# Sex differences in cardiac structure and function following ST-segment elevation myocardial infarction

**DOI:** 10.1038/s41598-026-52993-8

**Published:** 2026-05-19

**Authors:** Kim W. L. M. Ricken, Kyriakos Panaou, Chris Lenselink, Lawien Al Ali, Pim van der Harst, Iwan C. C. van der Horst, Gabija Pundziute-Do Prado, Adriaan A. Voors, Erik Lipsic

**Affiliations:** 1 Department of Cardiology, University Medical Center Groningen, Hanzeplein 1, Groningen, 9713 GZ The Netherlands; 2 Department of Intensive Care Medicine, Maastricht University Medical Center, Maastricht, the Netherlands; 3 Cardiovascular Research Institute (CARIM), Maastricht University, Universiteitssingel 50, Maastricht, 6229 ER the Netherlands

**Keywords:** Sex Differences, STEMI, Cardiac remodelling, Echocardiography, Cardiology, Diseases, Medical research, Physiology

## Abstract

Post-STEMI cardiac remodelling represents an important determinant of long-term outcomes, yet sex-specific differences in this process remain poorly understood. While disparities between women and men are well-established in the presentation, treatment, and outcomes of both STEMI and heart failure, the extent to which cardiac structure and function evolve differently between the sexes following a myocardial infarction has received limited attention. This study investigated sex-based differences in cardiac remodelling among patients enrolled in the GIPS-III trial, a randomized controlled trial comparing metformin to placebo in STEMI patients without diabetes. Transthoracic echocardiography was performed during the initial hospitalization and repeated at four months, allowing for a detailed assessment of structural and functional changes over time. The analysis included 379 patients, of whom 95 were women and 284 were men. Women presented with a higher prevalence of hypertension, lower hemoglobin levels, and markedly elevated NT-proBNP concentrations both on admission and at 24 hours. At both time points, women demonstrated smaller left ventricular diameters and volumes, a higher E/e’ ratio, and a greater left ventricular ejection fraction compared to men. Despite these baseline differences, the trajectories of echocardiographic parameters over time did not differ meaningfully between sexes. Adverse left ventricular remodelling, defined as a 20% or greater increase in left ventricular end-diastolic volume, occurred at comparable rates in women and men. Left ventricular geometry at four months, encompassing normal geometry, concentric or eccentric hypertrophy, and concentric remodelling, was similarly distributed across sexes. These findings suggest that while women and men differ in their baseline cardiac characteristics following STEMI, the underlying remodelling process appears to follow a broadly similar course, which has relevant implications for how post-STEMI monitoring and management strategies might be applied across sexes.

**Supplementary Information:**

The online version contains supplementary material available at 10.1038/s41598-026-52993-8.

## Introduction

Ischemic heart disease is the leading cause of morbidity and mortality in high-income countries for both women and men^1^. In recent times, the disparities between men and women in the context of acute coronary syndrome (ACS) have garnered considerable attention. There is extensive literature regarding sex disparities in clinical presentation, treatment, and outcomes after ST-elevation myocardial infarction (STEMI) in the current era of early percutaneous coronary intervention (PCI).

Notably, women tend to present less frequently with classical anginal symptoms^2,3^ and exhibit a higher propensity to present without chest pain^3^. Furthermore, women experience longer patient and doctor delays before receiving medical attention^2,4–6^ are less likely to undergo PCI^7,8^ and receive less guideline-directed therapy after STEMI^9–11^. Consequently, various studies reported worse clinical outcomes following STEMI in women^6,7,9–11^.

Less data is available on the change in cardiac structure and function after acute myocardial infarction. Preclinical research in rats examining the response after myocardial infarction (MI) demonstrated that female rats developed more concentric hypertrophy following volume and pressure overload, whereas male rats exhibited more dilated and eccentric hypertrophy^12,13^. These findings align with clinical data from studies on aortic stenosis, hypertension, and heart failure, which showed different patterns of remodelling between women and men^14–18^. However, robust clinical data in the context of a STEMI are lacking. Additionally, there is a near absence of clinical data regarding functional changes in STEMI patients. Therefore, we studied sex disparities in structural and functional echocardiographic parameters during hospitalization and their changes over a four-month period post-STEMI.

## Methods

### Study population and design

The current study was a post hoc analysis of Glycometabolic Intervention as an adjunct to primary percutaneous intervention in ST-elevation myocardial infarction (GIPS-III), a single-center, randomized, double-blind, placebo-controlled trial investigating the effects of metformin on left ventricular ejection fraction (LVEF) in non-diabetic patients presenting with first-time STEMI^19–22^. The full study protocol and trial design have been described in detail previously^19^; primary outcomes have been reported elsewhere^20^. In brief, a total of 379 STEMI patients treated with primary PCI at the University Medical Center Groningen between Jan 1, 2011, and May 26, 2013, were included in the study. Exclusion criteria included age < 18 years, prior myocardial infarction, diabetes, indication for coronary artery bypass grafting, and severe renal impairment. Participants were randomized to receive either metformin (500 mg twice daily) or placebo. Metformin did not improve LVEF compared to placebo at four months^20^. In addition, no significant difference in the incidence of major adverse cardiac events (MACE) or new-onset diabetes was observed between the treatment groups over a two-year follow-up period^21^. The trial is registered at ClinicalTrials.gov (NCT01217307; registered October 7, 2010) and was approved by the Medical Ethics Review Committee of the University Medical Center Groningen (METc UMCG; ID NL-OMON39113) on August 13, 2010, in accordance with national regulatory requirements. The study was conducted in accordance with the Declaration of Helsinki^23^, and all participants provided written informed consent.

**Fig. 1:**
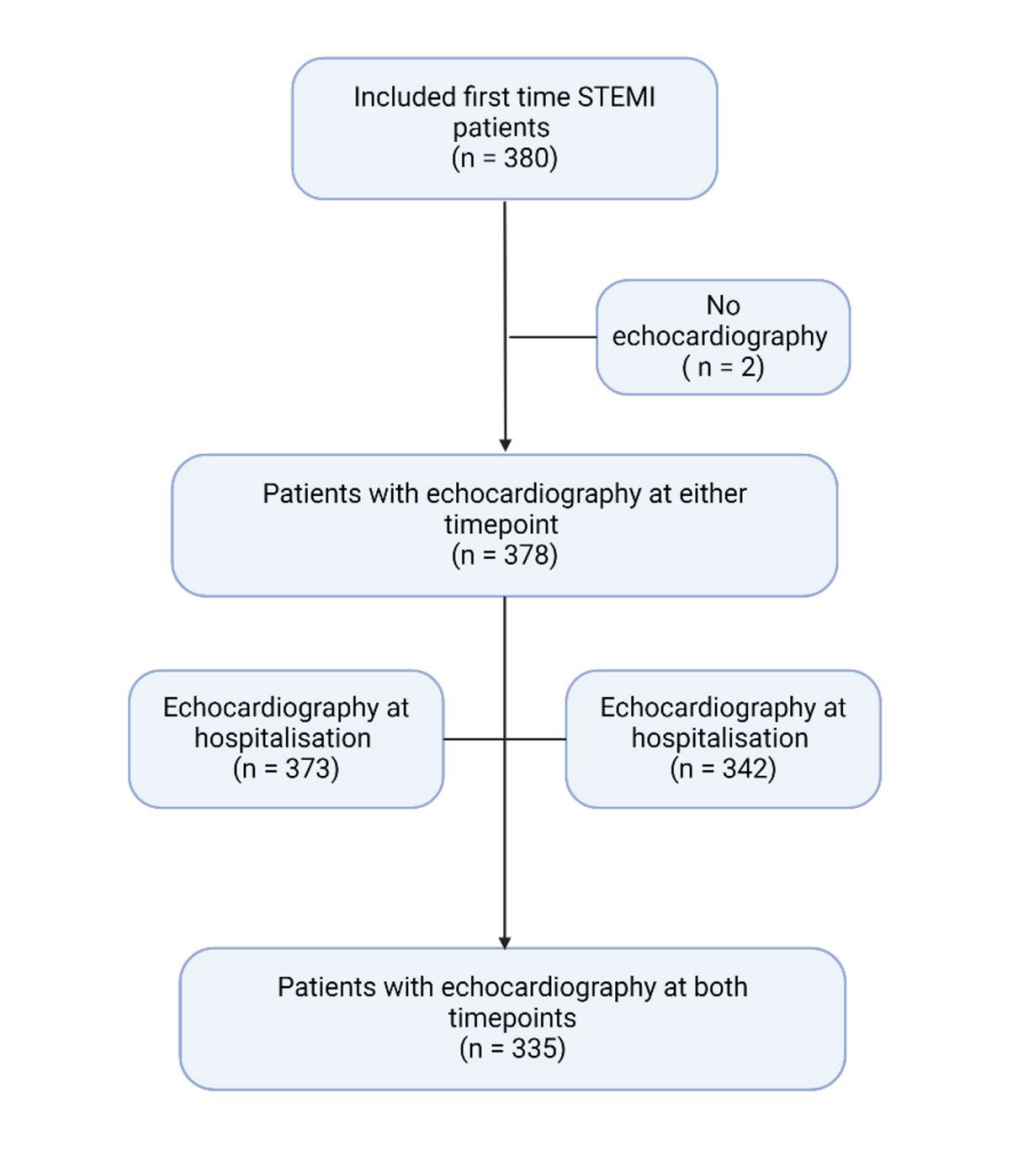
Flowchart of echocardiography availability.

The present study included all patients with available echocardiograms at either hospital admission or 4-month follow-up (*n* = 378). Only patients with echocardiograms at both time points were included for analyses examining the differences between the two time points, as shown in Fig. 1. Information regarding the missing values for each echo parameter is provided in ***Supplementary Table 1***.

### Patient and public involvement

Patients or the public were not involved in the design, conduct or dissemination plans of our research.

### Echocardiography

Transthoracic echocardiography was performed in the left lateral decubitus position using a Vivid 7 echo system (GE Healthcare, Horton, Norway) during hospitalization for STEMI and at the 4-month follow-up visit. All echocardiographic data were digitally stored in the DICOM format and analysed offline using EchoPAC BT version 10 (GE Healthcare, Horton, Norway) and Automated Function Imaging (AFI) software implemented in the EchoPAC Plug-in (version 204, GE Healthcare, Chicago, IL, USA). These are commercially available software packages from GE Healthcare (https://www.gehealthcare.com). All echocardiographic analyses were performed by certified echocardiographers at an independent core laboratory (Groningen Imaging Core Laboratory, Groningen, the Netherlands). Operators performing the analyses were blinded to treatment allocation and clinical information. Echocardiograms were interpreted in accordance with contemporary guidelines^24–26^. Where appropriate, echocardiographic measurements were indexed to body surface area (BSA) using the Du Bois and Du Bois formula^27^. The following structural measurements were evaluated: Left ventricular (LV) interventricular septal and posterior wall thickness, LV end-diastolic diameter (LVEDD), and LV end-systolic diameter (LVESD). LV mass was estimated from linear dimensions according to the method described by Devereux et al.^28^. Cardiac chamber volumes were quantified in accordance with the ASE/EACVI recommendations for cardiac chamber quantification in adults^26^. Simpson’s biplane volumetric parameters were also measured, including LV end-diastolic volume (LVEDV) and LV end-systolic volume (LVESV). Left atrial volume (LAV) was measured using the area-length method. The left atrial volume index (LAVI) was also determined.

Functional parameters included LVEF, the ratio of early transmitral flow to early mitral annular velocity (E/e’), left atrial (LA) and Global Longitudinal Strain (GLS), and wall motion score index (WMSI). LVEF was calculated as LVEF = (LVEDV-LVESV)/LVEDV × 100%. Tissue color Doppler was used to measure early diastolic tissue velocities (e’) from both the septal and lateral walls^26^, and the mean e’ was calculated as (e’ septal + e’ lateral)/2. E/e’ ratio was calculated as E/mean e’. The reported values represent the mean of three heartbeats at the end of expiration. A single operator measured LA strain, LV global, and regional longitudinal strain according to the relevant European Association of Cardiovascular Imaging (EACVI) consensus paper^24^. For LA strain, measurements were performed in an Apical Four-Chamber (A4CH) view. Regarding the GLS, patients with missing or poor-quality A4CH, Apical Two-Chamber (A2CH), or Apical Long Axis (APLAX) views at hospitalization or four-month follow-up were excluded. For each patient, LV segments were designated as ‘affected’ or ‘not affected’ by the infarction based on the recorded culprit coronary artery segment. The average GLS of the affected and non-affected segments were calculated in each patient.

### Definitions

Adverse remodelling was defined as a 20% increase in LVEDV at 4 months compared with the hospitalization echo^29^.

In accordance with current guidelines, eccentric hypertrophy was defined as left ventricular mass index (LVMI) > 95 g/m2 in women and > 115 g/m2 in men with relative wall thickness (RWT) ≤ 0.42; concentric hypertrophy was defined as LVMI > 95 g/m2 in women and > 115 g/m2 in men with RWT > 0.42; concentric remodelling was defined as LVMI ≤ 95 g/m2 in women and ≤ 115 g/m2 in men with RWT > 0.42^26^. These geometric patterns were assessed based on the 4-month echocardiography.

### Statistical analysis

Continuous variables were reported as mean ± standard deviation or median (interquartile range) for normally distributed and skewed data. Categorical variables were presented as numbers (percentages). Skewed data were log-transformed. Differences between groups were tested using the two-tailed t-test for normally distributed data and the Mann-Whitney U test for skewed data. For categorical variables, the chi-squared test was used. Paired t-tests were used to assess differences between two time points for normally distributed data and the Wilcoxon rank-sum test for skewed data. A significance level of 0.05 was used for two-tailed tests.

## Results

### Patient population

A total of 379 patients, consisting of 95 women and 284 men, were included in the GIPS-III study, with a mean age of 58.3 ± 11.7 years. During hospitalization, echocardiographic data were available for 373 patients, including 92 women and 281 men. At the four-month follow-up, echocardiography results were available for 342 patients, of which 76 were women, and 266 were men included in the analysis. At both time points, echocardiography data were available for 335 patients, including 76 women and 259 men **(**Fig. 1**)**.

As shown in Table 1, women had a significantly higher prevalence of hypertension (*p* = 0.004), lower hemoglobin levels on admission (*p* < 0.001), higher HbA1c levels on admission (*p* = 0.025), and higher NT-proBNP levels both on admission (*p* < 0.001) and after 24 h (*p* < 0.001). There were no statistically significant differences in infarct-related factors, such as ischemia time, TIMI flow before and after PCI, and myocardial blush score or culprit vessels between the sexes. Additionally, enzymatic infarct size, as measured by peak CK-MB, did not differ between women and men. Discharge medication was also the same, except for statins, which were prescribed less often in women than in men (90.7% vs. 98.1%) **(Supplementary Table 2)**.

**Table 1:** Patient characteristics.

Characteristics	Total population	Men	Women	*p*-value
Number of patients	379	284	95	
Age, years	58.3 ± 11.7	57.8 ± 11.6	59.9 ± 11.9	0.14
Ethnicity				0.60
- Caucasian	366 (96.3%)	273 (95.8%)	93 (97.9%)	
- Asian	10 (2.63%)	9 (3.16%)	1 (1.05%)	
- Black	4 (1.05%)	3 (1.05%)	1 (1.05%)	
Body weight, kg	84.2 ± 14.3	86.8 ± 13.4	76.2 ± 13.9	**< 0.001**
Height, cm	177 ± 9.03	180 ± 7.39	167 ± 6.38	**< 0.001**
BMI, kg/m^2^	26.6 (24.2;29.2)	26.5 (24.4;29.0)	27.2 (24.1;29.4)	0.77
Blood pressure, mmHg				
- Systolic	133 (120;147)	132 (120;146)	137 (120;154)	0.17
- Diastolic	83.0 (74.0;94.0)	84.0 (75.0;95.0)	80.0 (73.0;91.0)	0.15
*Cardiovascular risk factors and patient history*				
Hypertension	113 (29.7%)	73 (25.6%)	40 (42.1%)	**0.004**
Hypercholesterolemia	239 (62.9%)	181 (63.5%)	58 (61.1%)	0.76
Smoking				0.071
- No	106 (27.9%)	76 (26.7%)	30 (31.6%)	
- Past	65 (17.1%)	56 (19.6%)	9 (9.47%)	
- Current	209 (55.0%)	153 (53.7%)	56 (58.9%)	
Family history of CVD < 65 years	127 (33.4%)	88 (30.9%)	39 (41.1%)	0.090
*Infarct related factors*				
Ischemia time, min	161 (109;249)	155 (109;245)	177 (110;266)	0.22
TIMI flow grade pre-PCI ≤ 1	236 (62.1%)	174 (61.1%)	62 (65.3%)	0.54
TIMI flow grade post-PCI < 3	34 (8.95%)	24 (8.42%)	10 (10.5%)	0.68
Myocardial blush grade ≤ 1 post-PCI	40 (10.6%)	27 (9.57%)	13 (13.7%)	0.35
Culprit:				0.61
- LAD	147 (38.7%)	110 (38.6%)	37 (38.9%)	
- RCA	169 (44.5%)	124 (43.5%)	45 (47.4%)	
- RCx	64 (16.8%)	51 (17.9%)	13 (13.7%)	
*Laboratory measurements – pre-PCI*				
Hemoglobin, mmol/L	8.95 (8.40;9.40)	9.10 (8.70;9.50)	8.40 (8.00;8.90)	**< 0.001**
Troponin T, ng/L	50.0 (23.0;136)	49.0 (25.0;133)	50.0 (22.0;145)	0.60
CK, U/L	130 (83.0;209)	138 (93.0;227)	100 (71.5;179)	**< 0.001**
CKMB, U/L	161 (68.5;326)	163 (76.0;335)	142 (52.0;301)	0.37
NT-proBNP, ng/L	81.0 (40.0;191)	67.0 (36.0;156)	132 (66.0;274)	**< 0.001**
eGFR, ml/min/1.37m^2^	92.3 ± 21.1	93.3 ± 20.4	89.3 ± 23.0	0.14
Creatinine, µmol/L	72.0 (62.0;82.0)	76.0 (67.0;85.0)	59.0 (54.0;69.5)	**< 0.001**
Urea, mmol/L	5.65 (4.70;6.62)	5.70 (4.80;6.60)	5.50 (4.60;6.65)	0.42
Potassium, mmol/L	3.70 (3.50;4.00)	3.80 (3.50;4.00)	3.70 (3.40;4.00)	**0.017**
Sodium, mmol/L	140 (139;141)	140 (139;141)	140 (139;142)	0.45
Glucose, mmol/L	8.20 (7.00;9.57)	8.40 (7.10;9.55)	8.00 (6.80;9.60)	0.68
HbA1c, %	5.80 (5.60;6.00)	5.80 (5.60;6.00)	5.90 (5.62;6.10)	**0.021**
HbA1c, mmol/mol	40.0 (38.0;42.0)	39.0 (37.0;42.0)	41.0 (38.0;43.0)	**0.025**
Total cholesterol, mmol/L	5.30 (4.70;6.00)	5.30 (4.70;6.00)	5.35 (4.80;6.00)	0.97
LDL cholesterol, mmol/L	3.80 (3.20;4.40)	3.80 (3.20;4.50)	3.70 (3.20;4.30)	0.47
HDL cholesterol, mmol/L	1.10 (0.90;1.30)	1.10 (0.90;1.30)	1.30 (1.10;1.50)	**< 0.001**
*Laboratory measurements – during admission*				
Peak Troponin T, ng/L	2880 (1107;6288)	2910 (1174;6412)	2587 (923;5509)	0.45
Peak CK, U/L	1347 (602;3078)	1360 (666;3161)	1206 (394;2911)	0.23
Peak CKMB, U/L	161 (68.5;326)	163 (76.0;335)	142 (52.0;301)	0.37
NT-proBNP, ng/L	902 (451;1614)	784 (407;1410)	1322 (740;1974)	**< 0.001**

Abbreviations: BMI, Body Mass Index; TIMI flow grade, Thrombolysis in Myocardial Infarction flow grade; LAD, Left Anterior Descending Coronary Artery; RCA, Right coronary artery; RCx, ramus circumflexis; CK, Creatine Kinase; CKMB, Creatine Kinase-MB isoenzyme; NT-proBNP, N-terminal pro–B-type natriuretic peptide; eGFR, estimated glomerular filtration rate; HbA1c, Glycated hemoglobin; LDL, low-density lipoprotein; HDL, high-density lipoprotein.

### Cardiac structure

At baseline (during hospitalization) and after four months, there were significant differences in echocardiographic parameters between women and men (Table 2). Women had smaller left ventricular systolic and diastolic diameters (p = < 0.01) and volumes (*p* < 0.01). However, the LAVI did not differ between women and men at either time points (*p* = 0.91 and *p* = 0.39). The absolute wall thickness was also lower in women than in men, although the RWT was not significantly different between the sexes (*p* = 0.42).

**Fig. 2:**
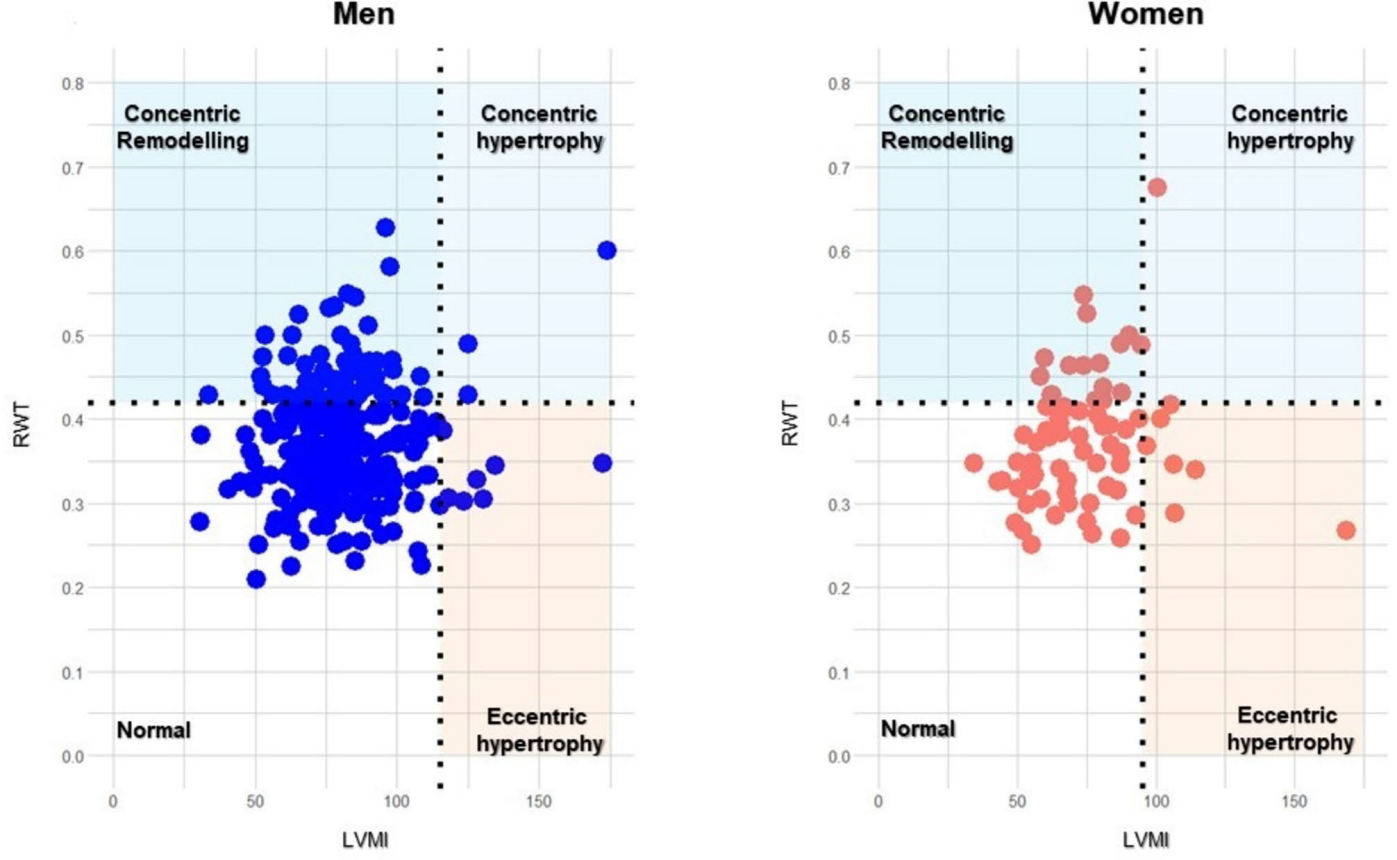
Scatterplot illustrating the distribution of different patterns of left ventricular geometry between sexes 4-months post-STEMI. Abbreviations: LVMI, left ventricular mass index; RWT, relative wall thickness.

As shown in Fig. 2 and **Supplementary Table 3**, there were no significant differences in geometry patterns between the sexes on the four-month echocardiogram. Most patients (67% women and 76% men) had a normal geometry at the four-month echo. Concentric hypertrophy was present in only 1% of both women and men. There was a slight difference in eccentric hypertrophy (10% in women and 3% in men), although this difference was not statistically significant (*p* = 0.21).

### Cardiac function

Women had a higher LVEF at both time points, although this was only statistically significant at the four-month echo (p = 0.005). Women also had a significantly higher E/e’ ratio compared to men on both the first echo (8.53 versus 7.35, *p* = 0.002) and the four-month echo (8.73 versus 7.56, *p* < 0.001). The WMSI, absolute GLS, GLS in affected and non-affected regions, and LA reservoir and conduit strain did not differ significantly between sexes at any time point (Table 2). The initial LA contraction strain was significantly lower in women than in men (-11% versus − 13%, *p* = 0.04). Although this trend continued on the four-month echocardiogram, it was no longer significant (*p* = 0.07).

### Changes in cardiac structure and function between hospitalization and 4 months

As shown in Table 3, no significant difference in the change in any structural or functional echocardiographic parameters between the two time points (hospitalization and four months) was observed. Furthermore, adverse remodelling did not differ between the sexes, with an incidence of 21.7% in women and 23.2% in men (*p* = 0.99).

**Table 2:** Structural and functional echocardiographic parameters at hospitalization and 4-month follow-up.

Variable	During hospitalization			At 4 months			Change between both visits		
	Men (*n* = 281)	Women (*n* = 92)	*P*-value	Men (*n* = 266)	Women (*n* = 76)	*P*-value	Men (*n* = 259)	Women (*n* = 76)	*P*-value
Time until echo, days	1.00 (1.00, 2.00)	1.00 (1.00, 2.00)	0.34	123 (117, 128)	124 (119, 128)	0.45	121 (113, 126)	121 (115, 126)	0.82
**Structural parameters**									
**Diameters**									
LVEDD, mm	49.0 (46.0, 53.0)	46.0 (43.0, 49.0)	**< 0.01**	50.0 (47.0, 54.0)	47.0 (43.5, 50.0)	**< 0.01**	1.0 (-1.0, 4.0)	2.0 (-1.0, 5.0)	0.13
LVESD, mm	34.0 (30.0, 37.0)	30.0 (28.0, 33.5)	**< 0.01**	33.0 (30.0, 37.0)	31.0 (27.0, 34.0)	**0.01**	0.0 (-4.0, 3.3)	1.0 (-2.0, 4.0)	0.11
**Volumes**									
LVEDV, ml	126 (98.5, 146)	96.5 (79.5, 113)	**< 0.01**	121 (100, 150)	95.0 (85.0, 111)	**< 0.01**	2.0 (-20.50, 24.0)	-4.5 (-10.0, 12.8)	0.54
LVESV, ml	65.0 (49.0, 80.0)	48.0 (35.0, 63.2)	**< 0.01**	60.0 (42.5, 76.5)	41.0 (32.0, 53.0)	**< 0.01**	-5.0 (-18.0, 11.5)	-7.0 (-15.3, 3.0)	0.35
LAV, ml	54.0 (45.0, 67.0)	48.0 (41.0, 58.0)	**0.007**	57.0 (48.0, 68.0)	54.0 (42.5, 64.5)	0.065	-3.0 (-12.0, 7.0)	-5.0 (-14.0, 5.0)	0.43
LAVI, ml/m2	26.4 (22.2, 32.2)	25.4 (23.2, 31.5)	0.91	27.4 (23.7, 33.4)	28.5 (24.0, 34.7)	0.39	1.4 (-3.3, 5.8)	2.7 (-2.9, 7.4)	0.29
Adverse remodelling, %							36 (23.2)	10 (21.7)	0.99
**Wall thickness**									
Septal wall, mm	10.0 (9.0, 11.0)	9.0 (8.0, 10.0)	**0.010**	9.0 (8.0, 10.0)	8.5 (8.0, 10.0)	**0.001**	0.0 (-1.0, 0.0)	0.0 (-1.0, 0.0)	0.76
Posterior wall, mm	9.0 (8.0, 10.0)	9.0 (8.0, 9.8)	**0.004**	9.0 (8.0, 10.0)	9.0 (8.0, 9.0)	0.067	0.0 (-1.0, 1.0)	0.0 (-1.0, 1.0)	0.10
Relative wall thickness	0.43 (0.38, 0.49)	0.43 (0.39, 0.49)	0.42	0.37 (0.33, 0.41)	0.37 (0.32, 0.41)	0.94	-0.07 (-0.12, 0.01)	-0.06 (-0.11, -0.01)	0.95
**Functional parameters**									
**Left ventricle**									
LVEF, %	48.0 (42.0, 55.0)	50.0 (44.0, 55.0)	0.14	52.6 (46.3, 58.4)	57.1 (47.7, 62.8)	**0.005**	3.63 (-2.52, 11.2)	7.74 (0.44, 12.6)	0.37
WMSI	1.31 (1.13, 1.63)	1.25 (1.10, 1.69)	0.84	1.19 (1.00, 1.44)	1.12 (1.00, 1.33)	0.17	-0.10 (-0.25, 0.00)	-0.06 (-0.22, 0.00)	0.63
E/e` ratio	7.35 (6.24, 8.88)	8.53 (6.58, 11.1)	**0.002**	7.56 (6.28, 8.78)	8.73 (7.31, 11.1)	**<0.001**	-0.02 (-1.46, 1.25)	0.62 (-0.88, 2.00)	0.096
GLS, %	-14.9 (-15.5, -14.4)	-15.7 (-17.0, -14.4)	0.25	-17.5 (-18.0, -16.8)	-18.4 (-19.3, -16.8)	0.25	-2.0 (-2.4, -1.5)	-1.8 (-3.0, -0.59)	0.77
GLS – affected region, %	-13.7 (-14.7, -12.6)	-12.9 (-15.8, -11.4)	0.93	-15.8 (-17.1, -15.3)	-17.6 (-18.6, -16.7)	0.19	-2.6 (-3.2, -1.6)	-3.4 (-5.1, -0.22)	0.93
GLS – unaffected region, %	-17.0 (-17.6, -16.00)	-17.6 (-20.0, -16.1)	0.07	-18.6 (-19.2, -18.3)	-19.2 (-21.6, -16.7)	0.28	-1.6 (-2.0, -0.88)	-1.4 (-2.6, 0.67)	0.49
**Left atrium**									
Reservoir strain, %	27.0 (22.5, 33.5)	26 (21.0, 32.0)	0.34	29.0 (24.0, 34.0)	29.0 (22.0, 34.0)	0.60	2.0 (-2.0, 5.0)	1.0 (-2.0, 6.0)	0.94
Conduit strain, %	-14.0 (-18.0, -11.0)	-14.0 (-19.0, -11.0)	0.60	-15.0 (-18.0, -12.0)	-15.0 (-19.0, -12.0)	0.74	0.0 (-3.0, 4.0)	-1.0 (-2.0, 3.0)	0.67
Contraction strain, %	-13.0 (-16.0, -9.0)	-11.0 (-14.0, -8.0)	**0.04**	-14.0 (-17.0, -11.0)	-13.0 (-16.3, -9.0)	0.07	-2 (-5.0, 1.0)	-1 (-5.0, 1.0)	0.92

Abbreviations: LVEDD, Left Ventricular End-Diastolic Diameter; LVESD, Left Ventricular End-Systolic Diameter; LAV, Left Atrial Volume; LAVI, Left Atrial Volume Index; LVEF, Left Ventricular Ejection Fraction; WMSI, Wall Motion Score Index; E/e’ ratio, Ratio of Early Diastolic Transmitral Flow Velocity to Early Diastolic Mitral Annular Velocity; GLS, Global Longitudinal Strain.

## Discussion

This study investigated sex differences in cardiac structure and function after STEMI. Although we observed the anticipated differences in patient characteristics and individual echocardiographic parameters between women and men, no sex disparities were noted in geometric patterns, adverse LV remodelling, or change in individual structural or functional echocardiographic parameters between hospitalization and 4 months after STEMI (Table 2).

Concerning the structural echocardiographic parameters, we found expected differences in individual parameters. Women had lower LV volumes and diameters than men, which is consistent with the literature and can be attributed to lower body size^30^. Based on preclinical research and clinical studies involving aortic stenosis, hypertension, and heart failure, which describe concentric hypertrophy and remodelling as being more prevalent in women, whereas dilated or eccentric hypertrophy/remodelling is more common in men, we anticipated finding similar patterns of remodelling after STEMI^12–18^. However, our findings did not indicate any differences in the geometric patterns of post-STEMI between sexes. One possible explanation could be the significant technical and therapeutic advancements in myocardial infarction management in the current era of early PCI, limiting myocardial damage and infarct size. In our study, most patients exhibited normal cardiac geometry 4 months post-STEMI. Another possible explanation could be that the analysis of geometric changes between the sexes has predominantly been conducted within the framework of (prolonged) LV volume or pressure overload. Chronic LV pressure and/or volume overload are prevalent in conditions such as aortic stenosis, heart failure, and hypertension, which can significantly affect remodelling patterns. In contrast, STEMI represents an acute pathological event rather than a scenario of prolonged overload. This acute process fundamentally differs from the chronic stress imposed by AoS, chronic heart failure, or hypertension and could, therefore, lead to differences in remodelling patterns.

Similar to our study, van der Bijl et al. (2020) found a similar incidence of adverse remodelling between women and men^31^. However, the prevalence of adverse remodelling in the overall patient cohort (48%) was higher than in our population (22.9%), with a comparable age distribution. This discrepancy could be attributed to several factors. In this study, only patients with first-time STEMI were included, whereas the study by van der Bijl et al. also included patients with a previous infarct (8% of their population). Additionally, our cohort did not include patients with diabetes, while van der Bijl et al. reported a 10% prevalence of diabetes. By focusing exclusively on first-time STEMI patients without diabetes, our study aimed to more precisely assess the impact of the infarct itself, minimizing potential confounding effects from comorbidities or pre-existing damage. Furthermore, the study by van der Bijl et al. had limited information on infarct severity, with only peak cardiac troponin T (cTnT) available. The peak cTnT level in the patient cohort was higher than in our cohort (3500 ng/L vs. 2880 ng/L), possibly suggesting larger infarcts. In addition, in the study by van der Bijl et al., men exhibited significantly higher cTnT levels than women (3,800 ng/L versus 2,900 ng/L, *p* < 0.001), whereas, in our study, the (enzymatic) infarct size was equivalent between women and men. Finally, van der Bijl et al. focused exclusively on structural LV parameters, which are relatively crude and late measures of LV remodelling, whereas our current study also emphasizes functional echocardiographic parameters.

A remarkable and consistent finding was a higher E/e’ ratio in women at hospitalization and after four months; however, the change in E/e’ ratio did not differ significantly between women and men. An earlier sub-study of GIPS-III focussed on adverse diastolic remodelling, demonstrated that the female sex was a predictor of a higher E/e’ ratio, observed both at the hospitalization echo and the 4-month echo, but not for the change in E/e’^22^. This finding was also reflected in our current analysis and could be attributed to the fact that women are older and have a higher prevalence of hypertension prior to STEMI. We found no statistically significant differences in the other functional parameters, WMSI and GLS.

### Limitations

Our cohort was predominantly male, reflecting a higher incidence of STEMI in men. However, ischemia-related parameters, enzymatic infarct size, and medical treatments were comparable between the sexes, ensuring a fair comparison. Women in our study had a higher prevalence of hypertension and higher LVEF at baseline. However, our interaction analysis for both hypertension and LVEF revealed no significant interactions, indicating that these differences did not substantially affect or confound the study outcomes.

The small number of women and the single-center nature of the study may have limited generalizability. Additionally, we only included patients with first-time STEMI without diabetes, with a relatively low risk of future events. This may limit its applicability to the broader STEMI population, especially those with recurrent events or diabetes. Furthermore, this study focused solely on post-STEMI remodelling and function, and the findings may not apply to non-ischemic remodelling or sex differences in those contexts.

The sample sizes for some parameters were small because of missing data and the limited group sizes (***Supplementary Table 1***). We retrospectively calculated the required sample size based on the observed median and IQR (***Supplementary Table 4***) and found that only minor and clinically insignificant differences between women and men could have been detected.

Finally, formal inter- and intra-observer variability analysis was not performed. All imaging data were analysed by a dedicated core laboratory, which standardizes image acquisition and analysis protocols and thereby reduces variability inherent to single-reader assessments; nevertheless, the absence of reported reproducibility coefficients remains a limitation.

### Clinical perspectives

Our study does not confirm our initial expectation that women show a different remodelling pattern than men after myocardial infarction. This implies that the acute nature of STEMI may result in more uniform remodelling responses between sexes compared to the more distinct patterns observed in chronic conditions such as hypertension, heart failure, or aortic stenosis. Therefore, it may be crucial to ensure the early and effective treatment of STEMI patients with prompt percutaneous coronary intervention (PCI) to prevent the progression of the acute event into a chronic condition that could eventually lead to remodelling and associated complications.

The most important difference between our study and previous studies is that our study is conducted in the era of early PCI, offering unique insights into how sex differences manifest in this modern treatment setting. These insights may differ from findings in earlier studies that did not account for rapid reperfusion strategies, which are now a standard of care. Of note, the residual left ventricular ejection fraction after STEMI was 52–57% in our study, which is much higher than previous studies. This is the result of a well-advanced rapid reperfusion strategy. This strategy might have caused the absence of differences in remodelling between men and women in our study.

Additionally, an important finding in our study is that there were no significant differences in the presentation, infarct-related factors, or medical treatment between men and women in our cohort. This contrasts with many other studies where such differences often confound sex-based analyses of outcomes. The absence of these confounding factors in our study enhances the relevance of our findings, as it allows for a more focused examination of sex-related cardiac remodelling and function in the context of STEMI, independent of these variables.

Furthermore, we observed no disparities in ischemia time and other infarction parameters. These findings indicate that increasing awareness of acute coronary syndromes in women, along with more consistent diagnostic and treatment practices across sexes, may contribute to comparable echocardiographic changes following STEMI. These observations challenge the notion of significant sex-related differences in post-infarction myocardial structure and function, while also encouraging further exploration of sex differences across the broader spectrum of myocardial infarction. The contrast between our results and prior findings may highlight the need for further investigation into whether the commonly cited differences in outcomes are driven by biological factors, disparities in clinical care, or other variables, such as timing or methods of follow-up. These contrasting findings underscore the complexity of the issue and suggest that more nuanced or stratified analyses may be necessary in future trials.

## Conclusions

Despite the differences between women and men in patient characteristics and individual echocardiographic parameters, no significant differences in geometric patterns, adverse LV remodelling, or changes in echocardiographic structural or functional parameters after STEMI were observed.

## Electronic Supplementary Material

Below is the link to the electronic supplementary material.

Supplementary Material 1

Supplementary Material 2

Supplementary Material 3

Supplementary Material 4

Supplementary Material 5

## Data Availability

The data underlying this article will be shared on reasonable request to the corresponding author.
