# Supplementary material for: Sex differences in cardiac structure and function following ST-segment elevation myocardial infarction
Source: Sci Rep. 2026 May 19;16:22756. doi: 10.1038/s41598-026-52993-8 (PMC13385960; doi:10.1038/s41598-026-52993-8)
Supplement: Supplementary file 1 — Supplementary Material 1 [file 41598_2026_52993_MOESM1_ESM.docx]

**Supplementary material**

**Supplementary Table S1.** Missing data per echocardiographic parameter.

| **Variable** | **During hospitalization** | | **At 4 months** | | **Change between both visits** | |
| --- | --- | --- | --- | --- | --- | --- |
|  | **Number of patients available** | **Missing (%)** | **Number of patients available** | **Missing (%)** | **Number of patients available** | **Missing (%)** |
| LVEDD | 350 | 7.9 | 337 | 11.3 | 318 | 16.3 |
| LVESD | 344 | 9.5 | 335 | 11.8 | 311 | 18.2 |
| LVEDV | 251 | 33.9 | 264 | 30.5 | 201 | 47.1 |
| LVESV | 251 | 33.9 | 264 | 30.5 | 201 | 47.1 |
| LAV | 303 | 20.3 | 310 | 18.4 | 256 | 32.6 |
| Adverse remodelling | - | - | - | - | 179 | 47.1 |
| Septal wall thickness | 352 | 7.4 | 336 | 11.6 | 318 | 16.3 |
| Posterior wall thickness | 348 | 8.4 | 337 | 11.3 | 316 | 16.8 |
| Relative wall thickness | 359 | 5.5 | 336 | 11.6 | 320 | 15.8 |
| LAVI | 303 | 20.3 | 310 | 18.4 | 256 | 32.6 |
| E/A ratio | 343 | 9.7 | 326 | 14.2 | 296 | 22.1 |
| E/e` ratio | 324 | 14.7 | 322 | 15.3 | 275 | 27.6 |
| LVEF | 372 | 2.1 | 264 | 30.5 | 261 | 31.3 |
| WMSI | 294 | 22.6 | 307 | 19.2 | 258 | 32.1 |
| LA reservoir strain | 288 | 24.2 | 277 | 27.1 | 222 | 41.6 |
| LA conduit strain | 288 | 24.2 | 277 | 27.1 | 222 | 41.6 |
| LA contraction strain | 288 | 24.2 | 277 | 27.1 | 222 | 41.6 |
| GLS | 186 | 51.1 | 179 | 52.9 | 179 | 52.9 |
| GLS – affected region | 186 | 51.1 | 179 | 52.9 | 179 | 52.9 |
| GLS – unaffected region | 186 | 51.1 | 179 | 52.9 | 179 | 52.9 |

**Supplementary Table S2. Medication 4 months after hospitalization.**

|  | Total population | Men | Women | p-value |
| --- | --- | --- | --- | --- |
| Number of patients | 379 | 284 | 95 |  |
| Beta-blockers | 340 (95.5%) | 255 (94.4%) | 85 (98.8%) | 0.132 |
| ACE inhibitors | 276 (77.5%) | 212 (78.5%) | 64 (74.4%) | 0.519 |
| Angiotensin receptor blockers | 45 (12.6%) | 34 (12.6%) | 11 (12.8%) | 1.000 |
| Diuretics | 73 (20.5%) | 51 (18.9%) | 22 (25.6%) | 0.236 |
| Coumarin derivatives | 26 (7.30%) | 19 (7.04%) | 7 (8.14%) | 0.917 |
| P2Y12 Inhibitors | 346 (97.2%) | 262 (97.0%) | 84 (97.7%) | 1.000 |
| Calcium channel blockers | 30 (8.43%) | 22 (8.15%) | 8 (9.30%) | 0.910 |
| Statins | 343 (96.3%) | 265 (98.1%) | 78 (90.7%) | **0.004** |

Abbreviations: ACE inhibitors, Angiotensin-Converting Enzyme Inhibitors; P2Y12 inhibitors, Purinergic Receptor Y12 Inhibitors.

**Supplementary Table S3.** Incidence of geometry patterns between sexes based on four-month echocardiography.

|  | Normal | Eccentric hypertrophy | Concentric remodelling | Concentric hypertrophy |
| --- | --- | --- | --- | --- |
| Men | 75.53 % *(n= 195)* | 3.09 % *(n = 8)* | 20.46 % *(n = 53)* | 1.16 % *(n = 3)* |
| Women | 66.67 % *(n = 48)* | 9.72 % *(n = 7)* | 22.22 % *(n = 16)* | 1.39 % *(n = 1)* |

**Supplementary Table S4.** Retrospective Sample Size Calculations for Parameters Assessed Between Men and Women

| **Variable** | **During hospitalization** | | | **At 4 months** | | | **Change between both visits** | | |
| --- | --- | --- | --- | --- | --- | --- | --- | --- | --- |
|  | **Observed p-value** | Required sample size Men | Required sample size Women | **Observed p-value** | Required sample size Men | Required sample size Women | **Observed p-value** | Required sample size Men | Required sample size Women |
| LVEDD | <0.001 | 172 | 57 | <0.001 | 172 | 57 | 0.182 | 1139 | 376 |
| LVESD | <0.001 | 97 | 32 | 0.001 | 388 | 128 | 0.105 | 1663 | 549 |
| LVEDV | <0.001 | 82 | 27 | <0.001 | 117 | 39 | 0.540 | 3093 | 1021 |
| LVESV | <0.001 | 105 | 35 | <0.001 | 101 | 33 | 0.791 | 6882 | 2271 |
| LAV | 0.007 | 425 | 140 | 0.065 | 1701 | 561 | 0.428 | 2855 | 942 |
| Adverse remodelling | - | - | - | - | - | - | 0.991 | 24589 | 8114 |
| Septal wall thickness | 0.010 | 127 | 42 | 0.001 | 506 | 167 | 0.764 | - | - |
| Posterior wall thickness | 0.004 | - | - | 0.067 | - | - | 0.099 | - | - |
| Relative wall thickness | 0.416 | - | - | 0.943 | - | - | 0.950 | 5346 | 1764 |
| LAVI | 0.908 | 3163 | 1044 | 0.388 | 2993 | 988 | 0.293 | 7246 | 2391 |
| E/A ratio | 0.191 | 1307 | 431 | 0.827 | 64058 | 21139 | 0.637 | 2450 | 808 |
| E/e` ratio | 0.002 | 464 | 153 | <0.001 | 332 | 110 | 0.096 | 641 | 212 |
| LVEF | 0.142 | 1337 | 441 | 0.005 | 356 | 117 | 0.501 | 767 | 253 |
| WMSI | 0.842 | 2197 | 725 | 0.172 | 1250 | 412 | 0.633 | 1236 | 408 |
| LA reservoir strain | 0.34 | 3828 | 1263 | 0.60 | - | - | 0.94 | 2025 | 668 |
| LA conduit strain | 0.60 | - | - | 0.74 | - | - | 0.67 | 1550 | 512 |
| LA contraction strain | 0.04 | 388 | 128 | 0.07 | 1139 | 376 | 0.92 | 1139 | 376 |
| GLS | 0.4 | 21384 | 7057 | 0.5 | 1307 | 431 | 0.5 | 4029 | 1330 |
| GLS – affected region | >0.9 | 2286 | 754 | 0.2 | 340 | 112 | >0.9 | 1721 | 568 |
| GLS – unaffected region | 0.07 | 2376 | 784 | 0.3 | 2376 | 784 | 0.5 | 2376 | 784 |
