# Supplementary material for: Sex differences in cardiac structure and function following ST-segment elevation myocardial infarction
Source: Sci Rep. 2026 May 19;16:22756. doi: 10.1038/s41598-026-52993-8 (PMC13385960; doi:10.1038/s41598-026-52993-8)
Supplement: Supplementary file 2 — Supplementary Material 2 [file 41598_2026_52993_MOESM2_ESM.pdf]

Original Investigation

Effect of Metformin on Left Ventricular Function After Acute Myocardial Infarction in Patients Without Diabetes
The GIPS-III Randomized Clinical Trial

Chris P. H. Lexis, MD; Iwan C. C. van der Horst, MD, PhD; Erik Lipsic, MD, PhD; Wouter G. Wieringa, MD; Rudolf A. de Boer, MD, PhD; Ad F. M. van den Heuvel, MD, PhD; Hindrik W. van der Werf, MD; Remco A. J. Schurer, MD; Gabija Pundziute, MD, PhD; Eng S. Tan, MD, PhD; Wybe Nieuwland, MD, PhD; Hendrik M. Willemsen, MD; Bernard Dorhout, PhD; Barbara H. W. Molmans, PharmD; Anouk N. A. van der Horst-Schrivers, MD, PhD; Bruce H. R. Wolffenbuttel, MD, PhD; Gert J. ter Horst, PhD; Albert C. van Rossum, MD, PhD; Jan G. P. Tijssen, PhD; Hans L. Hillege, MD, PhD; Bart J. G. L. de Smet, MD, PhD; Pim van der Harst, MD, PhD; Dirk J. van Veldhuisen, MD, PhD; for the GIPS-III Investigators

IMPORTANCE Metformin treatment is associated with improved outcome after myocardial infarction in patients with diabetes. In animal experimental studies metformin preserves left ventricular function.

OBJECTIVE To evaluate the effect of metformin treatment on preservation of left ventricular function in patients without diabetes presenting with ST-segment elevation myocardial infarction (STEMI).

DESIGN, SETTING, AND PARTICIPANTS Double-blind, placebo-controlled study conducted among 380 patients who underwent primary percutaneous coronary intervention (PCI) for STEMI at the University Medical Center Groningen, the Netherlands, between January 1, 2011, and May 26, 2013.

INTERVENTIONS Metformin hydrochloride (500 mg) (n = 191) or placebo (n = 189) twice daily for 4 months.

MAIN OUTCOMES AND MEASURES The primary efficacy measure was left ventricular ejection fraction (LVEF) after 4 months, assessed by magnetic resonance imaging. A secondary efficacy measure was the N-terminal pro-brain natriuretic peptide (NT-proBNP) concentration after 4 months. The incidence of major adverse cardiac events (MACE; the combined end point of death, reinfarction, or target-lesion revascularization) was recorded until 4 months as a secondary efficacy measure.

RESULTS At 4 months, all patients were alive and none were lost to follow-up. LVEF was 53.1% (95% CI, 51.6%-54.6%) in the metformin group (n = 135), compared with 54.8% (95% CI, 53.5%-56.1%) (P = .10) in the placebo group (n = 136). NT-proBNP concentration was 167 ng/L in the metformin group (interquartile range [IQR], 65-393 ng/L) and 167 ng/L in the placebo group (IQR, 74-383 ng/L) (P = .66). MACE were observed in 6 patients (3.1%) in the metformin group and in 2 patients (1.1%) in the placebo group (P = .16). Creatinine concentration (79 μmol/L [IQR, 70-87 μmol/L] vs 79 μmol/L [IQR, 72-89 μmol/L], P = .61) and glycated hemoglobin (5.9% [IQR, 5.6%-6.1%] vs 5.9% [IQR, 5.7%-6.1%], P = .15) were not significantly different between both groups. No cases of lactic acidosis were observed.

CONCLUSIONS AND RELEVANCE Among patients without diabetes presenting with STEMI and undergoing primary PCI, the use of metformin compared with placebo did not result in improved LVEF after 4 months. The present findings do not support the use of metformin in this setting.

TRIAL REGISTRATION clinicaltrials.gov Identifier: NCT01217307.

JAMA. 2014;311(15):1526-1535. doi:10.1001/jama.2014.3315
Published online March 31, 2014.

Supplemental content at jama.com

Author Affiliations: Author affiliations are listed at the end of this article.

Group Information: The Glycometabolic Intervention as Adjunct to Primary Coronary Intervention in ST-Segment Elevation Myocardial Infarction (GIPS-III) Investigators and Committee members are listed at the end of this article.

Corresponding Author: Iwan C. C. van der Horst, MD, PhD, Department of Critical Care, University Medical Center Groningen, University of Groningen, Hanzeplein 1, 9700 RB Groningen, the Netherlands (i.c.c.van.der.horst@umcg.nl).

Copyright 2014 American Medical Association. All rights reserved.
Downloaded from jamanetwork.com by Rijksuniversiteit Groningen user on 05/06/2026

In the Western world, approximately 1 of every 7 people with acute myocardial infarction will die of its consequences.[1,2] Patients presenting with ST-segment elevation myocardial infarction (STEMI) require immediate treatment with antithrombotic agents and primary percutaneous intervention (PCI) to restore coronary blood flow.[1-3] Timely reperfusion reduces myocardial damage and the risk of developing left ventricular dysfunction.[4,5] Still, STEMI results in left ventricular dysfunction in up to 50% of patients, and approximately 20% to 40% of patients develop heart failure sometime after STEMI.[6,7] Heart failure after STEMI is associated with a 3 to 4 times higher mortality risk.[7-10] Left ventricular dysfunction is regarded as the strongest predictor for adverse outcome after STEMI.[1]

Metformin, a biguanide often used in the treatment of diabetes, has been reported to have favorable effects on ventricular function.[11] Basic studies showed that metformin treatment is associated with an enhanced phosphorylation of AMP-activated protein kinase, inducing changes in intracellular pathways and altering mitochondrial function.[12] Interference with these pathways may ultimately result in improved systolic and diastolic function. Data from experimental studies have suggested that administration of metformin before and during ischemia-reperfusion might affect these protective pathways and preserve left ventricular function, independent of glycometabolic state.[13-15] Moreover, in observational studies of patients with acute myocardial infarction, concurrent treatment with metformin was associated with lower peak values of creatine kinase (CK), myocardial band of CK, and troponins and with improved survival after STEMI in patients with type 2 diabetes, compared with other antihyperglycemic strategies.[16-18]

The Glycometabolic Intervention as Adjunct to Primary Percutaneous Coronary Intervention in ST-Segment Elevation Myocardial Infarction (GIPS) III trial was designed to determine whether metformin preserves left ventricular function after STEMI in patients without diabetes.[11]

Methods

Study Population

All patients admitted to the University Medical Center Groningen between January 1, 2011, and May 26, 2013, via the STEMI protocol were considered eligible for this trial. The design of the study has been described in detail previously.[11] Briefly, inclusion criteria were age older than 18 years, the presence of STEMI, and primary PCI with implantation of at least 1 stent with a diameter of at least 3 mm resulting in Thrombolysis in Myocardial Infarction (TIMI) flow grade 2 or 3 post PCI. Major exclusion criteria were previous myocardial infarction, known diabetes, the need for coronary artery bypass graft surgery, severe renal dysfunction, and standard contraindications for magnetic resonance imaging (MRI).

Study Procedures

On admission, standard laboratory assessment including serum concentrations of CK, myocardial band of CK, creatinine, N-terminal pro brain natriuretic peptide (NT-proBNP), blood glucose, and glycated hemoglobin (HbA_{1c}) was performed. Standard physical examination parameters including blood pressure, heart rate, and body mass index were measured. Coronary angiography was performed using standard techniques. The choice and order of coronary intervention (ie, thrombus aspiration, balloon angioplasty, or stenting) was left to the discretion of the operator. During the PCI procedure, all patients provided verbal informed consent in the presence of an independent witness.

After arrival in the coronary care unit, patients were randomly assigned in a 1:1 ratio, using block randomization of 6 patients, to a 4-month treatment with either metformin hydrochloride (500 mg) or a visually matching placebo, both administered twice daily, blinded to patients and investigators. The study medication was started as soon as possible after PCI, with the aim of administering the first dose within 3 hours after PCI. The dose of metformin was chosen arbitrarily, based on our experience in the treatment of patients with type 2 diabetes, for whom this would be a typical starting dose.

Manufacturing and packaging including blinding was performed by Stichting Apotheek Haagse Ziekenhuizen, Den Haag, the Netherlands, according to the Good Manufacturing Practice standards of the European Union. Study drug adherence was assessed by tablet counts at the visits to the outpatient clinic. The duration of the treatment, 4 months, was based on the presumption that the majority of myocardial remodeling occurs over the course of this critical period.[19]

Following admission to the coronary care unit, patients provided written informed consent. All patients received standard medication according to current guidelines, were offered rehabilitation programs for myocardial infarction, and were given general advice on diet, smoking, and lifestyle changes according to a standardized protocol.[1] Patients were seen in the outpatient clinic 2 weeks, 7 weeks, and 4 months after discharge. Standard laboratory assessment was repeated at 4 months.

Study monitoring, data management, and validation were independently performed at the Trial Coordination Center (University Medical Center Groningen, the Netherlands). Detailed information is available in eMethods 1 in Supplement. The members of the steering committee were responsible for the design and conduct of the trial and the collection of the data. An end point adjudication committee blinded to allocation assessed all end points, and a data and safety monitoring board advised on whether the trial should be stopped because of clear evidence of harm. Members of the publication committee wrote all drafts of the manuscript and vouch for the accuracy and completeness of the reported data.

The contents of this article are consistent with the research protocol, and the data analysis was performed according to a prespecified analysis plan. The study protocol was in accordance with the Declaration of Helsinki and was approved by the local ethics committee (Groningen, the Netherlands) and national regulatory authorities.

Copyright 2014 American Medical Association. All rights reserved.
Downloaded from jamanetwork.com by Rijksuniversiteit Groningen user on 05/06/2026

Study Outcomes

The primary efficacy measure was left ventricular ejection fraction (LVEF), measured by MRI 4 months after infarction. Imaging was performed on a 3.0 Tesla whole-body MRI scanner (Achieva; Philips) using a phased array cardiac receiver coil. Electrocardiogram-gated cine steady state, free precession magnetic resonance images were acquired during repeated breath holds in contiguous short-axis slices of 1 cm covering the entire left ventricle. The endocardial borders were outlined in end-systolic and end-diastolic images. Left ventricular end-systolic volumes and left ventricular end-diastolic volumes were calculated using the summation of slice method multiplied by slice thickness. An independent core laboratory (Image Analysis Center, VU University Medical Center, Amsterdam, the Netherlands) evaluated the MRI scans and assessed the primary efficacy measure, blinded for treatment allocation and clinical patient data.

One of the principal secondary efficacy measures was the NT-proBNP concentration at 4 months. Before unblinding the study we prioritized the NT-proBNP levels at 4 months as the principal secondary efficacy measure, above other collected outcome measures, based on the strong evidence for NT-proBNP level as an independent predictor of clinical outcome.[20] Other secondary efficacy measures were the incidence, within 4 months after infarction, of the combined end point major adverse cardiac events (MACE [cardiovascular death, recurrent myocardial infarction, target-lesion revascularization]), and single efficacy and safety measures consisting of death, recurrent STEMI and non-ST-segment elevation myocardial infarction, recurrent coronary intervention (excluding interventions solely based on baseline, pretreatment angiography and clinical information), target-lesion revascularization, target-vessel revascularization, non-target-vessel revascularization, coronary artery bypass graft surgery, hospitalization for heart failure, hospitalization for chest pain, implantation of an implantable cardioverter-defibrillator, stroke, and lactic acidosis. The definitions used are available in eMethods 2 in Supplement.

Concentrations of glucose and HbA_{1c} were recorded at 4 months as additional efficacy measures, whereas creatinine concentration was recorded as an additional safety parameter.

Statistical Analysis

The study was designed with 80% power to detect a significant difference in LVEF between the 2 groups 4 months after STEMI, at a 2-sided significance level of 5%. Previous randomized trials in patients with acute myocardial infarction that showed reduced mortality and morbidity demonstrated concurrent absolute differences in LVEF of 3 percentage points or more.[21,22] Therefore, we considered this to be a feasible and clinically important difference. The SD of the effect measure was estimated to be 9%.[23] We calculated that 141 patients would need to be enrolled in each group. To allow an estimated 12% of patients to not undergo the primary end point measurement, we increased the initial total sample size to 320 patients. Actual dropout rates were close to 25%, resulting in the recommendation by the data and safety monitoring board to increase the total sample twice, first to 350 patients and then to 380 patients. To maintain statistical power to detect a clinically significant difference, these recommendations were acknowledged by the steering committee.

All analyses were performed according to a prespecified statistical analysis plan, which was finalized prior to unblinding of the randomization code (available from authors). Values for continuous variables that approximate a normal distribution are presented as mean (SD), and differences between groups were assessed by 2-tailed *t* test. Continuous variables not normally distributed are presented as medians with their interquartile ranges (IQRs). If normality could not be assumed, data underwent log transformation to convert it to a normal distribution. If indicated, differences in effect measurements and their 95% confidence intervals are presented between the control group and the metformin group. A sensitivity analysis using standard linear regression analysis was performed adjusted for age, sex, baseline NT-proBNP concentration, and myocardial blush grade to evaluate the robustness of the effect of treatment on the outcome parameters.

Post hoc, we explored multiple-imputation analysis (M = 100) as implemented in Stata under the *mi* command. We used ordered logistic (*olog*) regression to impute the 3 missing myocardial blush grade values. We used multivariate normal regression (*mvn*) for missing continuous variables (108 missing LVEF and 5 missing HbA_{1c} values), which uses a Bayesian iterative Markov Chain Monte Carlo method to impute missing values. We explored 2 strategies of imputing missing values: strategy A, based on age, sex, myocardial blush grade, and baseline NT-proBNP values (the variables used for the multivariate sensitivity analysis) and strategy B, based on all available Table 1 baseline variables (glucose and claudication were omitted because of colinearity). χ^2 Analysis or Fisher exact test was used to test differences between proportions of presence of a clinical secondary efficacy measure as adjudicated by the end point adjudication committee.

All reported *P* values are 2-sided, and $P < .05$ was considered to indicate a significant difference between groups. Analyses were performed with Stata version 12.0 (StataCorp).

Results

Study Population

During the enrollment period, between January 1, 2011, and May 26, 2013, 1473 patients were admitted to our hospital via the STEMI protocol (**Figure 1**). After inclusion and exclusion criteria were considered and verbal informed consent obtained, a total of 380 patients were enrolled in the study. Of the total sample, 191 patients were randomly allocated to the metformin treatment and 189 patients to placebo. Following admission to the coronary care unit, all patients provided written informed consent, except for 1 (randomized to placebo). This patient withdrew verbal consent and was excluded from analysis, leaving 379 patients in the final study.

Baseline characteristics at randomization were well balanced in the 2 treatment groups (**Table 1**). The median time from the onset of symptoms to first coronary intervention was

Copyright 2014 American Medical Association. All rights reserved.

Downloaded from jamanetwork.com by Rijksuniversiteit Groningen user on 05/06/2026

Figure 1. Flow of Patients Through the Glycometabolic Intervention as Adjunct to Primary Coronary Intervention in the ST-Segment Elevation Myocardial Infarction (GIPS-III) Trial

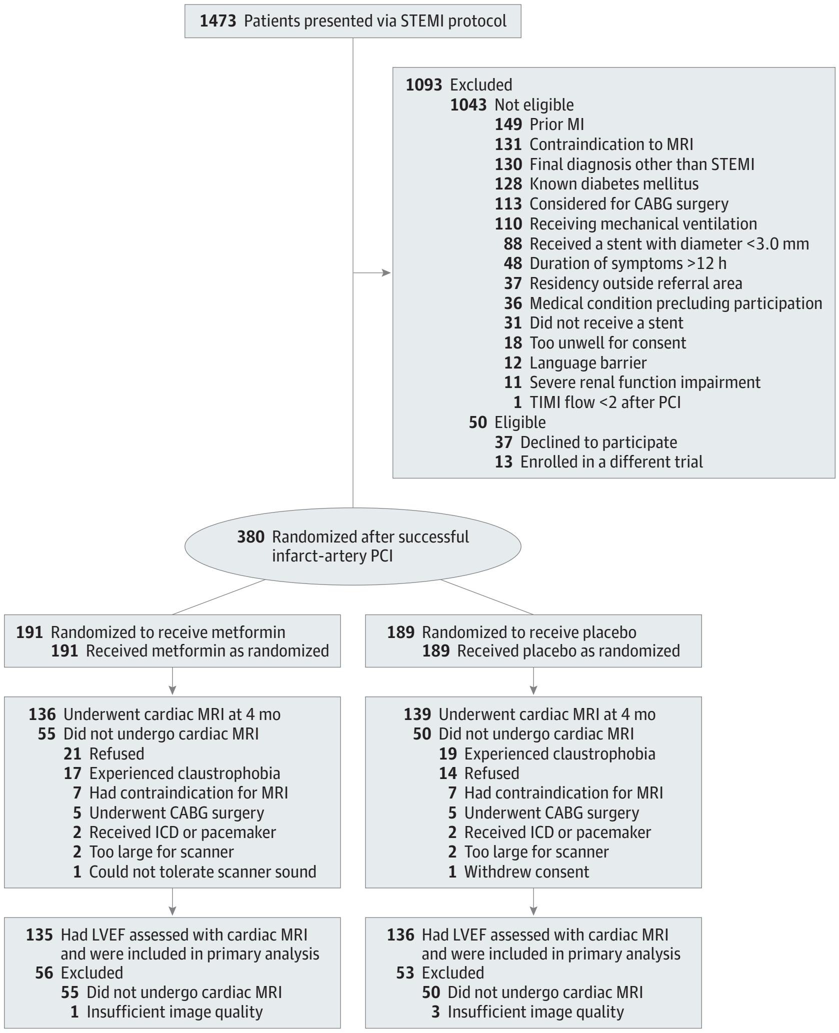

Patients could be excluded for more than 1 reason; the primary reason for exclusion in each case is shown. CABG indicates coronary artery bypass graft; ICD, implantable cardioverter-defibrillator; LVEF, left ventricular ejection fraction; MRI, magnetic resonance imaging; STEMI, ST-segment elevation myocardial infarction; TIMI, Thrombolysis in Myocardial Infarction; PCI, percutaneous coronary intervention.

161 minutes (IQR, 109-250). The median time of administration of the first dose of study treatment after first coronary intervention was 102 minutes in the metformin group (IQR, 81-133) and 100 minutes in the control group (IQR, 78-134) ($P = .27$). The median duration of exposure to study medication was 124 days in the metformin group (IQR, 119-125) and 124 days in the control group (IQR, 120-125) ($P = .14$). The rate of premature discontinuation of the study drug was similar between the metformin and placebo groups (16% vs 13%; $P = .42$). Further details on the medical therapy at discharge are reported in eTable 1 in Supplement.

Cardiac MRI Population

Four months after infarction, all patients were alive and none were lost to follow-up. Of these, 105 patients did not undergo MRI for various reasons (Figure 1). From among the 275 remaining patients, the MRI core laboratory considered 4 scans to be of insufficient quality. On average, patients with MRI data were on average younger, more often male, less often current smokers, had lower systolic blood pressure, less often had multivessel disease, and more often had better angiographic PCI outcome, as reflected by TIMI flow grade 3 after PCI (eTable 2 in Supplement). There were no baseline differences between the 2 treatment groups (eTable 3 in Supplement).

Primary End Point Measure

Left ventricular ejection fraction 4 months after randomization did not differ between the metformin group (53.1% [95% CI, 51.6%-54.6%]) and the placebo group (54.8% [95% CI, 53.5%-56.1%] $P = .10$) (**Table 2**). Additional sensitivity analysis, adjusting for age, sex, baseline NT-proBNP level, and myocardial blush grade suggested that this finding was robust and not driven by potential relevant baseline characteristics. Results were little changed when missing data were handled using multiple imputations. The treatment effect on LVEF was also similar in the prespecified subgroups, with the exception being the subgroup divided according to the median creatinine level (**Figure 2**). However, when creatinine levels were studied on

Copyright 2014 American Medical Association. All rights reserved.

Downloaded from jamanetwork.com by Rijksuniversiteit Groningen user on 05/06/2026

a continuous scale, no evidence for interaction with the effect of metformin was observed (interaction coefficient, 0.03 [95% CI, −0.11 to 0.17]; *P* = .69), suggesting that this finding is attributable to chance.

Secondary End Point Measures

The NT-proBNP concentration at 4 months after infarction, a principal secondary efficacy measure, was available in 320 patients (84.4%). The concentration of NT-proBNP was not dif-

Table 1. Baseline Characteristics

Characteristic	No. (%) Total (n = 379)	Metformin (n = 191)	Placebo (n = 188)
Age, mean (SD), y	58.8 (11.6)	58.7 (11.8)	58.8 (11.5)
Women	95 (25.1)	47 (24.6)	48 (25.5)
Body mass index, mean (SD)[a]	27.0 (3.8)	26.9 (3.8)	27.0 (3.9)
Race/ethnicity			
White	365 (96.3)	185 (96.9)	180 (95.7)
Asian	10 (2.6)	5 (2.6)	5 (2.7)
Black	4 (1.1)	1 (0.5)	3 (1.6)
Cardiovascular related history			
Hypertension	112 (30.0)	61 (31.9)	51 (27.1)
Dyslipidemia	239 (63.1)	111 (58.1)	128 (68.1)
Current smoking	209 (55.1)	108 (56.5)	101 (53.7)
Stroke	3 (0.8)	2 (1.0)	1 (0.5)
Peripheral artery disease	0	0	0
Previous PCI	3 (0.8)	1 (0.5)	3 (1.6)
Blood pressure, mean (SD), mm Hg			
Systolic	134 (23)	135 (23)	134 (24)
Diastolic	84 (15)	85 (14)	84 (15)
Heart rate, mean (SD), beats/min	76 (16)	75 (16)	77 (16)
Ischemia time, median (IQR), min	161 (109-250)	171 (110-272)	153 (108-234)
Single-vessel disease	258 (68.1)	122 (63.0)	136 (72.3)
Infarct-related artery			
Left main	0	0	0
Left anterior descending coronary artery	146 (38.5)	75 (39.3)	71 (37.8)
Left circumflex coronary artery	64 (16.9)	33 (17.3)	31 (16.5)
Right coronary artery	169 (44.6)	83 (43.5)	86 (45.7)
Infarct-related artery TIMI flow			
Preintervention grade			
0	208 (54.9)	99 (51.8)	109 (58.0)
1	27 (7.1)	14 (7.3)	13 (6.9)
2	66 (17.4)	45 (23.6)	21 (11.2)
3	78 (20.6)	33 (17.3)	45 (23.9)
Postintervention grade			
2	34 (9.0)	24 (12.6)	10 (5.3)
3	345 (91.0)	167 (87.4)	178 (94.7)
Myocardial blush grade			
0	10 (2.6)	6 (3.2)	4 (2.1)
1	29 (7.7)	20 (10.6)	9 (4.8)
2	74 (19.5)	35 (18.6)	39 (20.7)
3	263 (69.4)	127 (67.6)	136 (72.3)
Laboratory values at admission, median (IQR)			
CK, U/L	129 (83-210)	133 (87-260)	123 (82-181)
Myocardial band of CK, U/L	16 (13-25)	16 (13-29)	16 (12-23)
Creatinine, μmol/L	72 (62-82)	71 (61-84)	72 (63-80)
NT-proBNP, ng/L	81 (40-200)	83 (41-235)	79 (38-176)
Glucose, mmol/L	8.2 (7.0-9.6)	8.2 (7.0-9.4)	8.4 (7.2-9.8)
HbA_{1c}, %	5.8 (5.6-6.0)	5.8 (5.6-6.1)	5.8 (5.6-6.0)

Abbreviations: CK, creatine kinase; HbA_{1c}, glycated hemoglobin; IQR, interquartile range; NT-proBNP, N-terminal pro brain natriuretic peptide; PCI, percutaneous coronary intervention; TIMI, Thrombolysis in Myocardial Infarction.

[a] Calculated as weight in kilograms divided by height in meters squared.

Copyright 2014 American Medical Association. All rights reserved.

Downloaded from jamanetwork.com by Rijksuniversiteit Groningen user on 05/06/2026

Table 2. Outcomes at 4 Months

Outcome[a]	Total (n = 379)	Metformin (n = 191)	Placebo (n = 188)	P Value
**Primary end point, % (95% CI)**				
LVEF	53.9 (52.9-55.0)	53.1 (51.6-54.6)	54.8 (53.5-56.1)	.10
**Secondary end point, median (IQR)**				
NT-proBNP, ng/L	167 (72-390)	167 (65-393)	167 (74-375)	.66
Creatinine, μmol/L	79 (71-88)	79 (70-87)	79 (72-89)	.61
Glucose, mmol/L	5.7 (5.2-6.2)	5.7 (5.2-6.3)	5.6 (5.2-6.2)	.96
HbA_{1c}, %	5.9 (5.7-6.1)	5.9 (5.6-6.1)	5.9 (5.7-6.1)	.15
**Clinical outcomes, No. (%)**				
MACE[b]	8 (2.1)	6 (3.1)	2 (1.1)	.16
Death	0	0	0	1.00
Reinfarction	7 (1.8)	5 (2.6)	2 (1.1)	.26
STEMI	2 (0.5)	1 (0.5)	1 (0.5)	.99
Non-STEMI	5 (1.3)	4 (2.1)	1 (0.5)	.18
Stent thrombosis	3 (0.8)	2 (1.0)	1 (0.5)	.57
Ischemia-driven reintervention	15 (4.0)	8 (4.2)	7 (3.7)	.82
Target-lesion revascularization	4 (1.1)	3 (1.6)	1 (0.5)	.33
Target-vessel revascularization	3 (0.8)	1 (0.5)	2 (1.1)	.56
Non-target-vessel revascularization	8 (2.1)	4 (2.1)	4 (2.2)	.99
CABG surgery	1 (0.3)	1 (0.5)	0	.32
Hospitalization for heart failure	2 (0.5)	2 (1.0)	0	.16
Hospitalization for chest pain	24 (6.3)	14 (7.3)	10 (5.3)	.42
Implantable cardioverter-defibrillator implantation	3 (0.8)	2 (1.0)	1 (0.5)	.57
Stroke	1 (0.3)	0	1 (0.5)	.31
Diabetes	59 (15.6)	32 (18.5)	27 (15.3)	.56

Abbreviations: CABG, coronary artery bypass graft; HbA_{1c}, glycated hemoglobin; IQR, interquartile range; LVEF, left ventricular ejection fraction; MACE, major adverse cardiac events; non-STEMI, non–ST-segment elevation myocardial infarction; NT-proBNP, N-terminal pro-brain natriuretic peptide; STEMI, ST-segment elevation myocardial infarction.

[a] Definitions are available in eMethods 2 in Supplement.

[b] Death, reinfarction, or target-lesion revascularization.

ferent between the 2 groups, with a median of 167 ng/L (IQR, 65-393 ng/L) for the metformin group and 167 ng/L (IQR, 74-375 ng/L) for the placebo group (*P* = .66). This finding did not change in sensitivity analysis with adjustment for age, sex, baseline NT-proBNP concentration, and myocardial blush grade.

The combined MACE end point and individual end points—reinfarction, interventions, hospitalizations for heart failure and chest pain, implantations of implantable cardioverter-defibrillators, and stroke—up to 4 months are displayed in Table 2. No differences in MACE were observed between the metformin group compared with the placebo group (3% vs 1%, respectively; *P* = .16). There were also no differences in individual end points between the 2 treatment groups. There were no significant differences between the patients who underwent primary end point assessment and those who did not undergo MRI (eTable 4 in Supplement). The study medication was generally well tolerated. No case of lactic acidosis and impaired renal function was diagnosed during follow-up.

Concentrations of creatinine, glucose, and HbA_{1c} were available in 333 (87.8%), 328 (86.5%), and 317 (83.6%) patients, respectively. The creatinine concentration at 4 months was comparable between the metformin group (79 μmol/L [IQR, 70-87 μmol/L]) and the control group (79 μmol/L [IQR, 72-89 μmol/L]) (*P* = .61). Glucose levels measured at 4 months during the visit to the outpatient clinic were 5.7 mmol/L (IQR, 5.2-6.3 mmol/L) in the metformin group and 5.6 mmol/L (IQR, 5.2-6.2 mmol/l) in the placebo group (*P* = .96). The HbA_{1c} concentration at 4 months did not differ between groups, with a median of 5.9% (IQR, 5.6%-6.1%) for the metformin group and 5.9% (IQR, 5.7%-6.1%) for the placebo group (*P* = .15).

Discussion

In this double-blind, randomized, controlled trial, in patients without diabetes who underwent primary PCI for STEMI, treatment with 500 mg of metformin administered twice daily for 4 months did not have an effect on LVEF or NT-proBNP levels, compared with placebo. Because left ventricular function is currently regarded as the most important predictor of morbidity and mortality after STEMI, it is unlikely that metformin will have a significant effect on long-term outcome after STEMI in patients without diabetes.

To our knowledge, this study is the first to prospectively study the effects on left ventricular function of metformin compared with placebo as adjunct to optimal medical treatment. Our results did not confirm the effects observed in experimental studies.[13-15] In a nondiabetic rat model of myocardial infarction our group demonstrated that metformin was associated with a reduced infarct size of 22% and a relative improvement in LVEF of 52% as compared with placebo.[13] Another experimental study by Calvert et al[15] demonstrated that administration of metformin both prior to or during ischemia-reperfusion was effective in improving left ventricular function in a mouse model.

Copyright 2014 American Medical Association. All rights reserved.

Downloaded from jamanetwork.com by Rijksuniversiteit Groningen user on 05/06/2026

Figure 2. Estimated Effect of Metformin Compared With Placebo on Left Ventricular Ejection Fraction (LVEF) According to Prespecified Subgroups

Variable	No. of Patients		LVEF (95% CI), %		Difference in LVEF in Percentage Points (95% CI)	*P* for Interaction
	Metformin	Placebo	Metformin	Placebo		
Overall	135	136	53.09 (51.56-54.61)	54.80 (53.46-56.14)	-1.71 (-3.73 to 0.31)	
Sex						
Male	107	106	52.35 (50.76-53.94)	54.89 (53.37-56.41)	-2.54 (-4.73 to -0.36)	.11
Female	30	28	55.91 (51.70-60.12)	54.48 (51.45-57.50)	1.43 (-3.58 to -6.44)	
Age, y						
Below median (≤58.0)	71	65	53.81 (51.74-55.87)	54.69 (52.85-56.52)	-0.88 (-3.63 to 1.88)	.40
Above median (>58.0)	64	71	52.28 (49.98-54.58)	54.90 (52.92-56.89)	-2.62 (-5.61 to 0.38)	
BMI						
Below median (≤26.6)	69	67	52.21 (49.98-54.44)	54.77 (52.84-56.70)	-2.56 (-5.49 to 0.37)	.40
Above median (>26.6)	66	69	54.00 (51.89-56.11)	54.83 (52.92-56.74)	-0.83 (-3.64 to 1.99)	
Location of MI						
Anterior	55	56	51.32 (48.66-53.98)	53.26 (50.80-55.72)	-1.96 (-5.52 to 1.64)	.86
Nonanterior	80	80	54.30 (52.49-56.11)	55.88 (54.38-57.37)	-1.58 (-3.91 to 0.75)	
No. of diseased vessels						
1	93	102	53.24 (51.36-55.13)	55.30 (53.78-56.81)	-2.05 (-4.43 to 0.32)	.34
2 or 3	42	34	52.74 (50.06-55.42)	53.31 (50.37-56.26)	-0.57 (-4.41 to 3.27)	
Ischemic time, min						
Below median (≤156)	63	73	54.48 (52.32-56.54)	55.43 (53.85-57.01)	-0.95 (-3.55 to 1.66)	.54
Above median (>156)	72	63	51.87 (49.71-54.02)	54.07 (51.79-56.35)	-2.20 (-5.31 to 0.90)	
TIMI flow before PCI						
0 or 1	82	92	51.48 (49.52-53.44)	53.48 (51.70-55.27)	-2.01 (-4.64 to 0.62)	.97
2 or 3	53	44	55.58 (53.23-57.92)	57.55 (55.97-59.13)	-1.97 (-4.88 to 0.94)	
TIMI flow after PCI						
2	10	7	52.33 (46.64-58.03)	48.70 (38.76-58.64)	3.64 (-6.00 to 13.28)	.19
3	125	129	53.15 (51.54-54.75)	55.13 (53.80-56.46)	-1.98 (-4.05 to 0.08)	
Myocardial blush grade						
0 or 1	12	12	51.17 (45.88-56.46)	49.09 (42.11-56.06)	2.09 (-6.16 to 10.34)	.24
2 or 3	121	124	53.23 (51.59-54.86)	55.35 (54.04-56.66)	-2.13 (-4.21 to -0.05)	
Admission HbA1c						
Below median (≤5.8)	80	80	53.33 (51.33-55.33)	53.73 (51.79-55.69)	-0.39 (-3.16 to 2.37)	.16
Above median (>5.8)	54	55	53.08 (50.71-55.45)	56.43 (54.71-58.14)	-3.35 (-6.23 to -0.47)	
Admission glucose, mmol/L						
Below median (≤8.2)	64	73	54.51 (52.61-56.42)	55.27 (53.46-57.08)	-0.76 (-3.36 to 1.85)	.41
Above median (>8.2)	71	63	51.80 (49.46-54.14)	54.25 (52.22-56.29)	-2.45 (-5.56 to 0.66)	
Admission NT-proBNP, ng/L						
Below median (≤80)	68	69	54.08 (52.13-56.03)	56.12 (54.54-57.69)	-2.04 (-4.52 to 0.44)	.74
Above median (>80)	67	67	52.08 (49.71-54.46)	53.44 (51.26-55.62)	-1.36 (-4.55 to 1.83)	
Admission creatinine, µmol/L						
Below median (≤72)	70	66	51.24 (49.04-53.44)	55.30 (53.57-57.04)	-4.06 (-6.87 to -1.26)	.02
Above median (>72)	65	70	55.07 (53.03-57.12)	54.32 (52.26-56.38)	0.75 (-2.13 to 3.63)	

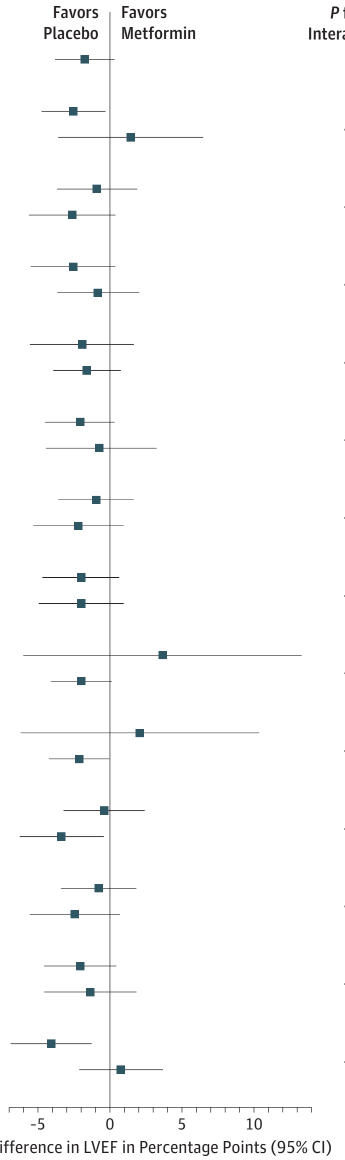

Estimation of the effect of metformin compared with placebo on LVEF according to prespecified subgroups. The estimated LVEF for the effect of metformin in the prespecified subgroups, as well as the absolute difference in LVEF for the effect of metformin per prespecified subgroup compared with the LVEF in the placebo group is displayed. BMI indicates body mass index; HbA_{1c}, glycated hemoglobin; MI, myocardial infarction; NT-proBNP, N-terminal pro-brain natriuretic peptide; PCI, percutaneous coronary intervention; TIMI, Thrombolysis in Myocardial Infarction.

Therefore, in our attempt to translate these experimental data into clinical situations, we included patients and started metformin immediately after PCI. Furthermore, because experimental data suggest that the effect of metformin on left ventricular function is not solely driven by glycemic control, we studied patients without known diabetes at inclusion.[13-15] In addition, in patients with established type 2 diabetes, metformin is indicated as standard treatment according to current guidelines; therefore, randomization of these patients to placebo is not appropriate.[24]

Earlier preclinical studies reporting on the protective effects of metformin were mainly performed in animals undergoing occlusion of the left main or the proximal left anterior descending coronary artery, resulting in a large area at risk.[13-15] To decrease heterogeneity of baseline LVEF, we only included patients presenting with their first acute myocardial infarction. Because of the exclusion of patients with prior myocardial infarction, as well as those with diabetes or those unable to provide informed consent, the baseline characteristics of our study population are somewhat favorable and lower-risk com-

Copyright 2014 American Medical Association. All rights reserved.

Downloaded from jamanetwork.com by Rijksuniversiteit Groningen user on 05/06/2026

pared with real-life practice. The exclusion of these high-risk categories might have influenced our findings.[8,9] However, in our subgroup analyses and in our sensitivity analyses adjusting for TIMI flow and myocardial blush grade, we did not find any evidence that the effect of metformin treatment was dependent on the area at risk.

Another potential reason for the lack of efficacy of metformin in our study might have been the time window between coronary occlusion and achieving effective plasma levels of metformin. On average, the administration of the first dose of study medication took place 101 minutes after coronary intervention. Considering a time to peak plasma concentration of metformin after oral administration of approximately 180 minutes, the average time between PCI and the achievement of effective plasma levels was approximately 4 hours.[25] Therefore, our study does not exclude a potential beneficial effect of effective plasma metformin levels during reperfusion or earlier.[26]

In a retrospective analysis, long-term metformin treatment in patients with diabetes was associated with lower peak levels of biochemical markers of myocardial infarct size and improved outcome compared with other glucose-lowering therapies.[18,27-29] However, data derived from nonrandomized, retrospective studies are susceptible to prescribing biases. Recently, metformin treatment (250 mg 3 times daily) in patients with the metabolic syndrome started 7 days prior to elective PCI resulted in both a smaller cardiac biomarker release after intervention and a favorable 1-year clinical outcome.[30] In experimental animal studies, the beneficial effects of metformin on left ventricular function and myocardial infarct size were similar when metformin was administered prior to or during ischemia-reperfusion.[15] In additional subgroup analyses, we did not observe differences in the treatment effect of metformin dependent on the timing of the first dose.

In addition, the lack of efficacy of metformin in our study might have been attributable to the dose of metformin administered. We studied metformin at a dosage of 500 mg twice daily because this is generally well tolerated and allows open-label treatment of metformin when diabetes is diagnosed after randomization. Another reason not to choose the highest possible dose are reports on metformin-induced lactic acidosis, especially in acute conditions related to renal insufficiency.[31,32] Patients with STEMI undergoing acute catheterization with contrast are indeed prone to the development of nephropathy, and safety data on high-dose metformin treatment were not available.[33] In this study, there was no evidence of lactic acidosis or impaired renal function. However, we cannot exclude a potential beneficial effect on LVEF of high-dose metformin treatment.

Four months of treatment with metformin (500 mg twice daily) did not have a large effect on glycemic control. Median glucose and HbA_{1c} levels did not differ between patients receiving metformin compared with placebo (P = .96 and P = .15, respectively). In the CAMERA (Carotid Atherosclerosis: Metformin for Insulin Resistance) study, treatment of patients with coronary artery disease but without diabetes using metformin (850 mg twice daily for 18 months) resulted in a small but significant difference of −0.13% in HbA_{1c} values.[34] Recently, a systemic review of antihyperglycemic therapies to prevent the onset of diabetes could not detect a significant effect of metformin.[35] In line with these previous observations, we did not see a difference in the incidence of new-onset diabetes at 4 months.

In our study, measures of left ventricular function were the main end points to investigate the effect of metformin. Left ventricular ejection fraction obtained by MRI is an established efficacy measure because it predicts MACE.[36] Concentration of NT-proBNP as a measure strongly relates with LVEF and clinical outcome.[20,37,38] By using these surrogate measures for clinical outcome we aimed to detect an effect of metformin on outcome. Moreover, we anticipated that the number of MACE would be too small to detect a significant effect of metformin. Indeed, MACE were observed in few patients. Follow-up is ongoing to investigate long-term effects of metformin on clinical outcome.

Conclusions

Among patients without diabetes presenting with STEMI, metformin did not preserve LVEF compared with placebo. A role for metformin in preventing heart failure after myocardial infarction remains unproven.

ARTICLE INFORMATION

Published Online: March 31, 2014. doi:10.1001/jama.2014.3315.

Author Affiliations: University of Groningen, University Medical Center Groningen, Department of Cardiology, Groningen, the Netherlands (Lexis, Lipsic, Wieringa, de Boer, van den Heuvel, van der Werf, Schurer, Pundziute, Tan, Nieuwland, Willemsen, Dorhout, Hillege, van der Harst, van Veldhuisen); University of Groningen, University Medical Center Groningen, Department of Critical Care, Groningen, the Netherlands (van der Horst); University of Groningen, University Medical Center Groningen, Department of Clinical Pharmacy and Pharmacology, Groningen, the Netherlands (Molmans); University of Groningen, University Medical Center Groningen, Department of Endocrinology, Groningen, the Netherlands (van der Horst-Schrivers, Wolffenbuttel); University of Groningen, University Medical Center Groningen, Department of Neuroscience, Groningen, the Netherlands (ter Horst); VU University, VU University Medical Center, Department of Cardiology, Amsterdam, the Netherlands (van Rossum); University of Amsterdam, Academic Medical Center, Department of Cardiology, Amsterdam, the Netherlands (Tijssen); University of Groningen, University Medical Center Groningen, Department of Epidemiology, Groningen, the Netherlands (Hillege); Department of Cardiology, Meander Medical Center, Amersfoort, the Netherlands (de Smet).

Author Contributions: Drs Lexis and van der Harst had full access to all of the data in the study and take responsibility for the integrity of the data and the accuracy of the data analysis. Drs Lexis and van der Horst contributed equally to this article.
Study concept and design: Lexis, van der Horst, de Boer, van den Heuvel, Nieuwland, Dorhout, Molmans, van der Horst-Schrivers, Wolffenbuttel, ter Horst, de Smet, van der Harst, van Veldhuisen.
Acquisition, analysis, or interpretation of data: Lexis, van der Horst, Lipsic, Wieringa, de Boer, van den Heuvel, van der Werf, Schurer, Pundziute, Tan, Willemsen, Dorhout, van der Horst-Schrivers, van Rossum, Tijssen, Hillege, de Smet, van der Harst, van Veldhuisen.
Drafting of the manuscript: Lexis, van der Horst, Lipsic, van der Horst-Schrivers, van der Harst, van Veldhuisen.
Critical revision of the manuscript for important intellectual content: All authors.

Copyright 2014 American Medical Association. All rights reserved.
Downloaded from jamanetwork.com by Rijksuniversiteit Groningen user on 05/06/2026

Statistical analysis: Lexis, van der Horst, Tijssen, Hillege, van der Harst.
Obtained funding: Lexis, van der Horst, de Smet, van der Harst, van Veldhuisen.
Administrative, technical, or material support: Lexis, van der Horst, Lipsic, Wieringa, van den Heuvel, van der Werf, Schurer, Pundziute, Tan, Nieuwland, Willemsen, Dorhout, Molmans, van der Horst-Schrivers, Wolffenbuttel, ter Horst, van Rossum, Hillege, de Smet, van der Harst.
Study supervision: van der Horst, Lipsic, de Boer, van den Heuvel, Nieuwland, Dorhout, van der Horst-Schrivers, Wolffenbuttel, ter Horst, van Rossum, Hillege, van der Harst, van Veldhuisen.

Conflict of Interest Disclosures: All authors have completed and submitted the ICMJE Form for Disclosure of Potential Conflicts of Interest. Dr de Boer reported receiving research grants from Abbott and BG Medicine Inc; serving as a consultant to Abbott, BG Medicine Inc, Novartis, and Medcon; receiving speakers' fees from Abbott, BG Medicine Inc, Novartis, Pfizer, AstraZeneca, Baxter, and Biomerieaux; and holding ownership interest in Pectacea.com and scPharmaceuticals. Dr Wolffenbuttel reported receiving grant support for clinical studies and also consulting fees for serving on advisory boards and as a speaker for Eli Lilly and Company, GlaxoSmithKline, Novo Nordisk, and Pfizer; and receiving consulting fees from Eli Lilly and Company as a member of the 4B study and the DURABLE Trial Data Monitoring Committee. Dr van Veldhuisen reported receiving board membership fees and/or travel expenses from Amgen, Johnson & Johnson, Novartis, Sorbent, Vifor, BG Medicine, and BioControl. No other authors reported disclosures.

Funding/Support: The GIPS-III trial was supported by grant 95103007 from ZonMw, the Netherlands Organization for Health Research and Development, The Hague, the Netherlands.

Role of the Sponsor: The Netherlands Organization for Health Research and Development had no role in the design and conduct of the study; the collection, management, analysis, and interpretation of the data; the preparation, review, or approval of the manuscript; or the decision to submit the manuscript for publication.

GIPS-III Investigators: All in the Netherlands: *University Medical Center Groningen, Groningen:* Iwan C. C. van der Horst (principal investigator), Chris P. H. Lexis, Erik Lipsic, Pim van der Harst, Dirk J. van Veldhuisen, Wouter G. Wieringa, Rudolf A. de Boer, Ad F. M. van den Heuvel, Hindrik W. van der Werf, Remco A. J. Schurer, Gabija Pundziute, Eng S. Tan, Hendrik M. Willemsen, Anouk N. A. van der Horst-Schrivers, Bruce H. R. Wolffenbuttel, Bernard Dorhout, Hans L. Hillege, Wybe Nieuwland, Peter van der Meer, René A. Tio, Jenifer Coster, Yoran M. Hummel, Barbara H. W. Molmans, Gert J. ter Horst, Remco Renken, Anita J. Sibeijn-Kuiper; *Meander Medical Center:* Bart J. G. L. de Smet; *VU University Medical Center, Amsterdam:* Albert C. van Rossum, Robin Nijveldt; *Academic Medical Center, Amsterdam:* Jan G. P. Tijssen. **GIPS-III Steering Committee:** Iwan C. C. van der Horst, Erik Lipsic, Pim van der Harst, Rudolf A. de Boer, Anouk N. A. van der Horst-Schrivers, Bruce H. R. Wolffenbuttel, Dirk J. van Veldhuisen. **Writing Committee:** Chris P. H. Lexis, Iwan C. C. van der Horst, Erik Lipsic, Pim van der Harst, Dirk J. van Veldhuisen. **Data and Safety Monitoring Board:** Jan G. P. Tijssen, Robert J. de Winter, Arne J. Risselada, Richard M. de Jong, Rob K. Gonera. **End Point Adjudication Committee:** Vincent M. Roolvink, André P. van Beek, Fred van den Berg.

REFERENCES

1. Steg PG, James SK, Atar D, et al; Task Force on the management of ST-segment elevation acute myocardial infarction of the European Society of Cardiology (ESC). ESC Guidelines for the management of acute myocardial infarction in patients presenting with ST-segment elevation. *Eur Heart J*. 2012;33(20):2569-2619.

2. O'Gara PT, Kushner FG, Ascheim DD, et al; American College of Emergency Physicians; Society for Cardiovascular Angiography and Interventions. 2013 ACCF/AHA guideline for the management of ST-elevation myocardial infarction: a report of the American College of Cardiology Foundation/American Heart Association Task Force on Practice Guidelines. *J Am Coll Cardiol*. 2013;61(4):e78-e140.

3. Patel MR, Dehmer GJ, Hirshfeld JW, Smith PK, Spertus JA. ACCF/SCAI/STS/AATS/AHA/ASNC 2009 Appropriateness Criteria for Coronary Revascularization: a report of the American College of Cardiology Foundation Appropriateness Criteria Task Force, Society for Cardiovascular Angiography and Interventions, Society of Thoracic Surgeons, American Association for Thoracic Surgery, American Heart Association, and the American Society of Nuclear Cardiology: endorsed by the American Society of Echocardiography, the Heart Failure Society of America, and the Society of Cardiovascular Computed Tomography. *Circulation*. 2009;119(9):1330-1352.

4. Terkelsen CJ, Sørensen JT, Maeng M, et al. System delay and mortality among patients with STEMI treated with primary percutaneous coronary intervention. *JAMA*. 2010;304(7):763-771.

5. Pinto DS, Frederick PD, Chakrabarti AK, et al; National Registry of Myocardial Infarction Investigators. Benefit of transferring ST-segment-elevation myocardial infarction patients for percutaneous coronary intervention compared with administration of onsite fibrinolytic declines as delays increase. *Circulation*. 2011;124(23):2512-2521.

6. Velagaleti RS, Pencina MJ, Murabito JM, et al. Long-term trends in the incidence of heart failure after myocardial infarction. *Circulation*. 2008;118(20):2057-2062.

7. Weir RA, McMurray JJ, Velazquez EJ. Epidemiology of heart failure and left ventricular systolic dysfunction after acute myocardial infarction: prevalence, clinical characteristics, and prognostic importance. *Am J Cardiol*. 2006;97(10A):13F-25F.

8. Steg PG, Dabbous OH, Feldman LJ, et al; Global Registry of Acute Coronary Events Investigators. Determinants and prognostic impact of heart failure complicating acute coronary syndromes: observations from the Global Registry of Acute Coronary Events (GRACE). *Circulation*. 2004;109(4):494-499.

9. McAlister FA, Quan H, Fong A, Jin Y, Cujec B, Johnson D. Effect of invasive coronary revascularization in acute myocardial infarction on subsequent death rate and frequency of chronic heart failure. *Am J Cardiol*. 2008;102(1):1-5.

10. Levy D, Kenchaiah S, Larson MG, et al. Long-term trends in the incidence of and survival with heart failure. *N Engl J Med*. 2002;347(18):1397-1402.

11. Lexis CP, van der Horst IC, Lipsic E, et al; GIPS-III Investigators. Metformin in non-diabetic patients presenting with ST elevation myocardial infarction: rationale and design of the glycometabolic intervention as adjunct to primary percutaneous intervention in ST elevation myocardial infarction (GIPS)-III trial. *Cardiovasc Drugs Ther*. 2012;26(5):417-426.

12. El Messaoudi S, Rongen GA, de Boer RA, Riksen NP. The cardioprotective effects of metformin. *Curr Opin Lipidol*. 2011;22(6):445-453.

13. Yin M, van der Horst IC, van Melle JP, et al. Metformin improves cardiac function in a nondiabetic rat model of post-MI heart failure. *Am J Physiol Heart Circ Physiol*. 2011;301(2):H459-H468.

14. Gundewar S, Calvert JW, Jha S, et al. Activation of AMP-activated protein kinase by metformin improves left ventricular function and survival in heart failure. *Circ Res*. 2009;104(3):403-411.

15. Calvert JW, Gundewar S, Jha S, et al. Acute metformin therapy confers cardioprotection against myocardial infarction via AMPK-eNOS-mediated signaling. *Diabetes*. 2008;57(3):696-705.

16. Mellbin LG, Malmberg K, Norhammar A, Wedel H, Rydén L; DIGAMI 2 Investigators. The impact of glucose lowering treatment on long-term prognosis in patients with type 2 diabetes and myocardial infarction: a report from the DIGAMI 2 trial. *Eur Heart J*. 2008;29(2):166-176.

17. Mellbin LG, Malmberg K, Norhammar A, Wedel H, Rydén L; DIGAMI 2 Investigators. Prognostic implications of glucose-lowering treatment in patients with acute myocardial infarction and diabetes: experiences from an extended follow-up of the Diabetes Mellitus Insulin-Glucose Infusion in Acute Myocardial Infarction (DIGAMI) 2 Study. *Diabetologia*. 2011;54(6):1308-1317.

18. Lexis CP, Wieringa WG, Hiemstra B, et al. Chronic metformin treatment is associated with reduced myocardial infarct size in diabetic patients with ST-segment elevation myocardial infarction. *Cardiovasc Drugs Ther*. 2014;28(2):163-171.

19. Pokorney SD, Rodriguez JF, Ortiz JT, Lee DC, Bonow RO, Wu E. Infarct healing is a dynamic process following acute myocardial infarction. *J Cardiovasc Magn Reson*. 2012;14:62.

20. van Veldhuisen DJ, Linssen GC, Jaarsma T, et al. B-type natriuretic peptide and prognosis in heart failure patients with preserved and reduced ejection fraction. *J Am Coll Cardiol*. 2013;61(14):1498-1506.

21. Doughty RN, Whalley GA, Walsh HA, Gamble GD, López-Sendón J, Sharpe N; CAPRICORN Echo Substudy Investigators. Effects of carvedilol on left ventricular remodeling after acute myocardial infarction: the CAPRICORN Echo Substudy. *Circulation*. 2004;109(2):201-206.

22. Sharpe N, Smith H, Murphy J, Greaves S, Hart H, Gamble G. Early prevention of left ventricular dysfunction after myocardial infarction with angiotensin-converting-enzyme inhibition. *Lancet*. 1991;337(8746):872-876.

23. Hirsch A, Nijveldt R, van der Vleuten PA, et al; HEBE Investigators. Intracoronary infusion of

Copyright 2014 American Medical Association. All rights reserved.

Downloaded from jamanetwork.com by Rijksuniversiteit Groningen user on 05/06/2026

mononuclear cells from bone marrow or peripheral blood compared with standard therapy in patients after acute myocardial infarction treated by primary percutaneous coronary intervention: results of the randomized controlled HEBE trial. *Eur Heart J*. 2011;32(14):1736-1747.

24. Inzucchi SE, Bergenstal RM, Buse JB, et al; American Diabetes Association (ADA); European Association for the Study of Diabetes (EASD). Management of hyperglycemia in type 2 diabetes: a patient-centered approach: position statement of the American Diabetes Association (ADA) and the European Association for the Study of Diabetes (EASD). *Diabetes Care*. 2012;35(6):1364-1379.

25. Tucker GT, Casey C, Phillips PJ, Connor H, Ward JD, Woods HF. Metformin kinetics in healthy subjects and in patients with diabetes mellitus. *Br J Clin Pharmacol*. 1981;12(2):235-246.

26. Gersh BJ, Stone GW, White HD, Holmes DR Jr. Pharmacological facilitation of primary percutaneous coronary intervention for acute myocardial infarction: is the slope of the curve the shape of the future? *JAMA*. 2005;293(8):979-986.

27. UK Prospective Diabetes Study (UKPDS) Group. Effect of intensive blood-glucose control with metformin on complications in overweight patients with type 2 diabetes (UKPDS 34). *Lancet*. 1998;352(9131):854-865.

28. Kao J, Tobis J, McClelland RL, et al; Investigators in the Prevention of Restenosis With Tranilast and Its Outcomes Trial. Relation of metformin treatment to clinical events in diabetic patients undergoing percutaneous intervention. *Am J Cardiol*. 2004;93(11):1347-1350.

29. Roussel R, Travert F, Pasquet B, et al; Reduction of Atherothrombosis for Continued Health (REACH) Registry Investigators. Metformin use and mortality among patients with diabetes and atherothrombosis. *Arch Intern Med*. 2010;170(21):1892-1899.

30. Li J, Xu JP, Zhao XZ, Sun XJ, Xu ZW, Song SJ. Protective effect of metformin on myocardial injury in metabolic syndrome patients following percutaneous coronary intervention. *Cardiology*. 2014;127(2):133-139.

31. Rydén L, Grant PJ, Anker SD, et al; Authors/Task Force Members; ESC Committee for Practice Guidelines (CPG); Document Reviewers. ESC Guidelines on diabetes, pre-diabetes, and cardiovascular diseases developed in collaboration with the EASD: the Task Force on diabetes, pre-diabetes, and cardiovascular diseases of the European Society of Cardiology (ESC) and developed in collaboration with the European Association for the Study of Diabetes (EASD). *Eur Heart J*. 2013;34(39):3035-3087.

32. Renda F, Mura P, Finco G, Ferrazin F, Pani L, Landoni G. Metformin-associated lactic acidosis requiring hospitalization: a national 10 year survey and a systematic literature review. *Eur Rev Med Pharmacol Sci*. 2013;17(suppl 1):45-49.

33. Maioli M, Toso A, Leoncini M, Gallopin M, Musilli N, Bellandi F. Persistent renal damage after contrast-induced acute kidney injury: incidence, evolution, risk factors, and prognosis. *Circulation*. 2012;125(25):3099-3107.

34. Preiss D, Lloyd SM, Ford I, et al. Metformin for non-diabetic patients with coronary heart disease (the CAMERA study): a randomised controlled trial. *Lancet Diabetes Endocrinol*. 2014;2(2):116-124.

35. Merlotti C, Morabito A, Pontiroli AE. Prevention of type 2 diabetes: a systemic review and meta-analysis of different intervention strategies [published online January 29, 2014]. *Diabetes Obes Metab*. doi:10.1111/dom.12270.

36. El Aidi H, Adams A, Moons KG, et al. Cardiac magnetic resonance imaging findings and the risk of cardiovascular events in patients with recent myocardial infarction or suspected or known coronary artery disease—a systematic review of prognostic studies. *J Am Coll Cardiol*. 2014;63(11):1031-1045.

37. Scirica BM, Kadakia MB, de Lemos JA, et al; National Cardiovascular Data Registry. Association between natriuretic peptides and mortality among patients admitted with myocardial infarction: a report from the ACTION Registry(R)-GWTG™. *Clin Chem*. 2013;59(8):1205-1214.

38. Luchner A, Hengstenberg C, Löwel H, et al. NT-ProBNP in outpatients after myocardial infarction: interaction between symptoms and left ventricular function and optimized cut-points. *J Card Fail*. 2005;11(5)(suppl):S21-S27.

Copyright 2014 American Medical Association. All rights reserved.

Downloaded from jamanetwork.com by Rijksuniversiteit Groningen user on 05/06/2026
