# Supplementary material for: Sex differences in cardiac structure and function following ST-segment elevation myocardial infarction
Source: Sci Rep. 2026 May 19;16:22756. doi: 10.1038/s41598-026-52993-8 (PMC13385960; doi:10.1038/s41598-026-52993-8)
Supplement: Supplementary file 3 — Supplementary Material 3 [file 41598_2026_52993_MOESM3_ESM.pdf]

Cardiovasc Drugs Ther (2012) 26:417–426
DOI 10.1007/s10557-012-6413-1

Metformin in non-Diabetic Patients Presenting with ST Elevation Myocardial Infarction: Rationale and Design of the Glycometabolic Intervention as Adjunct to Primary Percutaneous Intervention in ST Elevation Myocardial Infarction (GIPS)-III Trial

Chris P. H. Lexis · Iwan C. C. van der Horst · Erik Lipsic · Pim van der Harst · Anouk N. A. van der Horst-Schrivers · Bruce H. R. Wolffenbuttel · Rudolf A. de Boer · Albert C. van Rossum · Dirk J. van Veldhuisen · Bart J. G. L. de Smet · for the GIPS-III Investigators

Published online: 12 September 2012
© The Author(s) 2012. This article is published with open access at Springerlink.com

Abstract
Background Left ventricular dysfunction and the development of heart failure is a frequent and serious complication of myocardial infarction. Recent animal experimental studies suggested that metformin treatment reduces myocardial injury and preserves cardiac function in non-diabetic rats after experimental myocardial infarction. We will study the efficacy of metformin with the aim to preserve left ventricular ejection fraction in non-diabetic patients presenting with ST elevation myocardial infarction (STEMI).
Methods The Glycometabolic Intervention as adjunct to Primary percutaneous intervention in ST elevation myocardial infarction (GIPS)-III trial is a prospective, single center, double blind, randomized, placebo-controlled trial. Three-hundred-and-fifty patients, without diabetes, requiring primary percutaneous coronary intervention (PCI) for STEMI will be randomized to metformin 500 mg twice daily or placebo treatment and will undergo magnetic resonance imaging (MRI) after 4 months. Major exclusion criteria were prior myocardial infarction and severe renal dysfunction. The primary efficacy parameter is left ventricular ejection fraction 4 months after randomization. Secondary and tertiary efficacy parameters include major adverse cardiac events, new onset diabetes and glycometabolic parameters, and echocardiographic diastolic function. Safety parameters include renal function deterioration and lactic acidosis.
Conclusions The GIPS-III trial will evaluate the efficacy of metformin treatment to preserve left ventricular ejection fraction in STEMI patients without diabetes.

Key words ST-elevation myocardial infarction · Metformin · Left ventricular ejection fraction · Heart failure · Cardiac remodeling

Chris P. H. Lexis and Iwan C. C. van der Horst contributed equally

C. P. H. Lexis (✉) · I. C. C. van der Horst · E. Lipsic · P. van der Harst · R. A. de Boer · D. J. van Veldhuisen · B. J. G. L. de Smet
Department of Cardiology, University Medical Center Groningen, University of Groningen,
Hanzeplein 1, 9700 RB, PO Box 30.001,
Groningen, the Netherlands
e-mail: c.p.h.lexis@umcg.nl

I. C. C. van der Horst
Department of Critical Care,
University Medical Center Groningen, University of Groningen,
Groningen, The Netherlands

A. N. A. van der Horst-Schrivers · B. H. R. Wolffenbuttel
Department of Endocrinology and Metabolism,
University Medical Center Groningen, University of Groningen,
Groningen, the Netherlands

A. C. van Rossum
Department of Cardiology, VU Medical Center,
Amsterdam, The Netherlands

Background

Primary percutaneous coronary intervention (PCI) reduces early mortality and improves late clinical outcome in patients with acute myocardial infarction (MI). Large MI size and adverse left ventricular remodeling may cause post infarct deterioration of left ventricular function and development of overt heart failure.

Metformin, a biguanide oral antihyperglycemic agent used widely for the treatment of patients with type 2 diabetes mellitus, enhances glucose control through increased glucose utilization and decreased endogenous glucose release [1, 2]. Several studies in patients with diabetes demonstrated that metformin is associated with improved outcome and considered to be safe (Table 1) [3–9]. Furthermore, in patients at high risk of developing diabetes, metformin reduced the incidence of diabetes [10, 11].

Several preclinical studies in non-diabetic animals reported that metformin may confer cardioprotection by limiting MI size and preventing adverse remodeling. Recently, our group demonstrated that metformin reduces infarct size by 22 % in an experimental non-diabetic rat model of MI, resulting in a relative improvement in left ventricular ejection fraction (LVEF) of 52 % compared to placebo (Fig. 1) [12]. These effects were independent from glycemic control, as these were non-diabetic normoglycemic rats [12]. Other groups confirmed these results in murine and canine models, demonstrating metformin treatment compared to placebo reduced MI size between 22 % to 58 % [13–17], and resulted in a relative improvement in LVEF between 31 and 52 % [13, 14].

Current medical strategies are predominantly aimed at establishing reperfusion and secondary prevention including prevention of thrombo-embolism and inhibition of the renin-angiotensin system. Reducing infarct size and positive cardiac remodeling by metformin therapy provides a potential novel strategy to preserve functional myocardium and thereby improve prognosis.

Mechanism of action and potential benefit

Effects of metformin include decreased hyperglycemia, hypoinsulinemia, higher peripheral muscle glucose uptake, decreased hepatic glyconeogenesis, reduced hypercoagulability, improvement of the lipid profile, nitric oxide mediated vasodilatation, and additional cardioprotective effects [1]. Reported cardioprotective effects of metformin include attenuation of myocardial infarct size and improved left ventricular function. The exact mechanisms of action that explain these numerous effects of metformin remain to be elucidated. Especially our understanding of the cardioprotective effects of metformin, beyond glucose lowering, is incomplete [18]. Several ancillary mechanisms have been proposed to explain

Table 1 Effects of metformin on prognosis. RCT: randomized controlled trial; BMI: Body Mass Index; STEMI: ST-elevation myocardial infarction; HR: hazard ratio; OR: odds ratio

Ref.	Subjects	Study design	Number of subjects	Median follow-up	Effect of metformin on blood glucose levels	Effect of metformin on endpoints
3	Obese (BMI >27 kg/m^2) patients with diabetes	Post-hoc analysis of RCT	753	10.7 years	Comparable to other strategies	Improved survival ($p=0.021$)
4	STEMI patients with diabetes	Post-hoc analysis of RCT	1,145	4.1 years	Comparable to other strategies	Improved survival (HR death: 0.65, 0.47–0.90, $p=0.01$)
5	Patients with diabetes undergoing coronary intervention	Post-hoc analysis of RCT	2,772	9 months	Higher blood glucose levels compared to other strategies	Improved survival (OR death: 0.41, 0.21–0.79, $p=0.008$)<br>Lower rates of MI (OR MI: 0.41, 0.20–0.84, $p=0.016$)
6	Patients with diabetes and atherothrombosis	Observational	19,691	2 years	Higher blood glucose levels compared to other strategies	Improved survival (HR death: 0.76, 0.65–0.89, $p<0.001$)
7	Elderly patients with diabetes and heart failure	Retrospective	16,417	1 year	No data on glycemic control	Improved survival (HR death: 0.86, 0.78–0.97)
8	Patients with diabetes and heart failure	Observational	6,185	2 years	No data on glycemic control	Improved survival (HR death: 0.76, 0.63–0.92, $p<0.001$)
9	Patients with type 2 diabetes	Meta-analysis	96,295	1.3 years	No data on glycemic control	No increased risk for lactic acidosis<br>No differences in lactate levels

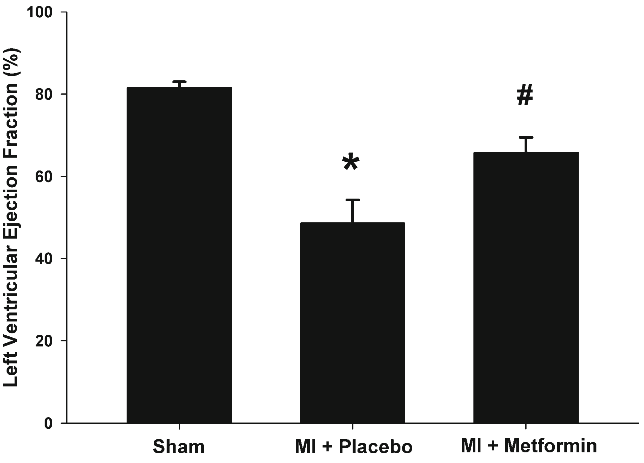

Fig. 1 metformin resulted in a relative improvement in left ventricular ejection fraction of 52 % compared to placebo. MI: myocardial infarction; *$P<0.05$ vs. sham group; # $P<0.05$ vs. placebo group. Adapted with permission from: Fig. 2 from Meimei Yin, Iwan CC van der Horst, Joost P van Melle, Cheng Qian, Wiek H van Gilst, Herman HW Silljé, and Rudolf A de Boer. Metformin improves cardiac function in a nondiabetic rat model of post-MI heart failure. Am J Physiol Heart Circ Physiol August 2011 301:(2) H459–H468

metformin induced cardioprotection, these are displayed in detail in Fig. 2 [12–33].

Collectively, the metformin induced changes in myocardial gene and energy program, especially the activation of AMPK, are associated with decreased infarct size, prevention of adverse remodeling, and result ultimately in improved cardiac function.

Study design

The GIPS-III trial is a single center, prospective, double-blind, randomized, placebo-controlled trial, designed to evaluate the efficacy of a 4 month metformin treatment on preservation of LVEF in non-diabetic STEMI patients requiring primary PCI treatment. A total of 350 non-diabetic STEMI patients will be included in the GIPS-III trial.

Eligibility

In- and exclusion criteria are listed in Table 2. In brief, subjects presenting with an acute STEMI treated with primary PCI, including the implantation of at least 1 stent with a diameter of at least 3.0 mm are considered for this trial. Verbal followed by written informed consent will be required from each patient.

Treatment

All patients will receive standard medical treatment for a STEMI according to European practice guidelines [34].

The flow chart of the GIPS-III trial is shown in Fig. 3. During the primary PCI procedure witnessed verbal informed consent will be obtained by the interventional cardiologist and additional blood samples will be drawn for storage. As soon as possible, but no more than 3 h after successful PCI, patients will be randomly assigned to a 4 month treatment with white film-coated tablets containing metformin hydrochloride 500 mg or visually matching placebo, administered twice daily. Secondary prevention will according to ESC guidelines include aspirin, thienopyridines, statins, angiotensin converting enzyme (ACE) inhibitors, and beta-receptor blockers, when indicated and tolerated [34, 35].

Subjects are scheduled for return visits at 2 weeks, 2 months, 4 months, and 12 months after hospital discharge. During every visit examination, assessment of clinical events and 12-lead electrocardiography are performed. During the 4 month visit the amount of study drug received, dispensed, and consumed will be recorded.

Study drug treatment will be discontinued in the following situations: 1) when subject withdraw consent, 2) in case of pregnancy, 3) when subjects develop severe renal dysfunction (defined as creatinin >177 μmol/L, or an estimated GFR <30 ml/min*1.73 m^2), or 4) when subjects develop a condition which, in the investigator's judgment, precludes further therapy. Discontinuation will have no consequence for the regular patient care. Since metformin therapy used as a single antihyperglycemic drug is not associated with hypoglycemia and due to the blinded nature of this trial, there will be no dose modifications of the study medication.

Study efficacy parameters

The primary efficacy parameter of the GIPS-III trial is LVEF measured by cardiac MRI 4 months after randomization, based on an intention-to-treat analysis. LVEF, is an important predictor of prognosis after MI [36, 37].

A per-protocol analysis, excluding patients diagnosed with new onset diabetes and treated with oral antihyperglyceemic agents or insulin prior to cardiac MRI, will be performed as a secondary efficacy parameter. Other secondary efficacy parameters include major cardiac adverse events (MACE; death, recurrent MI, target lesion revascularization), stroke, non-elective hospitalizations for chest pain or heart failure, all recurrent coronary interventions, and internal cardiac defibrillator implantations. Mortality will be divided into cardiac and non-cardiac. Cardiac death will be divided into three categories: heart failure, sudden death and other. A cardiologist will confirm deaths from cardiovascular causes by examining medical records obtained from hospitals and attending physicians or from the attending general practitioner if the patient died at home. Further, echocardiographic parameters of diastolic function, incidence of new onset diabetes, additional parameters

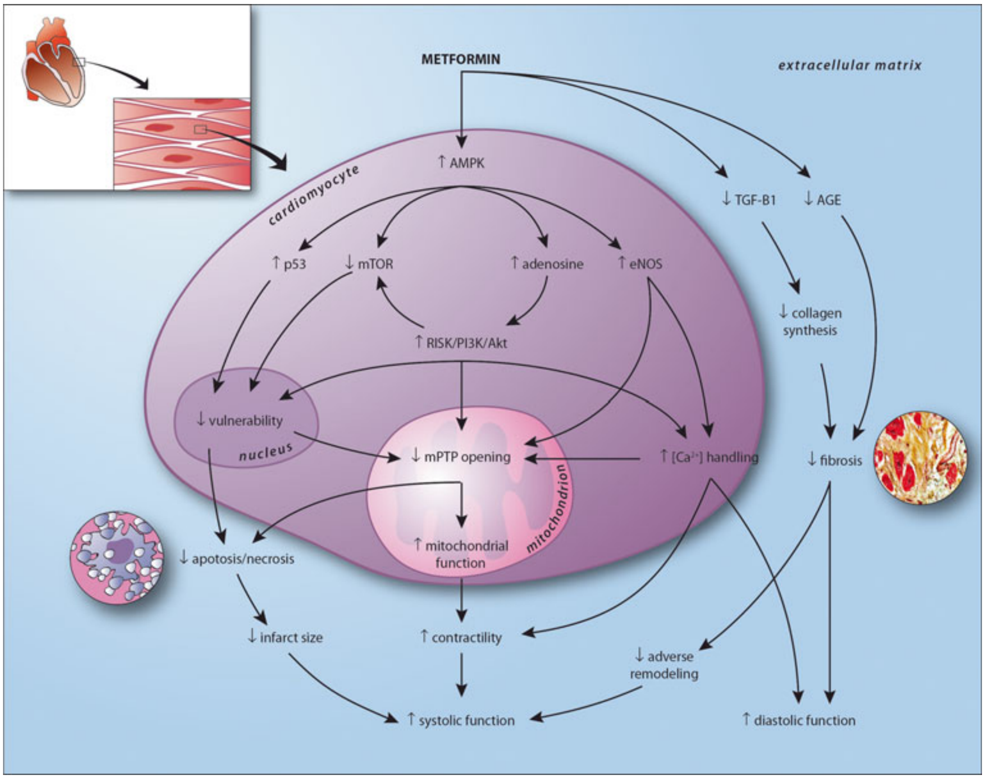

Fig. 2 visualization of the proposed cardioprotective mechanism of action of metformin in the human heart after myocardial infarction, resulting in improved systolic and diastolic function. In experimental models metformin has been consistently associated with enhanced phosphorylation of AMP activated protein kinase (AMPK) [12–24]. In the myocardium, characterized by high energy demands and low energy reserves, AMPK plays a pivotal role in maintaining metabolic homeostasis [18–21]. Metformin-induced AMPK phosphorylation may be mediated by inhibition of complex 1 of the respiratory chain, by upstream activation of the tumor suppressor gene liver kinase B1 (LKB1), or by decreased AMP–deaminase activity [21–24]. AMPK phosphorylation leads to activation of the Reperfusion Injury Salvage Kinase (RISK) pathway including phospatidylinositol-3-kinase (PI3K) and Akt pathways [17, 26], upregulation of the tumor suppressor gene p53 [27], inhibition of mammalian target of rapamycin (mTOR) [19], and upregulation of endothelial nitric oxide synthase (eNOS) [1, 13]. Activation of the RISK pathway and eNOS improves mitochondrial function and inhibits opening of the mitochondrial permeability transition pore (mPTP) [26]. The mPTP is a major mediator of myocardial reperfusion injury. Opening of the mPTP results in ATP depletion and cell death [25, 26]. Further, prevention of mPTP opening stimulates mitochondrial respiration, improving ATP availability and cellular function [25]. Upregulated p53 and inhibited mTOR, partly RISK pathway mediated, are associated with decreased cellular vulnerability by preventing post-mitotic cell death and improved resilience to ischemia related injury [19, 27]. Metformin mediated eNOS production, next to increasing nitric oxide production, enhances sodium pump activity causing decreased intracellular calcium levels [1]. In infarcted tissue, this may attenuate microvascular obstruction and thereby prevent mPTP mediated cell death [28]. In functional myocardium, optimized calcium handling results in improved contractility and relaxation [1]. Further, independent of AMPK, metformin inhibits transforming growth factor (TGF)-β1 myocardial expression, decreasing collagen synthesis and preventing fibrosis [29]. Metformin may also attenuate cardiac fibrosis by directly inhibiting advanced glycation endproduct (AGE) formation [30]. Also, metformin is associated with a decrease in dipeptidyl peptidase-4 activity and an increase in circulating levels of glucagon-like peptide 1 [31]. In a porcine model of ischemia and reperfusion injury, stimulation with a analogue (exenatide) resulted in a reduction of infarct size [32]. Another target of metformin may be the increase of glucose utilisation of the heart. The adult heart mainly relies on fatty acids utilisation, and switches back to glucose when damaged. However, metabolic flexibility of the failing heart is limited, and facilitation of glucose utilisation by metformin via increase of glucose transporters (GLUT-1 and GLUT-4) may explain its salutary effects on the cardiac function [12, 33]

measured by MRI, skin autofluorescence, electrocardiographic parameters, and blood sample analyses, will be used as tertiary efficacy endpoints.

Magnetic resonance imaging

Cardiovascular MRI is considered the most accurate measure to date for evaluation of LVEF, the extent of myocardial infarct size, and several other functional parameters [36]. The diagnostic accuracy of MRI for evaluation of LVEF allows a sample size reduction compared to other imaging modalities. Patients are studied with a 3.0 Tesla clinical scanner (3 T Achieva, Philips, Best, The Netherlands) at the NeuroImaging Center (NIC, University of Groningen, Groningen, The Netherlands) (Appendix C) using a phased array cardiac receiver coil. Electrocardiogram-gated cine steady-state, free precession magnetic resonance images acquired during repeated breath holds in the standard long-

Table 2 In- and exclusion criteria for the GIPS-III trial. MI: myocardial infarction; ECG: electrocardiogram; PCI: percutaneous coronary intervention; MRI: magnetic resonance imaging

Inclusion criteria	Exclusion criteria
• The diagnosis acute MI defined by chest pain suggestive for myocardial ischemia for at least 30 min, the time from onset of the symptoms less than 12 h before hospital admission, and an ECG recording with ST- segment elevation of more than 0.1 mV in 2 or more leads	• Prior MI<br>• Diabetes<br>• Creatinin >177 μmol/L measured pre-PCI<br>• Need for coronary artery bypass grafting<br>• Rescue PCI after thrombolytic therapy
• Successful primary PCI <12 h from onset of symptoms	• When subjects develop a condition which, in the investigator's judgment, precludes study therapy
• Verbal followed by written informed consent	• Inability to provide informed consent
• At least one stent sized ≥3.0 mm	• Younger than 18 years
• Eligible for cardiac MRI-scan:	• Contra-indication to metformin
- Body Mass Index <40 kg/m^2<br>- no ferromagnetic metal objects in the body	• an estimated life-expectancy of less than 6 months
- no claustrophobia	

axis views (4-, 3-, and 2-chamber view) and contiguous short-axis slices covering the entire left ventricle are used to assess global and regional ventricular function and to calculate LVEF (primary endpoint). Using identical slice locations, late contrast-enhanced (LCE) images are acquired 10 min after intravenous administration of a gadolinium-based contrast agent (Dotarem, Gorinchem, the Netherlands; 0.2 mmol/kg) with an inversion-recovery, gradient-echo pulse sequence to identify the location and extent of MI. The inversion time will be set to null the signal of viable myocardium for every

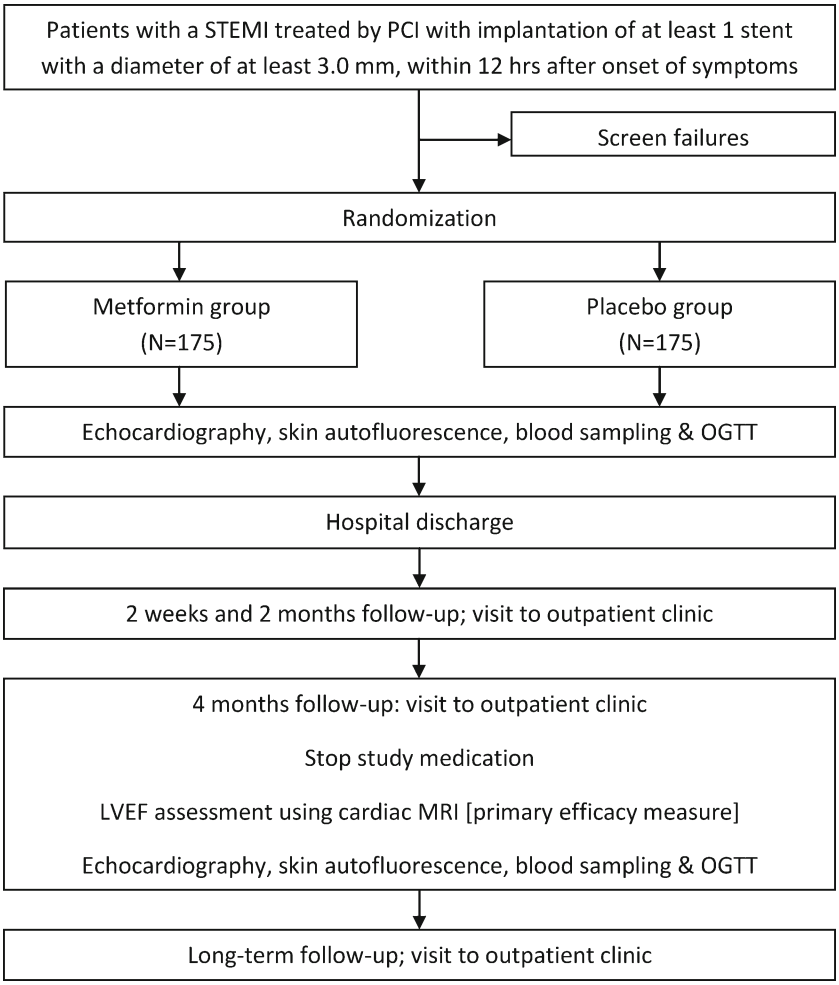

Fig. 3 Flow chart of the GIPS-III trial. STEMI, ST-elevation myocardial infarction; PCI, percutaneous coronary intervention; LVEF, left ventricular ejection fraction; MRI magnetic resonance imaging; OGTT oral glucose tolerance testing

individual patient. All MRI data are sent to independent cardiologists, blinded for randomization status, for quality control and blinded analysis (Appendix D).

The MRI data are analyzed using a dedicated software package. On the stack of short-axis cines, the endocardial and epicardial borders are outlined in end systolic and end diastolic images. Left ventricular end diastolic volume (LVEDV) and left ventricular end systolic volume (LVESV) are calculated using the summation of slice method multiplied by slice distance. LVEF is calculated as LVEF=100 %×(LVEDV-LVESV)/LVEDV. Summation of the volumes per slice of areas of hyperenhancement is outlined, allowing to calculate total infarct size.

Echocardiography

Two-dimensional echocardiography with a phased array electronic ultrasound will be performed 0–2 days after randomization and 4 months after randomization. Tissue Doppler (TD) imaging of the early mitral valve flow velocity/early TD lengthening velocity (*E/E'*), the ratio of the early (E) to late (A) mitral valve flow velocity, the deceleration time, the left atrial volume index (LAVI), and the difference between the duration of reverse pulmonary vein atrial systole flow (Ard) and mitral valve atrial wave flow (Ad) will be used to determine and classify diastolic function.

Skin autofluorescence

Tissue AGE accumulation will be assessed using a validated skin autofluorescence (AF) reader (advanced glycation end-products reader; patent PCT/NL99/00607; DiagnOptics BV, Groningen, The Netherlands) [38]. In short, a skin surface of approximately 2 cm^2 is illuminated by the AGE-reader with an peak excitation of ~370 nm. The reflected light from the skin is measured with a spectrometer in the 420–600 nm range, using 200 μm glass fibers. The value of skin AF is calculated as the ratio of the light intensity in the 420–600 nm wavelength range and the light intensity in the 300–420 nm wavelength range. Skin AF will be measured during hospitalization, and 4 months after randomization.

Electrocardiography

A standard 12-lead electrocardiogram is acquired at the time of presentation, after the PCI procedure, before hospital discharge, and at each outpatient clinic visit. Mean time interval between pre and post intervention will be registered. Pre-intervention ECG will be analyzed on the presence of ST-deviation. The post-intervention ECGs will be used to score persistent ST-deviation and ST-segment resolution, and the incidence and location of new Q-waves.

Laboratory analysis

During hospitalization, blood will be sampled at baseline and at 3, 6, 9, 12, and 24 h after PCI to monitor values of cardiac enzymes and high sensitive troponin [39]. Less frequently during hospitalization and at every visit to the outpatient clinic hemoglobin, platelets, glucose, creatinin and liver enzymes, total cholesterol, high-density and low-density lipoprotein, and N-terminal pro B-type natriuretic peptide will be determined.

Furthermore, during PCI, 24 h after PCI, and at every visit to the outpatient clinic, blood samples for additional analyses will be collected [40]. These analyses will include, but are not limited to, glycometabolic determinants, biomarkers, and other markers of disease severity or relevant to the disease [41, 42].

Diabetes and prediabetes

For assessment of diabetes and prediabetes, an oral glucose tolerance test (OGTT) will be performed during initial hospitalization and after study medication is stopped according to protocol and after primary endpoint analysis, next to the level of glycated hemoglobin (HbA1c) [40]. Diabetes and prediabetes will be diagnosed according to current guidelines [43]. Whenever new onset diabetes is diagnosed, patients will be treated by an endocrinologist according to current guidelines, additional to study treatment. Metformin can be started on top of study medication in a dose of 500 mg three times per day, to prevent exceeding maximal metformin dose. Patients who next to standard care and life style interventions need oral antihyperglycemic agents or insulin for glucose control prior to primary endpoint analysis will be excluded from the per protocol analysis.

Statistical considerations

Sample size

The sample size is calculated for the difference in the primary efficacy parameter (LVEF measured by MRI at 4 months) between the intervention group and the placebo group. With 80 % power to detect a 3 % difference in LVEF between active treatment and control (assuming a 2-sided α of 0.05 and an SD of 9 % for the change in LVEF) 141 patients are needed in each study group. A 3 % difference in LVEF is considered to be a clinically relevant outcome [37]. Based on local experience from previous studies, we assume that MRI analysis will be unavailable in up to 24 % of patients (due to study withdrawal, development of contra-indications e.g. ICD, claustrophobia, etc.) [44, 45]. To maintain 80 % power, an increase to a total of 350 patients is

required. However, if actual study completion rates differ from predicted rates, recruitment will be extended in order to achieve 282 patients with primary endpoint analysis. The maximal number of patients to which the inclusion can be extended in this trial will be limited to 380.

Statistical analyses of primary and secondary efficacy parameters

The primary efficacy parameter of the study is measured 4 months after randomization. For the analysis of binary endpoints, treatment comparisons will be performed using Fisher exact probability test or Chi-square analysis. For continuous outcomes, independent samples *t* test or a Mann–Whitney *U* test will be used, as appropriate. For clinical outcomes such as the incidence of major adverse cardiac events, Cox regression will be used to evaluate the association between the intervention and the endpoints. Kaplan-Meier curves displaying the pattern of events over the 4-month and long-term follow-up period will be drawn.

Study organization and monitoring

The GIPS-III trial is performed by the GIPS-III investigators (Appendix A), supervised by a steering committee (Appendix B). The steering committee is responsible for design and conduct of the study. Periodic assessments of safety are being performed by an independent data and safety monitoring board (DSMB) (Appendix E). Study endpoints will be assessed by an independent endpoint adjudication committee (EAC) (Appendix F). Data monitoring and data management will be performed by the independent Trial Coordination Center (Appendix G). For valorization purposes a users' committee will be installed (Appendix H). The trial registration number is NCT01217307 (www.clinicaltrials.gov).

Discussion

The GIPS-III trial will be the first randomized, double-blind, placebo-controlled trial to study the efficacy of metformin on preservation of LVEF in non-diabetic STEMI patients. This trial will provide valuable information on whether metformin can preserve LVEF and reduce myocardial infarct size after STEMI and might extend its clinical efficacy beyond patients with diabetes. LVEF was chosen as the primary efficacy parameter as this provides an important reflection of the functional consequences of post infarction cardiac remodeling and is probably more important than anatomical area at risk.

A unique aspect of the GIPS-III trial is that we evaluate non-glycemic effects of metformin in a non-diabetic population. In the current trial we excluded patients with a history of diabetes. Diabetes diagnosed after randomization will be regarded as "new onset diabetes" and will be treated by an endocrinologist which could include metformin treatment in addition to study drug treatment. According to the intention-to-treat principle, these patients will be included in the primary efficacy parameter analysis. For the secondary per protocol analysis these patients will be excluded.

We excluded patients with documented myocardial infarction from the GIPS-III trial to avoid inclusion of subjects with reduced LVEF at baseline, which might complicate the interpretation of our data. We also included only subjects with a STEMI based on a vessel requiring a stent diameter of at least 3 mm as an indicator of a relatively large area at risk which might potentially result in a clearly reduced LVEF. Although the exact mechanism of metformin remains to be elucidated, we start study treatment immediate (within 3 h) after PCI to have the largest possible window of opportunity. Our primary efficacy parameter will be evaluated 4 months after primary PCI. After 4 months would healing should be completed and partial or complete remodeling should have occurred [46].

Several prospective trials in patients with diabetes have reported a favorable outcome associated with metformin. Several retrospective analyses have demonstrated additional effects on cardiovascular endpoints. No prospective trial has yet shown the effects of metformin on myocardial infarct size and cardiac function. The effects and pathways allegedly responsible for the metformin-induced cardioprotective effects have not yet been studied in the human setting. Moreover, the exact contribution and efficacy of the supposed metformin mediated mechanisms to improved systolic and diastolic myocardial function is unclear. However, retrospective data consistently showed that metformin therapy was associated with improved outcome in diabetic patients (Table 1). In non-diabetic preclinical studies a consistent reduction in myocardial infarct size and improvement in left ventricular function has been reported [12–17]. Therefore, the GIPS-III trial may be regarded as a proof-of-principle trial focused on the cardioprotective effects of metformin. Collectively, we hypothesize that the metformin induced changes in myocardial gene and energy program, especially the activation of AMPK, will be associated with decreased infarct size, prevention of adverse remodeling, and may ultimately result in improved systolic function (Fig. 2). Diastolic function might also be improved by attenuating fibrosis and improving myocardial relaxation (Fig. 2). Extensive secondary analyses will allow to study the mechanisms involved with metformin use in a non diabetic population.

Current status

The GIPS-III trial has been approved by the local institutional review board, national regulatory agencies, and is

being carried out according the Declaration of Helsinki (Seoul 2008). GIPS-III has enrolled its first patient in January 2011. As of August 1st, 2012, 266 patients have been randomized. Completion of the inclusion is anticipated in January 2013. Primary endpoint analysis of the final randomized patient is expected in April 2013.

Conclusion

The GIPS-III trial is a single center, prospective, double-blind, randomized, placebo-controlled trial to determine whether a 4 month metformin treatment can improve LVEF in 350 non-diabetic patients presenting with STEMI requiring primary PCI treatment.

Acknowlegdments We acknowledge D. Buiter, medical artist, for his help in creating the figures.

Disclosures This study is supported by a grant of the Netherlands Organization for Health Research and Development (ZonMW; project no. 40-41200-98-9139). The study sponsor has no role in the design and conduct of the study, study analyses, and drafting and editing of the manuscript, and its final contents. The authors declare that they have no conflict of interest.

Open Access This article is distributed under the terms of the Creative Commons Attribution License which permits any use, distribution, and reproduction in any medium, provided the original author(s) and the source are credited.

Appendix A: GIPS-III investigators

CPH Lexis, MD, ICC van der Horst, MD, PhD, (principal investigator), WG Wieringa, MD, E Lipsic, P van der Harst, MD, PhD, MD, PhD, DJ van Veldhuisen, MD, PhD, BJGL de Smet, MD, PhD, HW van der Werf, MD, RAJ Schurer, MD, GP Pundziute, MD, PhD, AFM van den Heuvel, MD, PhD, ES Tan, MD, PhD, HZR Gerds, MANP, RN, RA de Boer, MD, PhD, MH Willemsen, MD, W Nieuwland, MD, J Coster, MD, PhD, RA Tio MD, PhD, P van der Meer, MD, PhD, B Dorhout, PhD, GJ ter Horst, PhD, ANA van der Horst-Schrivers, MD, PhD, BHR Wolffenbuttel, MD, PhD, *University of Groningen, University Medical Center Groningen, Groningen*.

Appendix B: steering committee

ICC van der Horst, MD, PhD, DJ van Veldhuisen, MD, PhD, BJGL de Smet, MD, PhD, P van der Harst, MD, PhD, ANA van der Horst-Schrivers, MD, PhD, RA de Boer, MD, PhD, BHR Wolffenbuttel, MD, PhD, *University of Groningen, University Medical Center Groningen, Groningen*.

Appendix C: NeuroImaging Center

GJ ter Horst, PhD, AJ Sibeijn-Kuiper, *University of Groningen, Groningen.*

Appendix D: MRI analysis

R Nijveldt, MD, PhD, AC van Rossum, MD, PhD, *VU University Medical Center, Amsterdam*.

Appendix E: data and safety monitoring board

JGP Tijssen, PhD, (Chair); RJ de Winter, MD, PhD, *Academic Medical Center, Amsterdam*; RM de Jong, MD, PhD, AJ Risselada, MD, RK Gonera, MD, *Wilhelmina Ziekenhuis Assen, Assen*.

Appendix F: endpoint adjudication committee

F van den Berg, MD, PhD, (Chair), AP van Beek, MD, PhD, *University of Groningen, University Medical Center Groningen, Groningen*; V Roolvink, MD, Isala Klinieken locatie Weezenlanden, afd. Cardiologie, Zwolle.

Appendix G: data monitoring and data management

Trial Coordination Center, *University Medical Center Groningen, Groningen*.

Appendix H: users' committee

ICC van der Horst, MD, PhD (Chair), P van der Harst, MD, PhD (Co-Chair), JW Alffenaar, PhD *University of Groningen, University Medical Center Groningen*, *Groningen*, J Blom (Secretary), *ZonMW, Den Haag*, BJGL de Smet, MD PhD, *Meander Medisch Centrum Amersfoort, Amersfoort,* TDJ Smilde MD, PhD, *Scheperziekenhuis Emmen, Emmen*, K Hoogenberg, MD PhD, *Martini Ziekenhuis, Groningen*, J Sikkema, *Stichting Business Generator Groningen, Transfer Liaison Group, Groningen*.

References

1. Kirpichnikov D, McFarlane SI, Sowers JR. Metformin: an update. Ann Intern Med. 2002;137:25–33.
2. Ryden L, Standl E, Bartnik M, et al. Guidelines on diabetes, pre-diabetes, and cardiovascular diseases: executive summary. The Task Force on Diabetes and Cardiovascular Diseases of the European

Society of Cardiology (ESC) and of the European Association for the Study of Diabetes (EASD). Eur Heart J. 2007;28:88–136.
3. UK Prospective Diabetes Study (UKPDS) Group. Effect of intensive blood-glucose control with metformin on complications in overweight patients with type 2 diabetes (UKPDS 34). Lancet. 1998;352:854–65.
4. Mellbin LG, Malmberg K, Norhammar A, Wedel H, Rydén L, et al. Prognostic implications of glucose-lowering treatment in patients with acute myocardial infarction and diabetes: experiences from an extended follow-up of the Diabetes Mellitus Insulin-Glucose Infusion in Acute Myocardial Infarction (DIGAMI) 2 Study. Diabetologia. 2011;54:1308–17.
5. Kao J, Tobis J, McClelland RL, et al. Relation of metformin treatment to clinical events in diabetic patient undergoing percutaneous intervention. Am J Cardiol. 2004;93:1347–50, A5.
6. Roussel R, Travert F, Pasquet B, et al. Metformin use and mortality among patients with diabetes and atherothrombosis. Arch Intern Med. 2010;170:1892–9.
7. Masoudi FA, Inzucchi SE, Wang Y, Havranek EP, Foody JM, Krumholz HM. Thiazolidinediones, metformin and outcomes in older patients with diabetes and heart failure. Circulation. 2005;111:539–90.
8. Aguilar D, Chan W, Bozkurt B, Ramasubbu K, Deswal A. Metformin use and mortality in ambulatory patients with diabetes and heart failure. Circ Heart Fail. 2011;4:53–8.
9. Salpeter SR, Greyber E, Pasternak GA, Salpeter EE. Risk of fatal and nonfatal lactic acidosis with metformin use in type 2 diabetes mellitus. Cochrane Database Syst Rev. 2010;CD002967.
10. Salpeter SR, Buckley NS, Kahn JA, Salpeter EE. Meta-analysis: metformin treatment in persons at risk for diabetes mellitus. Am J Med. 2008;121:149–57.
11. Knowler WC, Barret-Conner E, Fowler SE, et al. Reduction in the incidence of type 2 diabetes with lifestyle intervention or metformin. N Engl J Med. 2002;346:393–403.
12. Yin M, van der Horst IC, van Melle JP, et al. Metformin improves cardiac function in a nondiabetic rat model of post-MI heart failure. Am J Physiol Heart Circ Physiol. 2011;301:H459–68.
13. Calvert JW, Gundewar S, Jha S, et al. Acute metformin therapy confers cardioprotection against myocardial infarction via AMPK-eNOS-mediated signaling. Diabetes. 2008;57:696–705.
14. Gundewar S, Calvert JW, Jha S, et al. Activation of AMP-activated protein kinase by metformin improves left ventricular function and survival in heart failure. Circ Res. 2009;104:403–11.
15. Solskov L, Lofgren B, Kristiansen SB, et al. Metformin induces cardioprotection against ischaemia/reperfusion injury in the rat heart 24 hours after administration. Basic Clin Pharmacol Toxicol. 2008;103:82–7.
16. Zhou G, Myers R, Li Y, et al. Role AMP-activated protein kinase in mechanism of metformin action. J Clin Invest. 2001;108:1167–74.
17. Paiva M, Riksen NP, Davidson SM, et al. Metformin prevents myocardial reperfusion injury by activating the adenosine receptor. J Cardiovasc Pharmacol. 2009;53:373–8.
18. El Messaoudi S, Rongen GA, de Boer RA, Riksen NP. The cardioprotective effects of metformin. Curr Opin Lipidol. 2011;22:445–53.
19. Jalving M, Gietema JA, Lefrandt JD, et al. Metformin: taking away the candy for cancer? Eur J Cancer. 2010;46:2369–80.
20. Ardehali H, Sabbah HN, Burke MA, et al. Targeting myocardial substrate metabolism in heart failure: potential for new strategies. Eur J Heart Fail. 2012;14:120–9.
21. Hardie DG, Carling D, Gamblin SJ. AMP-activated protein kinase: also regulated by ADP? Trends Biochem Sci. 2011;36:470–7.
22. Owen MR, Doran E, Halestrap AP. Evidence that metformin exerts its anti-diabetic effects through inhibition of complex 1 of the respiratory chain. Biochem J. 2000;348:607–14.
23. Lizcano JM, Goransson O, Toth R, et al. LKB1 is a master kinase that activates 13 kinases of the AMPK subfamily, including MARK/PAR-1. EMBO J. 2004;23:833–43.
24. Ouyang J, Parakhia RA, Ochs RS. Metformin activates AMP kinase through inhibition of AMP deaminase. J Biol Chem. 2001;286:1–11.
25. Hausenloy DJ, Ong SB, Yellon DM. The mitochondrial permeability transition pore as a target for preconditioning and postconditioning. Basic Res Cardiol. 2009;104:189–202.
26. Bhamra GS, Hausenloy DJ, Davidson SM, et al. Metformin protects the ischemic heart by the Akt-mediated inhibition of mitochondrial permeability transition pore opening. Basic Res Cardiol. 2008;103:274–84.
27. Jones RG, Plas DR, Kubek S, et al. AMP-activated protein kinase induces a p53-dependent metabolic checkpoint. Mol Cell. 2005;18:283–93.
28. Halestrap AP, Clarke SJ, Javadov SA. Mitochondral permeability transition pore opening during myocardial reperfusion – a target for cardioprotection. Cardiovasc Res. 2004;61:372–85.
29. Xiao H, Ma X, Feng W, et al. Metformin attenuates cardiac fibrosis by inhibiting the $TGFB_1$-Smad3 signalling pathway. Cardiovasc Res. 2010;87:504–13.
30. Ishibashi Y, Matsui T, Takeuchi M, Yamagishi S. Beneficial effets of metformin and irbesartan on advanced glycation end products (AGEs)-RAGE-induced proximal tubular cell injury. Pharmacol Res. 2012;65:297–302.
31. Cuthbertson J, Patterson S, O'Harte FP, Bell PM. Investigation of the effect of oral metformin on dipeptidylpeptidase-4 (DPP-4) activity in Type 2 diabetes. Diabet Med. 2009;26:649–54.
32. Timmers L, Henriques JP, de Kleijn DP, et al. Exenatide reduces infarct size and improves cardiac function in a porcine model of ischemia and reperfusion injury. J Am Coll Cardiol. 2009;53:501–10.
33. Fischer Y, Thomas J, Rösen P, Kammermeier H. Action of metformin on glucose transport and glucose transporter GLUT1 and GLUT4 in heart muscle cells from healthy and diabetic rats. Endocrinology. 1995;136:412–20.
34. Van de Werf F, Bax J, Betriu A, et al. Management of acute myocardial infarction in patients presenting with persistent ST-segment elevation. Eur Heart J. 2008;29:2909–45.
35. Ploegstra MJ, Kampinga MA, Croon DH, Zijlstra F, van der Horst IC. Secondary prevention following myocardial nfarction may be improved. Long-term medication use according to practice guidelines. Ned Tijschr Geneeskd. 2010;154:A1971.
36. Götte MJ, Germans T, Russel IK, et al. Myocardial strain and torsion quantified by cardiovascular magnetic resonance tissue tagging. Studies in normal and impaired left ventricular function. J Am Coll Cardiol. 2006;48:2002–11.
37. van der Vleuten PA, Rasoul S, Huurnink et al. The importance of left ventricular function for long-term outcome after primary percutaneous coronary intervention. BMC Cardiovasc Disord. 2008;23:8:4.
38. Hartog JW, van der Wal RM, Schalkwijk CG, et al. Advanced glycation end-products, anti-hypertensive treatment and diastolic dysfunction in patients with hypertension and diastolic dysfunction. Eur J Heart Fail. 2010;12:397–403.
39. Sjauw KD, van der Horst IC, Nijsten MW, Nieuwland W, Zijlstra F. Value of routine admission laboratory tests to predict thirty-day mortality in patients with acute myocardial infarction. Am J Cardiol. 2006;97:1435–40.
40. van der Horst IC, de Boer RA, Hillege HL, Boomsma F, Voors AA, van Veldhuisen DJ. Neurohormonal profile of patients with heart failure and diabetes. Neth Heart J. 2010;18:190–6.
41. Timmer JR, Hoekstra M, Nijsten MW, et al. Prognostic value of admission glycosylated hemoglobin and glucose in nondiabetic patients with ST-segment-elevation myocardial infarction treated with percutaneuos coronary intervention. Circulation. 2011;124:704–11.

42. Van der Horst IC, Nijsten MW, Vogelzang M, Zijlstra F. Persistent hyperglycemia is an independent predictor of outcome in acute myocardial infarction. Cardiovasc Diabetol. 2007;6:2.
43. Standards of Medical Care. Diabetes Care. 2011;34:S11–61.
44. Stone GW, Maehara A, Witzenbichler B, et al. Intracoronary abciximab and aspiration thrombectomy in patients with large anterior myocardial infarction: the INFUSE-AMI randomized trial. JAMA. 2012;307:1817–26.
45. Lønborg J, Vejlstrup N, Kelbæk H, et al. Exenatide reduces reperfusion injury in patients with ST-segment elevation myocardial infarction. Eur Heart J. 2012;33:1491–9.
46. Sun Y, Weber KT. Infarct scar, a dynamic tissue. Cardiovascular Res. 2000;46:250–6.
