# Supplementary material for: Sex differences in cardiac structure and function following ST-segment elevation myocardial infarction
Source: Sci Rep. 2026 May 19;16:22756. doi: 10.1038/s41598-026-52993-8 (PMC13385960; doi:10.1038/s41598-026-52993-8)
Supplement: Supplementary file 4 — Supplementary Material 4 [file 41598_2026_52993_MOESM4_ESM.pdf]

RESEARCH PROTOCOL

Metabolic modulation with metformin to reduce heart failure after acute myocardial infarction: Glycometabolic Intervention as adjunct to Primary percutaneous coronary intervention in ST elevation myocardial infarction (GIPS-III)

A randomized controlled trial

Version # 6

Thorax Center, Department of Cardiology
University Medical Center Groningen (UMCG), University of Groningen, the Netherlands

PROTOCOL TITLE:

Metabolic modulation with metformin to reduce heart failure after acute myocardial infarction: Glycometabolic Intervention as adjunct to Primary percutaneous coronary intervention in ST elevation myocardial infarction (GIPS-III): a randomized controlled trial.

**Protocol ID**	**GIPS-III**
**Short title**	metformin to reduce heart failure after acute myocardial infarction
**Version**	**# 6**
**Date**	**01-02-2013**
**Principal investigator**	***Dr. I.C.C. van der Horst***<br>**Department of Cardiology, Thorax Center**<br>**UMCG, University of Groningen, The Netherlands**<br>**Telephone +31 50 3612355; Fax +31 50 3611347**<br>**Email : i.c.c.horst@umcg.nl**
**Coordinating investigator**	***Drs. C.P.H. Lexis***<br>**Department of Cardiology, Thorax Center**<br>**UMCG, University of Groningen, The Netherlands**<br>**Telephone +31 50 3613238; Fax +31 50 3611347**<br>***Email c.p.h.lexis@umcg.nl***
**Steering Committee**	***Dr. I.C.C. van der Horst; i.c.c.horst@thorax.umcg.nl (Chair)***<br>***Prof. Dr. D.J. van Veldhuisen; d.j.van.veldhuisen@thorax.umcg.nl***<br>***Dr. R.A. de Boer; r.a.de.boer@umcg.nl***<br>***Dr. P. van der Harst; p.van.der.harst@umcg.nl***<br>***Dr. E. Lipsic; e.lipsic@umcg.nl***<br>***Dr. A.N.A. van der Horst; a.n.a.van.der.horst@int.umcg.nl***<br>***Prof. Dr. B.H.R. Wolffenbuttel; bwo@umcg.nl***
**Independent physician**	***Dr. P.P. van Geel; p.p.van.geel@umcg.nl***<br>**Department of Cardiology, Thorax Center**<br>**UMCG, University of Groningen, The Netherlands**
**Catheterization laboratory**	***Dr. A.F.M. van den Heuvel; a.f.m.van.den.heuvel@umcg.nl*****Cardiac Catheterization Laboratory**<br>**UMCG, University of Groningen, The Netherlands**

Cardiology	*Prof. Dr. D.J. van Veldhuisen; d.j.van.veldhuisen@umcg.nl*<br>*Dr. R.A. de Boer; r.a.de.boer@umcg.nl*<br>*Dr. P. van der Harst; p.van.der.harst@umcg.nl*<br>Department of Cardiology, Thorax Center<br>UMCG, University of Groningen, The Netherlands
Endocrinology	*Prof. Dr. B.H.R. Wolffenbuttel; b.h.r.wolffenbuttel@umcg.nl*<br>*Dr. A.N.A. van der Horst; a.n.a.van.der.horst@umcg.nl*<br>Department of Endocrinology<br>UMCG, University of Groningen, The Netherlands
Laboratory sites	*A. Oosterhoff;a.oosterhoff@umcg.nl*<br>Department of Laboratory<br>UMCG, University of Groningen, The Netherlands
Pharmacy	*Drs. B.H.W. Molmans; b.m.w.molmans@umcg.nl*<br>Department of Pharmacy<br>UMCG, University of Groningen, The Netherlands
Neuro-imaging Center (NIC)	*Prof. dr. G.J. ter Horst; general director NIC*<br>g.j.ter.horst@umcg.nl<br>NIC, University of Groningen, The Netherlands
Core laboratory for MRI-	*Prof. dr. A.C van Rossum; ac.vrossum@vumc.nl*<br>*Dr. R. Nijveldt; r.nijveldt@vumc.nl*<br>Image Analysis Center VUmc<br>Department of Cardiology, VU Medical Center, Amsterdam
Core laboratory for Echocardiography	*Prof. dr. A.A. Voors; a.a.voors@umcg.nl*<br>*Y. Hummel; y.m.hummel@umcg.nl*<br>Echo corelab, UMCG, University of Groningen, The Netherlands
Subsidizing party	Van Buchem Stichting, Thorax Center, UMCG
Subsidizing party	*ZonMW*<br>Grant number: 95103007.<br>The Netherlands Organization for Health Research and Development, The Hague, the Netherlands.

PROTOCOL SIGNATURE SHEET

Name	Signature	Date
**Principal investigator:**<br>***Dr. I.C.C. van der Horst***<br>**Cardiologist**<br>**University Medical Center Groningen**		
**Coordinating investigator:**<br>***Drs. C.P.H. Lexis***<br>**Research physician**<br>**University Medical Center Groningen**		

TABLE OF CONTENTS

1. RATIONALE 11
2. OBJECTIVES 19
2.1 Primary objective 19
2.2 Secondary objectives 19
3. STUDY DESIGN 20
4. STUDY FLOW CHART 21
5. STUDY POPULATION 22
5.1 Population 22
5.2 Inclusion criteria 22
5.3 Exclusion criteria 22
6. RANDOMIZATION AND REGISTRATION 24
6.1 Randomization 24
6.2 Registration 25
7. TREATMENT OF SUBJECTS 26
7.1 Before PCI procedure 26
7.2 PCI procedure 26
7.3 After PCI procedure 26
7.4 The intervention 27
7.5 Co-intervention 27
8. INVESTIGATIONAL MEDICINAL PRODUCT 28
8.1 Name and description 28
8.2 Summary of findings from non-clinical studies 28
8.3 Summary of findings from clinical studies 28
8.4 Summary of known and potential risks and benefits 29
8.5 Description and justification of route of administration and dosage 30
8.6 Dosage and method of administration 30
8.7 Preparation and labeling of investigational medicinal product 31
8.8 Drug accountability 31
8.9 Compliance 32
9. OUTCOME PARAMETERS AND SUBGROUPS 33
9.1 Outcome parameters 33
9.1.1 Main study outcome parameter 33
9.1.2 Clinical secondary study outcome parameters 33
9.1.3 Tertiary study outcome parameters 35
9.2 Subgroups 36
9.3 Study procedures 36
9.3.1 Baseline characterisics and clinical outcome 36
9.3.2 Coronary angiography 37
9.3.3 Electrocardiography 37
9.3.4 Laboratory analysis 38

9.3.4.1. Enzymatic infarct size 38
9.3.4.2 Glycometabolic state 39
9.3.5 Skin autofluorescence 39
9.3.6 Cardiac magnetic resonance imaging 40
9.3.7 Blood pressure 41
9.3.8 NYHA Heart Failure score 41
9.3.9 Pregnancy test 41
9.4 Study visits 42
9.5 Withdrawal of individual subjects 44
9.5.1 Specific criteria for withdrawal of individual subjects 44
9.5.2 Replacement of individual subjects after withdrawal 44
9.5.3 Follow-up of subjects withdrawn from treatment 45
10. SAFETY ASPECTS AND SAFETY REPORTING 46
10.1 Safety aspects 46
10.1.1 Metformin 46
10.1.2 PCI procedure 46
10.2 Medical Research Involving Human Subjects Act (WMO) event 46
10.3 Adverse and serious adverse events 47
10.4 Follow-up of adverse events 47
10.5 Data safety monitoring board (DSMB) 48
10.6 Monitoring 48
10.6 Endpoint Adjudication Committee (EAC) 49
11. STATISTICAL ANALYSIS AND SAMPLE SIZE 50
11.1 Statistical analysis 50
11.2 Sample size 50
11.2.1 Sample size calculation 51
12. ETHICAL CONSIDERATIONS 52
12.1 Regulation statement 52
12.2 Informed consent 52
12.3 Insurance 52
12.4 Incentives 53
13. ADMINISTRATIVE ASPECTS 54
13.1 Handling and storage of data and documents 54
13.2 Subsidizing party 54
13.3 Amendments 54
13.4 Progress report 55
13.5 End of study report 55
13.6 Publication policy 55
14. APPENDIX 56
14.1 NYHA heart failure classification 56
14.2 Current guidelines on diagnosis of diabetes and prediabetes 56
15. REFERENCES 57
16. ATTACHMENTS 63

LIST OF ABBREVIATIONS AND RELEVANT DEFINITIONS

**ABR**	**ABR form - General assessment and registration form**
**ACE**	**Angiotensin-I converting enzyme**
**AGEs**	**Advanced glycation endproducts**
**AMP**	**Adenosine monophosphate**
**AMPK**	**AMP-activated protein kinase**
**ATP**	**Adenosine triphosphate**
**BMI**	**Body mass index**
**CABG**	**Coronary artery bypass grafting**
**CCU**	**Cardiac care unit**
**CK**	**Creatinin kinase**
**CK-MB**	**myocardial band fraction of CK**
**CMR**	**Cardiac magnetic resonance**
**CRF**	**Case report form**
**CRP**	**Chain reactive protein**
**CVD**	**Cardiovascular disease**
**DM**	**Diabetes mellitus**
**DPP-4**	**Dipeptidylpeptidase-4**
**ECG**	**Electrocardiogram**
**eNOS**	**Endothelial nitric oxide synthase**
**GFR**	**Glomerular filtration rate**
**GLP-1**	**Glucagon like protein-1**
**HDL**	**High-density Lipoprotein**
**HOMA-IR**	**Homeostasis model assessment of insulin resistance**
**hsCRP**	**High sensitivity CRP**
**IFCC**	**International Federation of Clinical Chemistry**
**LDH**	**Lactate dehydrogenase**
**LDL**	**Low-density lipoprotein**
**LGE**	**Late gadolinium-enhanced**
**LV**	**Left ventricle**
**LVEF**	**Left ventricular ejection fraction**
**MALA**	**Metformin-associated lactic acidosis**
**METC**	**Medical ethics trial committee**

**MBG**	**Myocardial blush grade**
**MI**	**Myocardial infarction**
**mPTP**	**Mitochondrial permeability transition pore**
**NYHA**	**New York Heart Association**
**PCI**	**Percutaneous coronary intervention**
**QCA**	**Quantitative coronary angiography**
**RAGE**	**Receptor for AGEs**
**RISK**	**Reperfusion injury salvation kinase**
**sMDRD**	**Simplified modification of diet in renal disease**
**STEMI**	**ST-elevation myocardial infarction**
**TEI**	**Transmural extent of infarction**
**TIMI**	**Thrombolysis in Myocardial Infarction**
**TNF-alfa**	**Tumor necrosis factor alfa**
**WBP**	**Personal Data Protection Act**
**WMO**	**Medical Research Involving Human Subjects Act**

SUMMARY

Rationale: Treatment of patients with acute myocardial infarction (MI) has development tremendously over the past decades. Despite the advances these patients remain a high risk population. The number of patients with metabolic disturbances among all patients is high, and after diagnostic test up to 45% of patients is diabetic. Patients with metabolic disturbances or diabetes are likely to develop heart failure. Among patients with diabetes heart failure is the number one cause of mortality. Several treatments including early reperfusion and angiotensin converting enzyme (ACE)-inhibitors aim to preserve ventricular function and thereby prevent the development of heart failure. For patients with diabetes it is known that metformin treatment is related to improved outcome, i.e. less macrovascular events and even a reduction of mortality. Until recently metformin treatment was considered to be contraindicated in patients with heart failure. In human studies the prevalence of heart failure among diabetics treated with metformin was lower than in patients treated with other glucose lowering drugs. In a recent experimental study it was observed that metformin treatment was related to higher left ventricular ejection fraction in an ischemic model.
Since metformin is related to a lower prevalence of heart failure and possibly may be related to preservation of left ventricular function after acute MI in experimental studies it is interesting to investigate the effect on left ventricular ejection fraction and outcome in patients with STEMI.
Objective: To evaluate the efficacy of metformin 500mg 2dd1 treatment compared with placebo 2dd1 in adjunction to optimal reperfusion therapy for acute MI on left ventricular ejection fraction at 4 months.
Study design: A randomized controlled, double blind, single center trial.
Study population/ intervention: A total of 380 patients with acute myocardial infarction after primary percutaneous coronary intervention (PCI) including thrombus aspiration and stenting and deemed amenable, by the investigator, to be treated with metformin 500 mg 2dd1 or placebo 2dd1. All patients will be treated according current guidelines.
Outcome measures: Primary endpoint measure is the difference in left ventricular ejection fraction between both groups as determined by cardiac magnetic resonance imaging (CMR) 4 months after randomization. Secondary outcome measures are clinical outcome – mortality, re-intervention, recurrent MI, hospitalization for heart failure – and myocardial infarct size measured with LGE CMR-imaging. In addition, the impact of metformin on new-onset diabetes, glycometabolic parameters, neurohormones and advanced glycation endproduct levels will be evaluated. Finally, safety of metformin therapy will be assessed with emphasis on metformin-associated lactic acidosis (MALA) and renal function.

Expected results: We hypothesize that treatment with metformin will prevent adverse left ventricle (LV) remodeling after MI. This will result in preserved LV function (assessed by CMR), and a lower rate of heart failure hospitalizations and mortality.

Keywords: acute myocardial infarction, heart failure, left ventricular ejection fraction, magnetic resonance imaging, metformin, diabetes

1. RATIONALE

Patients with impaired glucose tolerance or diabetes have a 2 to 4 fold higher risk of developing cardiovascular disease (CVD) (1). In patients with diabetes the prevalence of myocardial infarction and heart failure is higher than in individuals without diabetes. Cardiac death is the main cause of mortality in patients with diabetes (2). After diagnostic testing 25 to 45% of patients with an acute coronary syndrome have diabetes, whereas the prevalence of diabetes in the general population varies between 10 to 15% (3). Up to 65% of patients with an acute coronary syndrome have diabetes or metabolic disturbances (3).

Both after primary percutaneous coronary interventions (PCI) and coronary artery bypass grafting (CABG), patients with diabetes have a higher risk of cardiac death (4;5) and worse left ventricular function (6). Comparing patients with newly detected type 2 diabetes to patients with previously established diabetes, both groups have comparable proportions of re-infarction, stroke, and long-term mortality following acute myocardial infarction (4). Subdividing cardiac death, the rate of sudden cardiac death in patients with diabetes is comparable to patients without diabetes, whereas heart failure is the main cause of the attributive cardiac death in patients with diabetes (7).

In acute myocardial infarction, several therapies including early primary percutaneous coronary interventions and ACE-inhibitors aim to preserve ventricular function and thereby prevent the development of heart failure (3).

Patients with impaired left ventricular function after revascularization have worse prognosis (8). Patients with metabolic disturbances or diabetes are likely to develop heart failure (9;10). Moreover, increased admission glucose levels (11) and persistent hyperglycemia (12) are related to a higher

mortality in patients with acute MI, regardless of diabetic status. These elevated glucose levels are thought to reflect pre existent impaired glucose tolerance or increased physical stress.

Infarct size after myocardial infarction is comparable in patients with and without diabetes (2;13). However, patients with diabetes have a lower left ventricular ejection fraction (LVEF) (6) and more congestive heart failure after myocardial infarction (2). Left ventricular dysfunction in diabetics is not only caused by decreased systolic ventricular function. Diabetic patients have higher diastolic stiffness of the left ventricle, contributing to the decreased ventricular function in diabetics (14). Therefore, the decreased systolic and diastolic ventricular function and increased congestive heart failure observed in diabetes is apparently not the result of more ischemic injury. The decreased LVEF the can be the result of glycometabolic disturbances, including an increased utilization of free fatty acids, and impaired pre-conditioning causing myocardial cells to be more prone to ischemic and reperfusion injury (2). Patients with diabetes have decreased myocardial flow reserve, which is related to long-term glucose dysregulation (2). The presence of multi vessel disease is also associated with a limited recovery of myocardial function after MI. Extensive coronary artery disease, autonomic dysfunction and a high prevalence of hypertension and dyslipidemia have all been linked to the increased susceptibility of diabetic patients to develop heart failure (2). Furthermore, endothelial dysfunction resulting in increased vascular resistance, is not only associated with congestive heart failure but also with diabetes and glucose disturbances in the sub-diabetic range (3). Diabetic cardiomyopathy, which is induced by various mechanisms, also predisposes to heart failure.

Besides the left ventricular function, pro-thrombotic disturbances in the coagulation system and a higher prevalence of co-morbidity such as renal disease may also contribute to the adverse prognosis of diabetic patients.

Renal function is associated with mortality and hospitalization in patients with heart failure (15). Worsening renal function is a predictor for mortality and hospitalization (16), both in- and out-

hospital worsening of renal function (17). The glomerular filtration rate (GFR) measures the filtration capacity of the kidneys and is considered the best overall index of renal function. The sMDRD formula is an accurate formula for estimating GFR (18).

A different marker of renal function is albuminuria. Nagi et al (1993) demonstrated that metformin decreased urinary albumin excretion, implying that metformin can improve renal function (19).

NT-proBNP (a brain natriuretic peptide) is synthesized by and released from the myocardium in response to reduced left ventricular function, and can be used as a marker of heart failure (20).

The possible occurrence of a severe lactic acidosis is known as one of the adverse effects of metformin. In contrast with the huge number of treated patients, metformin-associated lactic acidosis (MALA) is rare, with an estimated incidence of three per 100,000 metformin-treated patients per year, but is associated with a high mortality rate, averaging 50% (21;22). In most cases, MALA occurs in a previously treated type 2 diabetic patient when a concurrent disease induces an acute renal failure leading to metformin accumulation. The association between metformin and decreased renal perfusion in MALA lead to the contraindication of metformin in patients with heart failure and decreased renal failure. However, the causative mechanism has been disputed by Lalau et al (2001) (23).

According to current guidelines metformin therapy is contraindicated in patients with acidosis, renal insufficiency or heart failure with New York Heart Association (NYHA) (see appendix, chapter 14) classification II or higher (www.fk.cvz.nl).

Treatment of patients with ST elevation myocardial infarction (STEMI) consists of early reperfusion, if possible by primary PCI (3). Additional treatment of hyperglycemia has been subject of multiple investigations. Studies on the role of intensive insulin treatment during acute MI so far could not show a beneficial effect on mortality, i.e. DIGAMI II, CREATE-ECLA, GIPS 1 and 2 and the HI-5 (24-28).

Interestingly, in the DIGAMI II glucose lowering by means of metformin did not influence mortality but was related to a lower proportion of non-fatal cardiovascular events (24). The latter finding is in agreement with the UKPDS, but in this study metformin did also have a mortality lowering effect, probably because of inclusion of more patients and longer follow-up (21). In an observation by Anselmino and colleagues, metformin-treated patients had a trend towards lower 1-year mortality, combined cardiovascular endpoints, and revascularization procedures while the use of sulfonylureas was neutral in this respect (29). In four retrospective, non-randomized cohort studies of diabetics with heart failure, treatment with metformin was associated with better outcome than treatment with other glucose lowering drugs (10;30-32).

Metformin decreases blood glucose by enhancing insulin sensitivity, inducing greater peripheral uptake of glucose, and decreasing hepatic glucose output while lowering plasma insulin concentrations (21;22). Furthermore, Salpeter et al (2008) demonstrated that metformin therapy decreased calculated insulin resistance, using the homeostasis model assessment of insulin resistance (HOMA-IR), by 23% (33). Metformin treatment resulted in a 30% reduction of diabetes related endpoints and mortality compared to other glucose lowering therapies that lower blood glucose to similar levels (21). Therefore the cardio protective effects of metformin can not be attributed to the glucose lowering effects alone (34).

Aguilar et al (2004) demonstrated that patients with newly diagnosed diabetes and patients with previously known diabetes had similarly increased adjusted risks of mortality and cardiovascular events (4). Salpeter et al (2008) demonstrated that in patients at risk for diabetes, metformin therapy resulted in a 40% reduction of the incidence of new onset diabetes with an absolute risk reduction of 6% (33), resulting in a number needed to treat of 17 patients in 1.8 years (33). Since patients with both new onset and previously known diabetes have worse prognosis compared to patients without diabetes, the ability of metformin to prevent diabetes can result in improved prognosis.

In experimental MI, it has recently been suggested that metformin indeed confers cardioprotection, both in acute myocardial ischemia and in post-MI heart failure (34-36). Some of these protective effects of metformin may go via the energy sensing adenosine monophosphate (AMP)-activated protein kinase (AMPK) and endothelial nitric oxide synthase (eNOS), however, precise mechanistic insights are lacking (34-36). AMPK has been shown to increase the phosphorylation and activity of eNOS, and has been shown to be an important regulator of diverse cellular pathways (37).

When activated, AMPK stimulates fatty acid oxidation (38), promotes glucose transport (39), accelerates intracellular glycolysis (40), and inhibits triglyceride (41) and protein synthesis (42). Since the myocardium can produce energy out of glucose, fatty acids and lactate, metformin may substantially improve cardiac energetics. Recently, Gundewar et al (2009) confirmed this by demonstrating that metformin improved cardiac energetics due to improved myocardial cell mitochondrial respiration and adenosine triphosphate (ATP) synthesis in a murine model of heart failure (34). Solskov et al (2008) demonstrated that a single dose of metformin resulted in an increased in myocardial AMPK activity and induced a reduction in myocardial infarct size (36). Both in diabetic and non-diabetic mice, Calvert et al (2008) demonstrated that administration of metformin before or at reperfusion decreased myocardial injury (35). Gundewar et al (2009) demonstrated that administration of metformin improved LV function and survival via AMPK-mediated pathways (34). They found that the cardioprotective effects of metformin were ablated in mice lacking functional AMPK and eNOS.

Chronic activation of AMPK phosphorylates transcription factors altering gene expression and modulates mitochondrial biogenesis. In vitro studies have shown that AMPK activation is a key mediator of the changes in substrate utilization during cardiac ischemia and functions to maintain energy homeostasis, cardiac function, and myocardial viability (34).

Elevated levels of chain reactive protein (CRP), interleukin-6 (IL-6), tumor necrosis factor alfa (TNF-alfa) and fibrinogen, biomarkers reflecting inflammation, have been demonstrated to be associated with the risk of cardiovascular mortality (43-53). High-sensitivity CRP (hsCRP), a marker for low-grade atherosclerotic subclinical chronic inflammation, is associated with cardiovascular death and heart failure (54).

Lund et al (2008) demonstrated that metformin was effective in reducing biomarkers reflecting inflammation in type 2 DM patients (43). De Jager et al (2005) demonstrated that metformin treatment was associated with improvement of endothelial function, unrelated to changes in glycaemic control (55). Metformin treatment did not improve chronic low-grade inflammation (55).

Accumulation of advanced glycation endproducts (AGEs) is related to the development of cardiac dysfunction in both diabetic patients (56-58) and patients without diabetes (56;59-62). Van Heerebeek et al (2008) demonstrated that diastolic dysfunction in diabetics is associated with increased deposition of AGEs (14). Tanaka et al (1999) demonstrated that metformin may be effective in the prevention of diabetic complications through directly inhibiting advanced glycation endproducts (AGEs) formation (63). In a canine model of diabetes, metformin decreased cross-linking of collagen by advanced glycation endproducts with consequent improvement in myocardial performance (64).

Ouslimani et al (2007) demonstrated that metformin was able to inhibit cell expression of the receptor for advanced glycation endproducts (RAGE) (65). Thus, metformin could be able to prevent the development of diabetic cardiomyopathy.

Administration of metformin at reperfusion reduces myocardial infarct size, both in diabetic and non-diabetic rats, via activation of the survival protein kinase Akt (66), which forms a component of the reperfusion injury salvage kinase (RISK) pathway (67), resulting in inhibition of mitochondrial

permeability transition pore (mPTP) (66). The mPTP, a non-specific channel of the mitochondrial inner membrane, mediates cardiomyocyte death (68).

Glucagon like protein-1 (GLP-1) is secreted in response to meal ingestion and stimulates insulin synthesis, pancreatic B-cell growth and proliferation, inhibition of gastric motility, decreased postprandial glucose concentration, and several extrapancreatic cardioprotective effects (69). GLP-1 is rapidly inactivated by dipeptidylpeptidase-4 (DPP-4). Recently, Cuthbertson et al (2009) demonstrated that metformin suppressed DPP-4 activity in fasting type 2 diabetic patients, resulting in elevated GLP-1 levels (70).

Other benefits of metformin, which may contribute to its cardiovascular effects, include stabilization of weight (71), improvement in lipid profile (22;33), increased fibrinolysis and reduced plasminogen activator inhibitor-1 (43).

Taken together, metabolic derangements in patients (with and without diabetes) after acute MI are related to impaired outcome. Treatment of diabetics with metformin is associated with beneficial outcome. The ability of metformin therapy to promote the activation of AMPK, the phosphorylation of eNOS, the reduction of biomarkers reflecting inflammation, the improvement of endothelial dysfunction, the inhibition of the formation of AGEs and RAGE, the inhibition of mPTP formation, the suppression of DPP-4 activity, and increase NO bioavailability provides numerous potential cardioprotective actions in the setting of heart failure, such as vasodilation and the inhibition of oxidative stress and apoptosis. All of these actions, in addition to the effects of NO on the mitochondria, could account for the improvements in left ventricular function following metformin treatment. In particular, the effects of eNOS on hemodynamics could play an important role in providing prolonged changes in afterload and coronary blood flow regulation, which could then promote left ventricular function and improve left ventricular ejection fraction.

The lack of hypoglycemia induced by metformin made it possible to treat non-diabetics with metformin in a study of prevention of diabetes. The prevalence of MALA is rare. With the exception of acidotic patients and patients with renal dysfunction, metformin treatment can safely be initiated in all patients. Based on data from experimental and clinical studies the effect of metformin on myocardial function, specifically left ventricular function, is of great interest. Since patients with diabetes after MI mainly die from heart failure, the effect of metformin on prevention of heart failure and mortality remains open for investigation.

Since metformin is related to a lower prevalence of mortality, heart failure and possibly even is related to preservation of left ventricular function after acute MI we will aim to investigate the effect of metformin treatment on left ventricular ejection fraction, biomarkers, hospitalization for heart failure and long-term mortality.

2. OBJECTIVES

2.1 Primary objective

The primary objective of the GIPS-III is to evaluate the efficacy of metformin treatment compared with placebo in adjunction to optimal reperfusion therapy for acute MI on left ventricular ejection fraction 4 months after primary PCI as measured with CMR-imaging.

2.2 Secondary objectives

Secondary study objectives include the investigation of the impact of metformin treatment on:

- the incidence of a cardiovascular event within 4 months and after long-term follow-up (all cause mortality, cardiovascular death, re-infarction, re-intervention (both re-PCI and CABG), stroke and combined endpoints) (see table 1);
- the incidence of hospitalization for heart failure within 4 months and after long-term follow-up;
- the prevalence of new onset diabetes and prediabetes as defined by the current guidelines ;
- laboratory parameters associated with improving outcome as assessed by glycometabolic state (e.g. fasting blood glucose, HbA1c, insulin, GLP-1, HOMA-IR), inflammatory state (e.g CRP, high-sensitivity CRP, TNF-alpha,Il-6), lipid and cholesterol spectrum (e.g. LDL, HDL, total cholesterol, triglycerides), advanced glycation endproducts (e.g AGEs), renal function (e.g hemoglobin, hematocrit, MDRD), and neurohormones (e.g NT-proBNP, plasma renin activity, aldosterone) and protein profiles (mRNA, DNA) before hospital discharge and at each outpatient clinic visit.
- Infarct size and TEI using LGE CMR imaging 4 months follow-up.
- Electrocardiographic determinants of myocardial perfusion (incidence of new Q waves, ST-segment resolution, persistent ST-deviation) during hospitalization and each outpatient clinic visit.
- systolic and diastolic function using echocardiographic parameters during hospitalization and at 4 months follow-up.
- Body Mass Index (BMI) before hospital discharge and at each outpatient clinic visit.

3. STUDY DESIGN

This is a single-center, prospective, randomized double blind study. In total, 380 patients with a first acute MI will be included. All patients will be randomly assigned to receive metformin 500mg 2dd1 or placebo 2dd1 on top of standard medical care. The study will take place at the University Medical Center of Groningen, a center with experience in primary PCI of patients with acute MI and with access to emergency cardiac surgery.

CMR-imaging is performed to determine LVEF four months after PCI, a period in which the remodeling of the heart has completed. CMR is a well-recognized, validated, and highly reproducible technique. After long-term follow-up, metabolic, neurohormonal and novel markers, clinical endpoints, and left ventricular function will be assessed.

4. STUDY FLOW CHART

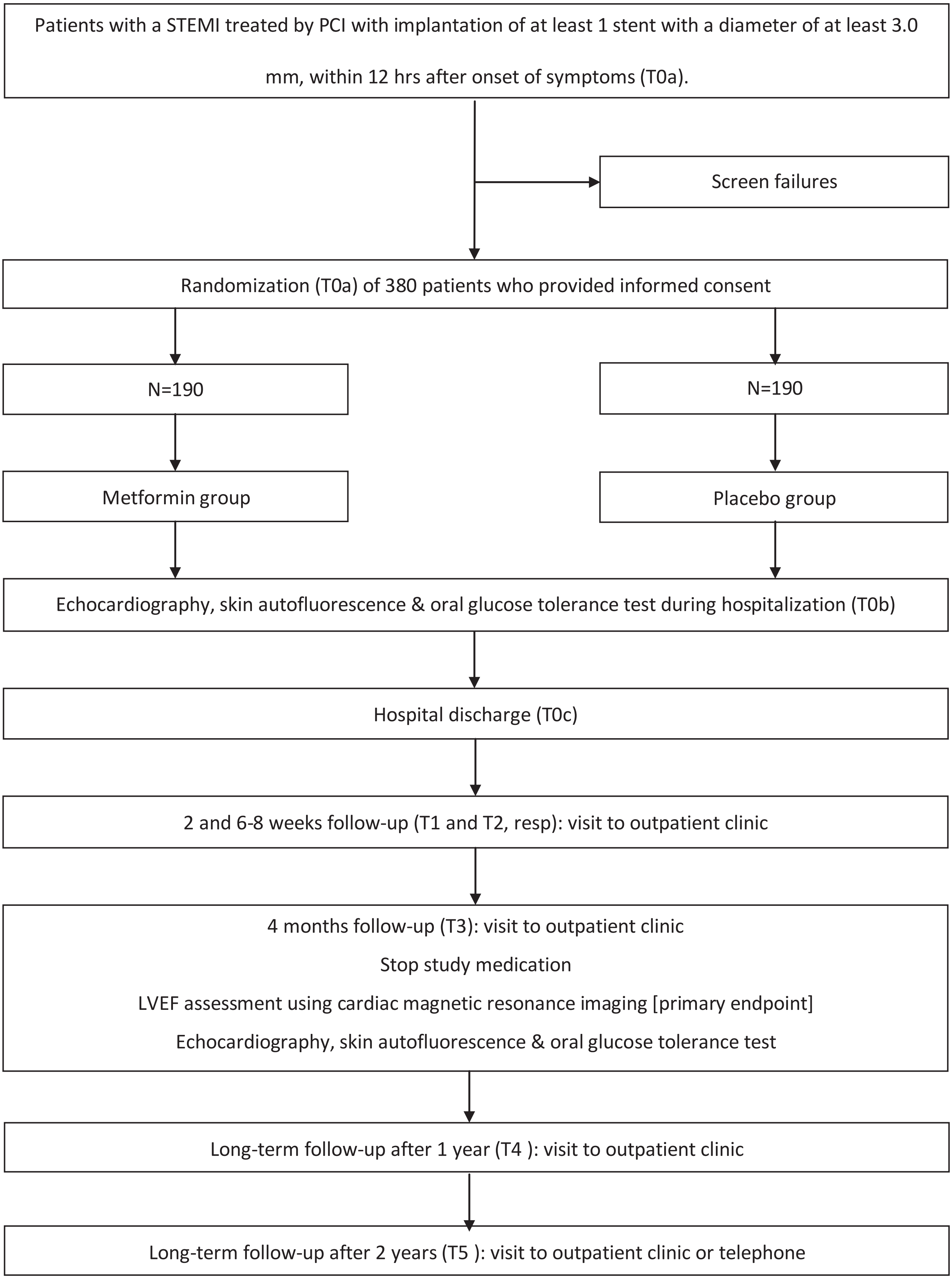

5. STUDY POPULATION

5.1 Population

All patients with an acute STEMI admitted to the University Medical Center of Groningen who are amenable to PCI are considered for participation in the study. During the PCI procedure, patients will be asked for their verbal informed consent by the treating cardiologist and catheterization laboratory nurse (independent witness). Patients will receive the study drug as soon as possible after PCI, but at least within 3 hours after PCI. Prior to administration of the first gift, the nurse at the CCU administrating the first dose will verify if patients still want to participate, and the patient information letter is handed over to the patient. Time of treatment will be recorded by the CCU nurse and verified by the investigator. In a later stadium following the PCI procedure, patients will sign the written informed consent.

5.2 Inclusion criteria

Inclusion criteria are:

- The diagnosis acute MI defined by chest pain suggestive for myocardial ischemia for at least 30 minutes, the time from onset of the symptoms less than 12 hours before hospital admission, and an ECG recording with ST- segment elevation of more than 0.1 mV in 2 or more leads.
- Successful primary PCI (post-procedural TIMI 2/3);
- At least one stent sized ≥ 3.0 mm;
- eligible for CMR-scan:
  - BMI <40;
  - no metal objects in the body (pacemaker, ICD, etc);
  - no claustrophobia;
- Verbal followed by written informed consent.

5.3 Exclusion criteria

Patients will be excluded from this study for any of the following reasons:

- Rescue PCI after thrombolytic therapy;
- Need for emergency coronary artery bypass grafting;
- Kreatinin >177 µmol/L measured pre-PCI;
- General condition which, according to the clinical judgment of the investigator and/or treating physician, does not allow the patient to participate in the study;
- Inability to provide informed consent;
- Younger than 18 years;
- Mechanical ventilation;
- Diabetes;
- Prior myocardial infarction;
- Contra-indication to metformin (see safety);
- The existence of a life-threatening disease with a life-expectancy of less than 6 months.

6. RANDOMIZATION AND REGISTRATION

6.1 Randomization

The Department of Pharmacy will supply the medication per patient blinded to both investigator and patient. Every set of medication will have a number. This number corresponds to the case report form (CRF) number. This CRF number will be the identification of the patient throughout the trial. The number for the study medication assignment must be recorded in the subject case report form (CRF). In the event a subject drops out prior to randomization, the subject's number will be re-used. Once a set of medication is randomly assigned to a patient, it will never be re-used. Randomization records are never re-used. Randomization data are kept strictly confidential; and are accessible only to authorized persons (B.H.W. Molmans, Department of Pharmacy), until the time of unblinding. Only when all patients have reached the primary efficacy measure (endpoint), the data file verified and the protocol violations determined, the database will be locked, after which the drug codes will be broken and made available for data analysis. Independent physicians and/or research nurses, who are blinded for treatment allocation, will perform follow-up visits T4 and T5. The endpoint adjudication committee will, who also will remain blinded after database lock, will assess all clinical endpoints. Analysis of data collected after database lock during will be done when all patients have reached a visit (either T4 or T5).

Unblinding of treatment in case of emergency can be done via the in-house pharmacy. An exception for unblinding the data will be made for the DSMB (for details: see attachment: DSMB charter). The contact procedures/results prior to unblinding, the events precipitating the unblinding, necessary contacts thereafter, and study status of the subject whose trial treatment was disclosed must be documented by the investigator and maintained in the study files.

Randomization will be performed by Stichting Apotheek Haagse Ziekenhuizen. In 63 blocks of 6 and 1 block of 2 patients, metformin 500mg 2dd1 and placebo 2dd1 will be randomized. After the PCI procedure and after verbal informed consent, patients will receive the study medication (metformin

500mg 2dd1 or placebo 2dd1) double blind. The investigator confirms the randomization by recording it in a dedicated listing which will be archieved in the Investigator Site File.

6.2 Registration

Selected data of potential candidates will be collected in a dedicated computerized database (Oracle Clincal ©). The trial coordination center will provide this computerized database. Data will be inserted in the database by cardiologists and research personnel. Data will be collected during initial hospitalization, from patient files, by telephone contacts, and from outpatient's visits. The database will contain baseline characteristics, interventional data, medication use, ECG-characteristics, CMR characteristics, laboratory data and clinical data. The database will be managed by the trial coordination center and the researchers.

7. TREATMENT OF SUBJECTS

7.1 Before PCI

In the phase before admission to the catheterization laboratory the patient will receive the following medical therapy according to current international guidelines:

- aspirin (a bolus of 300 mg if not already being taken, followed by 80 to 100mg/24 hours);
- intravenous heparin/low molecular weight heparin according to a scheme based on weight;
- thienopyridines;
- glycoprotein IIb/IIIa inhibitor: according to a scheme based on weight.

7.2 PCI procedure

All patients will be transported to the catheterization laboratory as soon as possible after admission to the hospital. At admission blood will be drawn from the patient by vena punction for standard lab analysis. After gaining arterial access at the catheterization laboratory, both coronary arteries will be visualized. If the coronary anatomy of the culprit lesion is suitable for percutaneous intervention, PCI will be performed. Primary treatment of the ischemia related coronary artery will consist of thrombus aspiration followed by stent implantation (at least one stent sized ≥ 3.0mm). Additional balloon angioplasty will be performed when necessary. Thrombus aspiration will be performed using an aspiration device (CE, Medtronic Corporation, Santa Rosa, California, USA).

7.3 After PCI procedure

Standard therapies after PCI will be administered according to international guidelines. Standard therapies include aspirin 80 or 100mg, thienopyridines, beta-blockers, lipid lowering agents (HMG-CoA-reductase inhibitors), and angiotensin converting enzyme inhibitors or angiotensin-II receptor blockers. These medications are likely to be protective in most patients.

Patients with new-onset diabetes will be treated according to international guidelines.

All medication used will be recorded in the eCRF (Oracle Clinical ©).

7.4 Investigational product/treatment

After successful PCI patients will be transferred to the CCU. After arrival all inclusion and exclusion criteria will be assessed, especially kreatinin concentration. Metformin tablets will be administered orally at 500 mg twice daily for 4 months or matching placebo tablets will be administered twice daily, blinded to both patients and cardiologists.

7.5 Co-intervention

Co-intervention is not applicable. No specific restrictions to co-medication apply. Permitted co-medication includes e.g. stable, appropriate pharmacologic therapy for coronary artery disease, heart failure or heart rhythm disorders (e.g. angiotensin converting enzyme inhibitors, angiotensin receptor blockers, beta blockers, anticoagulant drugs, platelet inhibitors, calcium antagonists, anti-arrhythmics, diuretics, HMG-CoA reductase inhibitors, nitrates, alfa blockers). The investigator may change the dose or add/discontinue any of the medications identified above if in the opinion of the investigator such a change is medically necessary. Cardiovascular medication at hospital admission, at hospital discharge, and during the outpatient clinic visits will be recorded in the eCRF.

8. INVESTIGATIONAL MEDICINAL PRODUCT

8.1 Name and description

Metformin, a biguanide hypoglycaemic agent used in the treatment of type 2 DM, improves glycaemic control by improving insulin sensitivity and decreasing intestinal absorption of glucose.

Metformin (hydrochloride) is a registered medicinal product (see attachment Summary of Product Characteristics).

The study medication, both metformin and placebo, will be provided by Stichting Apotheek Haagse Ziekenhuizen. For the metformin a Summary of Product Characteristics form is added as an attachment. An Investigational Medical Product Dossier of the placebo Is also added by Stichting Apotheek Haagse Ziekenhuizen.

8.2 Summary of findings from non-clinical studies

The ability of metformin therapy to promote the activation of AMPK, the phosphorylation of eNOS, the reduction of biomarkers reflecting inflammation, the improvement of endothelial dysfunction, the inhibition of the formation of AGEs and RAGE, the inhibition of mPTP formation, the suppression of DPP-4 activity, and increase NO bioavailability provides numerous potential cardioprotective actions in the setting of heart failure, such as vasodilatation and the inhibition of oxidative stress and apoptosis. All of these actions, in addition to the effects of NO on the mitochondria, could account for the improvements in left ventricular function following metformin treatment. In particular, the effects of eNOS on hemodynamics could play an important role in providing prolonged changes in afterload and coronary blood flow regulation, which could then promote left ventricular function and improve left ventricular ejection fraction.

8.3 Summary of findings from clinical studies

Metformin decreases blood glucose by enhancing insulin sensitivity, inducing greater peripheral uptake of glucose, and decreasing hepatic glucose output while lowering plasma insulin

concentrations (21;22). In the DIGAMI II glucose lowering by means of metformin did not influence mortality but was related to a lower proportion of non-fatal cardiovascular events (24). The latter finding is in agreement with the UKPDS, but in this study metformin did also have a mortality lowering effect, probably because of inclusion of more patients and longer follow-up (21). In an observation by Anselmino and colleagues, metformin-treated patients had a trend towards lower 1-year mortality, combined cardiovascular endpoints, and revascularization procedures while the use of sulfonylureas was neutral in this respect (29). In four retrospective, non-randomized cohort studies of diabetics with heart failure, treatment with metformin was associated with better outcome than treatment with other glucose lowering drugs (10;30-32).

Metformin treatment resulted in a 30% reduction of diabetes related endpoints and mortality compared to other glucose lowering therapies, lowering blood glucose to equal levels (21). Thus, the cardioprotective effects of metformin can not be attributed to glucose lowering effects alone (34).

8.4 Summary of known and potential risks and benefits

The possible occurrence of a severe lactic acidosis is known as one of the adverse effects of metformin. In contrast with the huge number of treated patients, metformin-associated lactic acidosis (MALA) is rare, with an estimated incidence of three per 100,000 metformin-treated patients per year, but is associated with a high mortality rate, averaging 50% (21;22). In most cases, MALA occurs in a previously treated type 2 diabetic patient when a concurrent disease induces an acute renal failure leading to metformin accumulation. The association between metformin and decreased renal perfusion in MALA lead to the contraindication of metformin in patients with heart failure and decreased renal failure. The causative mechanism has been disputed by Lalau et al (2001) (23).

According to current guidelines metformin therapy is contraindicated in patients with acidosis, renal insufficiency or heart failure with NYHA classification II or higher (www.fk.cvz.nl).

8.5 Description and justification of route of administration and dosage

The route of administration of the metformin 500 mg 2dd1 tablets is oral. The bioavailability is approximately 50 to 60 %. Withdrawal of study drug can be considered if the study drug is not tolerated or otherwise indicated in a specific patient (see withdrawal criteria). No other modifications to study drug can be made.

Normal starting dosage of metformin for treatment of diabetes type II varies from 500mg once daily to 500mg twice daily. Maximum dosage of metformin for treatment of diabetes type II is 3000mg divided over 3 gifts. In our study protocol we chose a dose of 500mg administered twice daily. Based on figures from the EASD 2009 guidelines (3) metformin leads to side effects, most all gastro-intestinal discomfort, in 30% of patients and results in discontinuation of 2-5% of patients started on metformin therapy. The side effects are, equal to the antihyperglycaemic effects, dose-dependent. Since the time window of administration of metformin is relatively short (4 months) for titration, metformin will be dosed in one uniform dosage. Most importantly, there is no evidence for a dose-dependent relation between metformin and the supposed cardioprotective effects. The dosage of metformin administered in the animal experiments (34-36) was high (metformin 250mg/kg/ml) compared to dosages normally administered in humans (8-45mg/kg/day). However, animal experiments demonstrated that relatively high dosages of metformin were necessary for the drug to exert its antihyperglycaemic effects in hyperglycaemic rats. Therefore, it might be deducted that the non-dose-dependent cardioprotective effects of metformin show at a dosage of 500mg metformin twice daily.

8.6 Dosages and method of administration

Metformin 500mg 2dd1 tablet oral.

8.7 Preparation and labeling of Investigational Medical Product

A white film coated double convex tablet formation of metformin hydrochloride (see attachments) in strengths of 500mg and a visually matching placebo tablet will be used for the 4 months double-blind treatment phase of the study. All study drugs will be supplied by Stichting Apotheek Haagse Ziekenhuizen and released for use according to Stichting Apotheek Haagse Ziekenhuizen standard operating procedures. All study drugs should be stored at room temperature of 20 to 25 degrees Celsius. The drugs will be dispensed during hospital admission and at the visit to the outpatient clinic at 6 weeks after randomization. Each treatment kit will contain blister cards, each containing a 1 week supply of study drug (14 tablets). Study drug labeling will include the following: sponsor's name, sponsor's address, sponsor's telephone number, route of administration, content ("metformin 500mg or placebo"), number of tablets, expiration date, "keep out of reach of children", "clinical trial medication", batch code, protocol number, name and address of principal investigator, patient number, and directions for use; according to Annex 13 of Good Manufacturing Practice. The study drug label will be supplied by Stichting Apotheek Haagse Ziekenhuizen (see attachment)

8.8 Drug accountability

The investigator will accurately record the amount of study drug received, dispensed, consumed or administered, and returning during the study period. An accurate record of the date and amount of the study drug dispensed to each subject must be available for inspection at any time. Dispensed medication will be recorded in a dedicated folder. Drug supply accountability, based on tablet counts, will be performed at the conclusion of the study, recorded on the appropriate CRF and the subject's source documentation. All unused study drug will be returned to the Department of Pharmacy of the UMCG, to destroy the study drugs according to accepted standards of Good Clinical Practice, the protocol and all applicable government laws and regulations and ICH guidelines governing the performance of clinical investigations. Drug accountability will be reviewed by the Department of Pharmacy of the UMCG during site visits and at the completion of the trial. The

investigator must not destroy any drug labels, or any partly-used or unused drug supply. All drug supplies are to be used only for this protocol and not for any other purpose. Partly-used or unused study drug will be destroyed in accordance with local, state, and federal regulations.

8.9 Compliance

Patients that used >80% of the study medication they were supposed to use, are considered to be compliant. A protocol violation will be reported if patients are non-compliant.

9. OUTCOME PARAMETERS AND SUBGROUPS

9.1 Outcome parameters

9.1.1 Main study outcome parameter

The primary endpoint (primary efficacy parameter) is to evaluate the effect of metformin treatment (in the intention-to-treat analysis) after a successful PCI for a first acute MI on left ventricular ejection fraction measured with CMR imaging at 4 months after the PCI procedure (as described in detail).

9.1.2 Clinical secondary study outcome parameters

Incidence of a cardiovascular event within 4 months and after long-term follow-up: defined as cardiovascular death, re-infarction (STEMI or Non-STEMI), re-intervention by PCI (target leasion revascularization, target vessel revascularization, or non-target vessel revascularization) or CABG, stroke (CVA, no TIA) and combined endpoints. MACE is defined as death, re-infarction and target laesion revascularisation.

Mortality will be divided into cardiac and non-cardiac. Cardiac death will be divided into three categories: heart failure, sudden death and other. A cardiologist will confirm deaths from cardiovascular causes by examining medical records obtained from hospitals and attending physicians or from attending general practitioner if the patient died at home. Sudden cardiac death will be defined as either witnessed, or un-witnessed, cardiac arrest without evidence of circulatory collapse, such as hypotension, exacerbation of congestive heart failure, or altered mental status, before the disappearance of the pulse or abrupt collapse occurring within one hour of the onset of the symptoms that resulted in death. Death due to heart failure will be defined as death due to clinically end-stage heart failure during hospital admission or by exacerbation of congestive heart failure reported by an attending general practitioner. For all these deaths, no probable non-cardiac cause should be suggested by the history or autopsy.

CLINICAL SECONDARY ENDPOINT	CLASSIFICATION	SUBGROUP
Death	1	
- Cardiac		A
- All Cause		B
Recurrent myocardial infarction	2	
- STEMI		A
- Non-STEMI		B
Recurrent coronary intervention	3	
- target lesion PCI		A
- target vessel PCI		B
- non target vessel PCI		C
- CABG		D
Cerebrovascular accident (CVA)	4	
Non-elective hospitalisation for:	5	
- Heart failure		A
- chest pain		B
ICD implantation	6	
New onset diabetes	7	
Prediabetes	8	

Tabel 1: Clinical secondary endpoints.

A non-elective hospitalization (defined as an overnight stay, with different dates for admission and discharge) for clear symptoms of heart failure within 4 months and after long-term follow-up will be recorded. Furthermore, non-elective hospitalization (defined as an overnight stay, with different dates for admission and discharge) for chest pain will be recorded as an endpoint. Implantation of an internal cardiac defibrillator (ICD) will be recorded as an endpoint. Hospitalization for other reasons than heart failure or chest pain will not be recorded as an endpoint. Whenever a patient undergoes an elective PCI, CABG, or ICD implantation, or has a recurrent myocardial infarction or stroke, the patient is (likely to be) hospitalized. Therefore, in case of these events, the hospitalization itself will not be recorded as a separate endpoint. New onset diabetes and prediabetes, as defined by the guidelines (see Appendix 14.2) will be recorded as an endpoint. Whenever new onset diabetes is

diagnosed, patients will be treated by an endocrinologist at University Medical Center Groningen according to current guidelines. Treatment for diabetes will be additional to study treatment. Visits for treatment will be separate from study visits.

9.1.3 Tertiary study outcome parameters

- Glycometabolic state (fasting blood glucose, HbA1c, insulin, GLP-1, DPP-4), inflammatory state (CRP, high-sensitivity CRP, TNF-alpha, etc), renal function (hemoglobin, hematocrit, urinary albumin excretion, creatinin, urea, sMDRD), lipid and cholesterol spectrum (LDL, HDL, total cholesterol, triglycerides), levels of advanced glycation endproducts (AGEs) measured in blood and by skin autofluorescence, neurohormones (NT-proBNP, plasma renin activity, aldosterone, etc), and protein profiles (e.g. mRNA) will be determined before hospital discharge and at each outpatient clinic visit to investigate the relationship of these factors with the intervention. Furthermore, the impact of these factors on LVEF, cardiovascular endpoints, and heart failure will be determined.

- Infarct size, TEI, functional MRI, and local contractility will be determined using LGE CMR imaging at 4 months follow-up. The impact of infarct size and TEI on LVEF, cardiovascular endpoints, and heart failure will be determined.

- The presence of pathologic Q waves on the ECG will be monitored before hospital discharge and at each outpatient clinic visit.

- Echocardiographic parameters of systolic and diastolic function, and valvular functions during hospitalization and at 4 months follow-up.

- Body Mass Index will be measured before hospital discharge and at each outpatient clinic visit.

- Safety and serious adverse event monitoring, i.e. the prevalence of lactic acidosis and worsening renal failure will be monitored during hospital admission, before hospital discharge and at each outpatient clinic visit.

9.2 Subgroups

The primary and secondary endpoints will be analyzed in prespecified subgroups defined by:

- gender;
- age;
- BMI;
- location of myocardial infarction;
- physical diagnostics;
- ECG characteristics;
- laboratory findings;
- CMR-imaging findings.

Furthermore, we will perform "per protocol" analyses restricted to compliant patients and who did not receive 'open label' anti-hyperglycaemic strategies for clinical reasons (e.g. developed diabetes during the study).

9.3 Study procedures

9.3.1 Baseline characteristics and clinical outcome

Baseline characteristics that will be collected include age, gender, time of symptom onset of first and last symptoms, time of admission, history of CABG, previous PCI, stroke and MI, positive family history for cardiovascular diseases, existence of diabetes, hypertension, smoking status, heart rate,

systolic and diastolic blood pressure, weight and length. Current treatment at admission of the patients to the hospital will be recorded.

Death, reinfarction, and ischemia driven target vessel revascularization will be recorded at 30 days and at 4 months and after long-term follow-up. Follow-up information will be obtained from hospital records, written correspondence and/or telephone interviews with the patient.

9.3.2 Coronary angiography

A coronary angiogram will be obtained at baseline. PCI of the ischemia related artery will be performed if indicated according to current protocols (possibly thrombus aspiration, balloon angioplasty and/or stenting, using bare metal stents or drug eluting stents). After the PCI procedure, a control coronary angiogram will be performed. Intravenously administered nitroglycerine will be given after the procedure and before the last angiogram in all patients.

The following baseline and post-procedural angiographic features will be recorded: Quantitative Coronary Angiography (QCA) data (reference diameter, minimal lumen diameter, and diameter stenosis, lesion length), the presence of thrombus, Thrombolysis In Myocardial Infarction (TIMI) flow grades, myocardial blush grade (MBG), and the presence of distal embolization.

QCA data and TIMI flow grades will be measured as previously described (72;73). The evaluation of MBG will be performed as described by Van ’t Hof et al: 0, no myocardial blush; 1, minimal myocardial blush or contrast density; 2, moderate myocardial blush or contrast density, but less than that obtained during angiography of a contra- or ipsilateral non-infarct related coronary artery; and 3, normal myocardial blush or contrast density, comparable with that obtained during angiography of a contra- or ipsilateral non -ischemia-related coronary artery (74). Persisting myocardial blush (“staining”) suggests leakage of contrast medium into the extravascular space and is graded 0. Next too MBG evaluation by the interventional cardiologist, MBG may also be assessed using the

Quantifying Blush Evaluator (QuBE), a computer program. QuBE has been demonstrated to provide practical, freely available, computer assessment of myocardial perfusion (75).

9.3.3 Electrocardiography

A standard 12-lead electrocardiogram is acquired at the time of presentation, after the PCI procedure, before hospital discharge, and at each outpatient clinic visit. Mean time interval between pre and post intervention will be registered. The ECG was recorded at a speed of 25 mm/s and a voltage of 10mm/mV. Q and R durations were measured in milliseconds along the PR baseline (1mm = 40ms), and the R amplitude in millivolts (1mm = 0.1 mV).Pre-intervention ECG will be analyzed on the presence of ST-deviation. The post-intervention ECG will be used to score the incidence and location of new Q-waves.

Electrocardiographic criteria of sufficient duration and amplitude for pathologic Q-waves were to meet the lead-specific standards of the Minnesota Code (codes 1-1 through 1-2, except 1-2-8) [Minnesota]. Additionally, the following criteria were used: Q wave ≥ 30ms on lead aVF, Q wave ≥30 ms on leads I and aVL, Q wave ≥30 ms in ≥2 on leads V4 to V6, R wave ≥40 ms in lead V1, any Q wave in lead V2, and R wave ≤0.1 mV and ≤10 ms on lead V2 according to Selvester's QRS screening criteria for Q-wave myocardial infarction.

Two observers blinded to study randomization will analyze all ECG recordings.

9.3.4 Laboratory analysis

Laboratory testing is subdivided in standard (according to current guidelines) laboratory tests, which are commonly assessed for standard patient care, and additional laboratory tests. These additional tests include markers of inflammation and fibrosis, glycometabolic state, trombogenic state, and

markers of heart failure. An overview of the laboratory analysis is added as an attachment (in Dutch). Enzymatic infarct size and glycometabolic state are discussed separately.

9.3.4.1 Enzymatic infarct size

Infarct size will be estimated by serial measurements of cardiac markers in serum, including creatinin kinase (CK), myocardial band fraction of CK (CK-MB), lactate dehydrogenase (LDH), and troponin T. The first measurement is taken as soon as possible after admission. Thereafter, frequent marker determinations are performed according to a schedule that calls for 4 to 6 measurements in the first 24-36 hours. To accommodate the problem in practice that exact predefined times for blood sampling are not always followed, the actual times of sampling, expressed as minutes after the moment of randomization, are recorded. To allow optimal comparison of the time courses of marker levels and to best approximate the area under the marker level curves, we use a dedicated algorithm (76;77). Per patient the algorithm interpolates the marker levels based on the precise time of measurement. Next, it determines for each of the predefined intervals if an actual measurement has been performed in that interval. Marker levels are determined on a Hitachi 717 automatic analyzer according to the International Federation of Clinical Chemistry (IFCC) recommendation, at 30 degrees Celsius. A peak marker release above the 75% percentile (i.e. the highest quartile) is defined as high enzyme release. Time to peak release is also determined.

9.3.4.2 Glycometabolic state

Patients with known diabetes are those reporting on an established diagnosis or those on glucose lowering medication, and will be excluded per protocol. The study protocol asks for fasting plasma glucose and glycosylated hemoglobin in patients with unknown glycometabolic state. The glycometabolic classification, performed at hospitalization, in normal glucose regulation, impaired glucose regulation (impaired fasting glucose and/or impaired glucose tolerance), and newly detected diabetes will be based on fasting plasma glucose and glycosylated hemoglobin according to

definitions in the guidelines. In addition, an oral glucose tolerance test (OGTT) will be performed to detect the sensitivity for glucose administration.

If diabetes or prediabetes is diagnosed, according to the current guidelines (See appendix 14.2), it will be recorded as an adverse event and reported as an endpoint.

9.3.5 Skin autofluorescence

Tissue AGE accumulation was assessed using a validated skin autofluorescence (AF) reader (advanced glycation end-products reader; patent PCT/NL99/00607;DiagnOptics BV, Groningen, The Netherlands). The AGE-reader illuminates a skin surface of approximately 2 cm^2, guarded against surrounding light, with an excitation light source between 300-420 nm (peak excitation ~370 nm) Light from the skin is measured with a spectrometer in the 420-600 nm range, using 200 μm glass fibers. The value of skin AF is calculated as the ratio of the light intensity in the 420-600 nm wavelength range and the light intensity in the 300-420 nm wavelength range. Meerwaldt et al have shown that repeated skin AF measurements on one day show an overall Altman error rate of 5.03% [78]. Intraindividual seasonal variance shows an Altman error rate of 5.87% [78]. The difference between repeated measurements does not alter depending on skin AF level [78]. Skin AF will be measured at the volar side of the lower arm at approximately 10 to 15 cm below the elbow fold. The measurement was performed three times at a healthy skin site (i.e. without visible vessels, scars or other skin abnormalities) and an average was calculated. Skin AF will be measured during hospitalization, and 4 months after randomization.

9.3.6 Cardiac magnetic resonance imaging

To determine LVEF, cardiac magnetic resonance imaging (CMR) will be performed. CMR examination will be performed on a 3 Tesla clinical scanner (3T Achieva, Philips, Best, the Netherlands) at the Neuro-imaging Center (general director: prof. dr. G.J. ter Horst) using a phased array cardiac receiver coil at 4 months after primary PCI. Electrocardiogram-gated images are acquired during repeated

breath-holds of approximately 10 seconds. LV function is determined with cine imaging, using a segmented steady state free precession pulse sequence in multiple short axis views every 10 mm covering the entire left ventricle. Typical in plane resolution was 1.6 x 1.9 mm2, with slice thickness of 6.0 mm (repetition time/echo time = 3.2/1.6 ms, flip angle 60°, matrix 256 x 156, temporal resolution 35 - 50 ms). Late gadolinium-enhanced (LGE) images are acquired to determine infarct size and transmural extent of infarction. A 2D segmented inversion recovery gradient-echo pulse sequence will be used, 15 minutes after administration of a gadolinium-based contrast agent (Dotarem, Guerbet, Roissy, France) (0.2 mmol/kg), with slice locations identical to the cine images. Typical in plane resolution is 1.4 x 1.8 mm2, with slice thickness of 6.0 mm (repetition time/echo time = 9.6/4.4 ms, flip angle 25°, matrix 256 x 166, triggering to every other heart beat). The inversion time will be set to null the signal of viable myocardium. All MRI data are sent anonymously to independent cardiologists, who are blinded for randomization status, for quality control and blinded analysis.

9.3.7 Blood pressure

Blood pressure will be measured in a seated position during physical examination using a standard mercury sphygmomanometer.

9.3.8 NYHA Heart Failure score

The study physician will determine the NYHA heart failure classification score according to the criteria (see appendix, chapter 14).

9.3.9 Pregnancy test

A pregnancy test, using serum human chorionic gonadotropin (HCG), will be measured in all female patients with childbearing potential (female patients of 60 years and younger) during hospital

admission and we will perform pregnancy tests during outpatient hospital visits when there is a reasonable suspicion of pregnancy.

9.4 Study visits

The study visits are summarized in the study flow chart (chapter 4, page 21). All efforts will be made to perform study visits as scheduled. However, a 5-day margin will be allowed for visits T1 and T2, and a 10-day margin will be allowed for visits T3, T4 and T5. All study procedures will have to be performed during study visit as scheduled, with the exception of the CMR-scan. The CMR-scan may be performed 10 days before visit T3.

- During hospitalization (T0)

  - inclusion/exclusion;
  - primary PCI (see inclusion criteria);
  - verbal followed by written informed consent;
  - randomization;
  - dispense study drug;
  - NYHA heart failure score (see appendix, chapter 14)
  - medical history;
  - concomitant medication;
  - ECG;
  - echocardiography (diastolic function);
  - skin autofluorescence;
  - serious adverse events recording;
  - clinical examination (blood pressure, heart rate, length, weight,);

- laboratory analysis (pregnancy test for female patients of childbearing potential, regular hematology and biochemistry, plus glycometabolic state, inflammatory state, lipid and cholesterol spectrum, AGEs, renal function, and neurohormones);
- oral glucose tolerance test (OGTT);

- Visit 1 (T1) at 2 weeks:
  - concomitant medication;
  - NYHA heart failure score;
  - ECG;
  - serious adverse events recording;
  - clinical examination (blood pressure, heart rate, length, weight,);
  - laboratory analysis (pregnancy test for female patients of childbearing potential, regular hematology and biochemistry, plus glycometabolic state, inflammatory state, lipid and cholesterol spectrum, AGEs, renal function, and neurohormones).

- Visit 2 (T2) at 6-8 weeks:
  - concomitant medication;
  - dispense study medication;
  - NYHA heart failure score;
  - ECG;
  - serious adverse events recording;
  - clinical examination (blood pressure, heart rate, length, weight,);
  - laboratory analysis (pregnancy test for female patients of childbearing potential, regular hematology and biochemistry, plus glycometabolic state, inflammatory state, lipid and cholesterol spectrum, AGEs, renal function, and neurohormones).

- Visit 3 (T3) at 4 months:

  - concomitant medication;

  - stop study medication and record the amount of study drug received, dispensed, consumed or administered for drug accountability;

  - NYHA heart failure score;

  - ECG;

  - echocardiography (diastolic function);

  - skin autofluorescence;

  - CMR-scan (primary endpoint);

  - serious adverse events recording;

  - clinical examination (blood pressure, heart rate, length, weight,);

  - laboratory analysis (pregnancy test for female patients of childbearing potential, regular hematology and biochemistry, plus glycometabolic state, inflammatory state, lipid and cholesterol spectrum, AGEs, renal function, and neurohormones);

  - oral glucose tolerance test (OGTT).

- Visit 4 (T4); long-term follow-up (1-year) :

  - concomitant medication;

  - NYHA heart failure score;

  - ECG;

  - serious adverse events recording;

  - clinical examination (blood pressure, heart rate, length, weight,);

  - laboratory analysis

- Visit 5 (T5); long-term follow-up (2-year): visit to outpatient clinic or by telephone

  - concomitant medication;

- NYHA heart failure score;
- serious adverse events recording;
- length, weight.

9.5 Withdrawal of individual subjects

Subjects can leave the study at any time for any reason if they wish to do so without any consequences. The investigator can decide to withdraw.

9.5.1 Specific criteria for withdrawal of individual subjects

Protocol therapy will be discontinued at any time if the following situations occur:

- the development if symptoms which, in the investigator's judgment, preclude further therapy;
- subject withdrew consent;
- lost to follow-up;
- pregnancy;
- study termination;
- the development of severe renal impairment (creatinin > 177 µmol/l).

9.5.2 Replacement of individual subjects after withdrawal

Subjects withdrawn from treatment will not be replaced with other subjects.

9.5.3 Follow-up of subjects withdrawn from treatment

All efforts will be made to follow the patient until the end of the study according to the study schedule. Patients withdrawn from the study will retain their patient number and their medication code already allocated.

10. SAFETY ASPECTS AND SAFETY REPORTING

10.1 Safety aspects

10.1.1 Metformin

To decrease the risk of lactic acidosis, strict prescribing guidelines for metformin therapy have been developed. Absolute contraindications include renal insufficiency (creatinin > 177 µmol/l) and metabolic acidosis. Metabolic acidosis caused by metformin only occurs in case of impaired renal function (creatinin > 177 µmol/l). Therefore, renal function will be monitored. In case of renal function detoriation (creatinin > 177 µmol/l), pH and lactate levels will be determined. Relative contraindications include conditions associated with hypoxemia and hypovolaemia. In addition, the incidence of lactic acidosis has been reported only during chronic administration of metformin (measured in patient-years), which makes it difficult to quantify the risk for lactic acidosis in patients undergoing a specific, high-risk event, such as PCI.

10.1.2 PCI procedure

PCI will be performed according to current European and U.S. guidelines.

10.2 Medical Research Involving Human Subjects Act (WMO) event

In accordance to section 10, subsection 1, of the Medical Research Involving Human Subjects Act (WMO), the investigator will inform the subjects and the reviewing accredited Medical research ethics committee (METC) if anything occurs, on the basis of which it appears that the disadvantages of participation may be significantly greater than was foreseen in the research proposal. The study will be suspended pending further review by the accredited METC, except insofar as suspension would jeopardize the subjects' health. The investigator will take care that all subjects are kept informed.

10.3 Adverse and serious adverse events

Adverse events are defined as any undesirable experience occurring to a subject during a clinical trial, whether or not considered related to the investigational procedure. All adverse events reported spontaneously by the subject or observed by the investigator or his staff will be recorded. Disease related adverse events, which are clearly related to the underlying condition of the patient and are not related to the intervention, will only be reported 6 monthly to the METC.

A serious adverse event is any untoward medical occurrence or effect that:

- results in death;
- is life threatening (at the time of the event);
- requires hospitalization or prolongation of existing inpatients' hospitalization;
- results in persistent or significant disability or incapacity;
- is a new event of the trial likely to affect the safety of the subjects, such as an unexpected outcome.

Since metformin is a widely used medicine which adverse effects have been described in detail, serious adverse events which are common in patients suffering from myocardial infarction which have no known association with the intervention ("disease related adverse events") will be reported 6 monthly to the METC. All serious adverse events with a known association with metformin will be reported to the accredited METC that approved the protocol, according to the requirements of that METC. Serious adverse events that are used to assess safety by the DSMB, which include death, re-MI, target vessel revascularization, worsening renal failure and lactic acidosis will be reported to the METC at all times.

Subjects transferred to other hospitals (referring hospitals) after PCI, which is standard care, will not be considered as an SAE. For logistical reasons, the discharge date from the UMCG will be used to plan the subsequent study visits (T1 – T5). The actual dates that the patients are discharged from the referring hospitals will be collected afterwards and recorded in the database as date of discharge.

10.4 Follow-up of adverse events

All adverse events will be followed until they have abated, or until a stable situation has been reached. Depending on the event additional tests, medical procedures as indicated or referral to a general physician or medical specialist according to current guidelines, possibly resulting in treatment, will take place. Participation in this trial will not interfere with treatment of adverse events.

10.5 Data Safety Monitoring Board

A DSMB is established to perform ongoing safety surveillance and to perform interim analyses on the safety data. This committee is an independent committee, consisting of:

- prof. dr. J.G. Tijssen, PhD, epidemiologist (Chair);
- prof. dr. R.J. de Winter, MD PhD, Interventional cardiologist (member);
- dr. R.M. de Jong, MD PhD, Cardiologist (member);
- drs. A.J. Risselada, MD, hospital pharmacist, epidemiologist (member);
- drs. R.K. Gonera, MD, endocrinologist (member).

These members have no conflict of interest with the sponsors of the study. The DSMB will meet on regular basis (i.e. 2-3 times per year) to evaluate (serious) adverse events, drug safety and clinical endpoints. The charter of the DSMB is added as an attachment (see attachments).

Extending recruitment (based on actual control arm response rates being different to predicted rather than on emerging differences) will be recommended by the DSMB based on the conditional power analysis that will be performed after 280 patient have reached their primary endpoint (LVEF analysis by CMR imaging 4 months after randomization) based on actual control arm response rates being different to predicted rather than on emerging differences. The maximal number of patients to which the inclusion can be extended in this trial will be limited to 450.

10.6 Monitoring

The monitoring of the GIPS-III trial will be performed by the Trial Coordination Center. They will monitor the trial according to the monitor plan; i.e. monitor the database and monitor patient safety according to the current NFU Guidelines (Nederlandse Federatie van Universitair Medische Centra).

10.7 Endpoint Adjudication Committee (EAC)

In order to reduce bias and discrepancies in the judgment of the investigators and to increase accuracy in the assessment of clinical endpoints, an Endpoint Adjucation Committee is established.

All endpoints reported in the GIPS-III trial have to be assessed by the EAC. The EAC has to determine whether the investigators assessment of the endpoint is correct and complete. If not, the EAC will adjudicate the assessment of the investigators. The investigators will have to adopt the assessment by the EAC.

This committee is an independent committee, consisting of:

- Dr. Fred van den Berg, MD, PhD (cardiologist, Chair)
- Dr. Vincent Roolvink, MD, PhD (Interventional cardiologist, member)
- Dr. André van Beek, MD, PhD (internist-endocrinologist)

The assessment of the endpoints is particularly important for the analyses performed by the Data and Safety Monitoring Board (DSMB) of the GIPS-III trial. The DSMB advices the sponsor and steering committee of the GIPS-III trial regarding safety of trial participants and those yet to be recruited. Furthermore, the DSMB advices the sponsor and steering committee of the GIPS-III trial about the validity and scientific merit of the GIPS-III trial.

In order to provide the DSMB with objective and accurate information about endpoints, the investigators of the GIPS-III trial need to provide objective and definitive conclusions on events and

endpoints reached in the trial. The EAC is responsible for the assessment of these endpoints, which the DSMB will use for their analyses.

11.STATISTICAL ANALYSIS AND SAMPLE SIZE

11.1 Statistical analysis

Analyses will be performed according to the intention-to-treat principle for the whole population and in the specified subgroups. An analysis per protocol will also be performed. Differences between group means will be assessed with the two-tailed Student's t-test. Chi-square analysis or Fisher's exact test is used to test differences between proportions. Survival will be calculated by the Kaplan-Meier product-limit method. The Mantel-Cox (or log-rank) test will be used to evaluate differences in survival between the two treatment groups. The Cox proportional-hazards regression model will be used to calculate relative risks and to adjust for differences in baseline characteristics. The relationship between metformin use and the primary and secondary endpoints will be studied using linear regression analysis, in which the outcome at the time of determination will be adjusted for the outcome at baseline. In addition, to assess the relationship between the determinant and the outcome over multiple measurements in time, GEE (generalized estimation equations) will be used as a method for dealing with correlated data arising from repeated measurements. Statistical significance is considered as a two-tailed p value <0.05. The Statistical Package for the Social Sciences (SPSS Inc., Chicago, IL, USA) version 16.0 will be used for all statistical analysis.

11.3 Sample size

The study is powered for the supposed change caused by the intervention in global left ventricular ejection fraction between the intervention group and placebo group at 4 months after hospitalization. This trial is primarily intended as a proof of concept study, and therefore not powered for a mortality benefit, but for the surrogate endpoint difference in left ventricular ejection fraction measured with CMR imaging 4 months after primary PCI. Van der Vleuten et al (8) demonstrated that a decrease of 5% in LVEF after STEMI leads to a HR of 1.44 for mortality at 1 year follow-up. The recently published HEBE III trial assumed a 3% change in LVEF to be clinical relevant

and to be feasible. Therefore a difference of 3% in LVEF is a relevant primary endpoint. With 141 patients in each study group, the study has 80% power to detect a 3% difference in change in ejection fraction between active treatment and control, assuming a 2-sided α of .05 for the change in left ventricular ejection fraction.

11.3.1 Sample size calculation

The following formula was used to assess the sample size (N) per group. The entire sample size will therefore be 2N.

$$N = (A + B)^2 \times 2(\sigma^2 / \delta^2)$$

N =	sample size of 1 group (Total sample = 2N)	?
A =	significance level (5%=1,96)	1,96
B =	power (80%=0,84)	0,84
σ =	expected standard deviation	9
δ =	delta (clinical relevant difference)	3

This results in the following calculation:

$$N = (1,96 + 0,84)^2 \times 2(9^2 / 3^2)$$

$$N = (2,8)^2 \times 18$$

$$N = 7,84 \times 18$$

$$N = 141$$

Sample size (2N) = 282

We assumed that in 12% of patients a primary endpoint observation would not be obtained. However, actual rates of patients not undergoing cardiac MRI (primary endpoint) differ from that assumption. Actual rates are close to 26%, therefore we require an increase to 380 patients , assuming that in 26% of patients no primary efficacy measurement will be obtained.

12. ETHICAL CONSIDERATIONS

12.1 Regulation statement

The study will be conducted according to the principles of the Declaration of Helsinki (2008, Seoul) and in accordance with the Medical Research Involving Human Subjects Act (WMO).

12.2 Informed consent

The purpose and details of the study are explained to all patients by a cardiologist and their verbal consent will be obtained prior to participation in the study by a cardiologist. Patients are enrolled in an emergency situation, and therefore consent is given verbally in the presence of an independent witness. Because of the acute situation, there is limited time for the patient to consider participation. If the patient expresses any doubt considering participation, the cardiologist will not enroll the patient. Written informed consent has to be given by the patient as soon as the clinical situation of the patient allows it.

The patient information letter and informed consent form is attached (see attachments).

12.3 Insurance

The Onderlinge Waarborgmaatschappij Centramed, Postbus 191, 2270 AD VOORBURG (polis number 624.529.102) has a liability insurance which is in accordance with the legal requirements in the Netherlands (Article 7 of the WMO and the Measure regarding Compulsory Insurance for Clinical Research in Humans of June 23th, 2003) This insurance provides cover for damage to research subjects through injury or death caused by the study procedure:

1. €450.000,- (i.e. four hundred and fifty thousand Euro) for death or injury for each subject who participates in the Research caused by study procedure;
2. €3.500.000 (i.e. three million five hundred thousand Euro) for death or injury for all subjects who participate in the Research caused by study procedure;

3. €5.000.000 (i.e. five million Euro) for the total damage incurred by the organization for all damage disclosed by scientific research.

The insurance applies to the damage that becomes apparent during the study or within 4 years after the end of the study.

12.4 Incentives

Patients will not receive any special incentives, compensation or treatment through participation in the study, with the exception of travel expenses if applicable for a specific study subject.

13. ADMINISTRATIVE ASPECTS

13.1 Handling and storage of data and documents

For each randomized patient, a electronic Case Record Form (eCRF) for data recording is provided. CRFs are coded with a study code combined with a patient number, which will be used in ascending numerical order. The Oracle Clinical electronic Case Record Form will be used for the GIPS-III trial. All data will be recorded in a dedicated database.

The investigator will ensure that patient anonymity is maintained according to the Personal Data Protection Act (WBP). On CRFs or other documents, patients are not identified by their names but by the CRF code. Only the investigators and co-investigators will have access to the CRF code. The coordinating investigator will keep a separate log of patient codes, names and addresses.

13.2 Subsidizing party

This trial is subsidized by the Van Buchem Stichting and by grant 95103007 by the ZonMw, the Netherlands Organization for Health Research and Development, The Hague, the Netherlands.

13.3 Amendments

All substantial amendments will be sent for approval to the METC that gave a favorable opinion. A substantial amendment is defined as an amendment to the terms of the METC application, or to the protocol or any other supporting documentation, that is likely to affect to a significant degree:

- the safety or physical or mental integrity of the subjects of the trial;
- the scientific value of the trial;
- the conduct or management of the trial;
- the quality or safety of any intervention used in the trial.

Non-substantial amendments will not be notified to the accredited METC, but will be recorded and filed by the sponsor.

13.4 Progress report

Information will be provided to the METC once a year on the date of inclusion of the first subject, numbers of subjects included and numbers of subjects that have completed the trial, serious adverse events/ serious adverse reactions, other problems, and amendments.

13.5 End of study report

The investigator will notify the accredited METC of the end of the study within a period of 8 weeks. The end of the study is defined as the last patient's last visit.

Within one year after the end of the study, the investigators will submit a final study report with the results of the study, including any publications/abstracts of the study, to the accredited METC.

13.6 Publication policy

Investigators will be encouraged to publish and present the results of the study. All publications will be reviewed and approved by the Executive Committee. The Executive Committee consists of the following members: I.C.C. van der Horst (MD, PhD), C.P.H. Lexis (MD) and D.J. van Veldhuisen (MD, PhD).

Investigators and the Executive Committee will publish the study results in compliance with the CCMO publication policy.

14.APPENDIX

14.1. NYHA heart failure classification

Class I: patients with no limitation of activities; they suffer no symptoms from ordinary activities.

Class II: patients with slight, mild limitation of activity; they are comfortable with rest or with mild exertion.

Class III: patients with marked limitations of activity; they are comfortable only at rest.

Class IV: patients who should be at complete rest, confined to bed or chair; any physical activity brings on discomfort and symptoms occur at rest.

14.2 Current guidelines on diagnosis of diabetes and prediabetes

Diabetes Mellitus

- HbA1c ≥ 6.5% or;
- Fasting glucose ≥ 7.0 mmol/L or;
- OGTT glucose ≥ 11.1 mmol/L.

  (measured at 120 minutes post glucose drink)

Prediabetes

- HbA1c 5.7-6.4% or
- Fasting glucose 5.6-6.9 mmol/L (venous) (ADA) : Impaired fasting glucose (IFG);
- OGTT glucose 7.9-11.0 mmol/L (venous), WHO): Impaired glucose tolerance (IGT).

  (measured at 120 minutes post glucose drink)

15. REFERENCES

Reference List

(1) Flaherty JD, Davidson CJ. Diabetes and coronary revascularization. JAMA 2005 Mar 23;293(12):1501-8.

(2) Bonow RO, Gheorghiade M. The diabetes epidemic: a national and global crisis. Am J Med 2004 Mar 8;116 Suppl 5A:2S-10S.

(3) Ryden L, Standl E, Bartnik M, Van den Berghe G, Betteridge J, de Boer MJ, et al. Guidelines on diabetes, pre-diabetes, and cardiovascular diseases: executive summary. The Task Force on Diabetes and Cardiovascular Diseases of the European Society of Cardiology (ESC) and of the European Association for the Study of Diabetes (EASD). Eur Heart J 2007 Jan;28(1):88-136.

(4) Aguilar D, Solomon SD, Kober L, Rouleau JL, Skali H, McMurray JJ, et al. Newly diagnosed and previously known diabetes mellitus and 1-year outcomes of acute myocardial infarction: the VALsartan In Acute myocardial iNfarcTion (VALIANT) trial. Circulation 2004 Sep 21;110(12):1572-8.

(5) The final 10-year follow-up results from the BARI randomized trial. J Am Coll Cardiol 2007 Apr 17;49(15):1600-6.

(6) Hofsten DE, Logstrup BB, Moller JE, Pellikka PA, Egstrup K. Abnormal glucose metabolism in acute myocardial infarction: influence on left ventricular function and prognosis. JACC Cardiovasc Imaging 2009 May;2(5):592-9.

(7) Timmer JR, Ottervanger JP, Thomas K, Hoorntje JC, de Boer MJ, Suryapranata H, et al. Long-term, cause-specific mortality after myocardial infarction in diabetes. Eur Heart J 2004 Jun;25(11):926-31.

(8) van der Vleuten PA, Rasoul S, Huurnink W, van der Horst IC, Slart RH, Reiffers S, et al. The importance of left ventricular function for long-term outcome after primary percutaneous coronary intervention. BMC Cardiovasc Disord 2008;8:4.

(9) Aronow WS, Ahn C. Incidence of heart failure in 2,737 older persons with and without diabetes mellitus. Chest 1999 Mar;115(3):867-8.

(10) Nichols GA, Koro CE, Gullion CM, Ephross SA, Brown JB. The incidence of congestive heart failure associated with antidiabetic therapies. Diabetes Metab Res Rev 2005 Jan;21(1):51-7.

(11) Timmer JR, van der Horst IC, Ottervanger JP, Henriques JP, Hoorntje JC, de Boer MJ, et al. Prognostic value of admission glucose in non-diabetic patients with myocardial infarction. Am Heart J 2004 Sep;148(3):399-404.

(12) Kosiborod M, Inzucchi SE, Krumholz HM, Xiao L, Jones PG, Fiske S, et al. Glucometrics in patients hospitalized with acute myocardial infarction: defining the optimal outcomes-based measure of risk. Circulation 2008 Feb 26;117(8):1018-27.

(13) Stone PH, Muller JE, Hartwell T, York BJ, Rutherford JD, Parker CB, et al. The effect of diabetes mellitus on prognosis and serial left ventricular function after acute myocardial infarction: contribution of both coronary disease and diastolic left ventricular dysfunction to the adverse prognosis. The MILIS Study Group. J Am Coll Cardiol 1989 Jul;14(1):49-57.

(14) Heerebeek van L, Hamdani N, Handoko ML, Falcao-Pires I, Musters RJ, Kupreishvili K, et al. Diastolic stiffness of the failing diabetic heart: importance of fibrosis, advanced glycation end products, and myocyte resting tension. Circulation 2008 Jan 1;117(1):43-51.

(15) Hillege HL, Nitsch D, Pfeffer MA, Swedberg K, McMurray JJ, Yusuf S, et al. Renal function as a predictor of outcome in a broad spectrum of patients with heart failure. Circulation 2006 Feb 7;113(5):671-8.

(16) Damman K, Navis G, Voors AA, Asselbergs FW, Smilde TD, Cleland JG, et al. Worsening renal function and prognosis in heart failure: systematic review and meta-analysis. J Card Fail 2007 Oct;13(8):599-608.

(17) Damman K, Jaarsma T, Voors AA, Navis G, Hillege HL, van Veldhuisen DJ. Both in- and out-hospital worsening of renal function predict outcome in patients with heart failure: results from the Coordinating Study Evaluating Outcome of Advising and Counseling in Heart Failure (COACH). Eur J Heart Fail 2009 Sep;11(9):847-54.

(18) Smilde TD, van Veldhuisen DJ, Navis G, Voors AA, Hillege HL. Drawbacks and prognostic value of formulas estimating renal function in patients with chronic heart failure and systolic dysfunction. Circulation 2006 Oct 10;114(15):1572-80.

(19) Nagi DK, Yudkin JS. Effects of metformin on insulin resistance, risk factors for cardiovascular disease, and plasminogen activator inhibitor in NIDDM subjects. A study of two ethnic groups. Diabetes Care 1993 Apr;16(4):621-9.

(20) Hunt SA, Abraham WT, Chin MH, Feldman AM, Francis GS, Ganiats TG, et al. 2009 Focused update incorporated into the ACC/AHA 2005 Guidelines for the Diagnosis and Management of Heart Failure in Adults A Report of the American College of Cardiology Foundation/American Heart Association Task Force on Practice Guidelines Developed in Collaboration With the International Society for Heart and Lung Transplantation. J Am Coll Cardiol 2009 Apr 14;53(15):e1-e90.

(21) Effect of intensive blood-glucose control with metformin on complications in overweight patients with type 2 diabetes (UKPDS 34). UK Prospective Diabetes Study (UKPDS) Group. Lancet 1998 Sep 12;352(9131):854-65.

(22) Kirpichnikov D, McFarlane SI, Sowers JR. Metformin: an update. Ann Intern Med 2002 Jul 2;137(1):25-33.

(23) Lalau JD, Race JM. Lactic acidosis in metformin therapy: searching for a link with metformin in reports of 'metformin-associated lactic acidosis'. Diabetes Obes Metab 2001 Jun;3(3):195-201.

(24) Malmberg K, Ryden L, Wedel H, Birkeland K, Bootsma A, Dickstein K, et al. Intense metabolic control by means of insulin in patients with diabetes mellitus and acute myocardial infarction (DIGAMI 2): effects on mortality and morbidity. Eur Heart J 2005 Apr;26(7):650-61.

(25) Mehta SR, Yusuf S, Diaz R, Zhu J, Pais P, Xavier D, et al. Effect of glucose-insulin-potassium infusion on mortality in patients with acute ST-segment elevation myocardial infarction: the CREATE-ECLA randomized controlled trial. JAMA 2005 Jan 26;293(4):437-46.

(26) Timmer JR, Svilaas T, Ottervanger JP, Henriques JP, Dambrink JH, van den Broek SA, et al. Glucose-insulin-potassium infusion in patients with acute myocardial infarction without signs of heart failure: the Glucose-Insulin-Potassium Study (GIPS)-II. J Am Coll Cardiol 2006 Apr 18;47(8):1730-1.

(27) van der Horst IC, Zijlstra F, 't Hof AW, Doggen CJ, de Boer MJ, Suryapranata H, et al. Glucose-insulin-potassium infusion inpatients treated with primary angioplasty for acute myocardial infarction: the glucose-insulin-potassium study: a randomized trial. J Am Coll Cardiol 2003 Sep 3;42(5):784-91.

(28) Cheung NW, Wong VW, McLean M. The Hyperglycemia: Intensive Insulin Infusion in Infarction (HI-5) study: a randomized controlled trial of insulin infusion therapy for myocardial infarction. Diabetes Care 2006 Apr;29(4):765-70.

(29) Anselmino M, Bartnik M, Malmberg K, Ryden L. Management of coronary artery disease in patients with and without diabetes mellitus. Acute management reasonable but secondary prevention unacceptably poor: a report from the Euro Heart Survey on Diabetes and the Heart. Eur J Cardiovasc Prev Rehabil 2007 Feb;14(1):28-36.

(30) McAlister FA, Eurich DT, Majumdar SR, Johnson JA. The risk of heart failure in patients with type 2 diabetes treated with oral agent monotherapy. Eur J Heart Fail 2008 Jul;10(7):703-8.

(31) Eurich DT, McAlister FA, Blackburn DF, Majumdar SR, Tsuyuki RT, Varney J, et al. Benefits and harms of antidiabetic agents in patients with diabetes and heart failure: systematic review. BMJ 2007 Sep 8;335(7618):497.

(32) Eurich DT, Majumdar SR, McAlister FA, Tsuyuki RT, Johnson JA. Improved clinical outcomes associated with metformin in patients with diabetes and heart failure. Diabetes Care 2005 Oct;28(10):2345-51.

(33) Salpeter SR, Buckley NS, Kahn JA, Salpeter EE. Meta-analysis: metformin treatment in persons at risk for diabetes mellitus. Am J Med 2008 Feb;121(2):149-57.

(34) Gundewar S, Calvert JW, Jha S, Toedt-Pingel I, Ji SY, Nunez D, et al. Activation of AMP-activated protein kinase by metformin improves left ventricular function and survival in heart failure. Circ Res 2009 Feb 13;104(3):403-11.

(35) Calvert JW, Gundewar S, Jha S, Greer JJ, Bestermann WH, Tian R, et al. Acute metformin therapy confers cardioprotection against myocardial infarction via AMPK-eNOS-mediated signaling. Diabetes 2008 Mar;57(3):696-705.

(36) Solskov L, Lofgren B, Kristiansen SB, Jessen N, Pold R, Nielsen TT, et al. Metformin induces cardioprotection against ischaemia/reperfusion injury in the rat heart 24 hours after administration. Basic Clin Pharmacol Toxicol 2008 Jul;103(1):82-7.

(37) Morrow VA, Foufelle F, Connell JM, Petrie JR, Gould GW, Salt IP. Direct activation of AMP-activated protein kinase stimulates nitric-oxide synthesis in human aortic endothelial cells. J Biol Chem 2003 Aug 22;278(34):31629-39.

(38) Kudo N, Gillespie JG, Kung L, Witters LA, Schulz R, Clanachan AS, et al. Characterization of 5'AMP-activated protein kinase activity in the heart and its role in inhibiting acetyl-CoA carboxylase during reperfusion following ischemia. Biochim Biophys Acta 1996 May 31;1301(1-2):67-75.

(39) Russell RR, III, Bergeron R, Shulman GI, Young LH. Translocation of myocardial GLUT-4 and increased glucose uptake through activation of AMPK by AICAR. Am J Physiol 1999 Aug;277(2 Pt 2):H643-H649.

(40) Marsin AS, Bertrand L, Rider MH, Deprez J, Beauloye C, Vincent MF, et al. Phosphorylation and activation of heart PFK-2 by AMPK has a role in the stimulation of glycolysis during ischaemia. Curr Biol 2000 Oct 19;10(20):1247-55.

(41) Winder WW, Hardie DG. AMP-activated protein kinase, a metabolic master switch: possible roles in type 2 diabetes. Am J Physiol 1999 Jul;277(1 Pt 1):E1-10.

(42) Kimura N, Tokunaga C, Dalal S, Richardson C, Yoshino K, Hara K, et al. A possible linkage between AMP-activated protein kinase (AMPK) and mammalian target of rapamycin (mTOR) signalling pathway. Genes Cells 2003 Jan;8(1):65-79.

(43) Lund SS, Tarnow L, Stehouwer CD, Schalkwijk CG, Teerlink T, Gram J, et al. Impact of metformin versus repaglinide on non-glycaemic cardiovascular risk markers related to inflammation and endothelial dysfunction in non-obese patients with type 2 diabetes. Eur J Endocrinol 2008 May;158(5):631-41.

(44) Thompson SG, Kienast J, Pyke SD, Haverkate F, van de Loo JC. Hemostatic factors and the risk of myocardial infarction or sudden death in patients with angina pectoris. European Concerted Action on Thrombosis and Disabilities Angina Pectoris Study Group. N Engl J Med 1995 Mar 9;332(10):635-41.

(45) Ridker PM, Rifai N, Rose L, Buring JE, Cook NR. Comparison of C-reactive protein and low-density lipoprotein cholesterol levels in the prediction of first cardiovascular events. N Engl J Med 2002 Nov 14;347(20):1557-65.

(46) Ridker PM, Rifai N, Pfeffer M, Sacks F, Lepage S, Braunwald E. Elevation of tumor necrosis factor-alpha and increased risk of recurrent coronary events after myocardial infarction. Circulation 2000 May 9;101(18):2149-53.

(47) Lapolla A, Piarulli F, Sartore G, Rossetti C, Martano L, Carraro P, et al. Peripheral artery disease in type 2 diabetes: the role of fibrinolysis. Thromb Haemost 2003 Jan;89(1):91-6.

(48) Tanne D, Haim M, Boyko V, Goldbourt U, Reshef T, Matetzky S, et al. Soluble intercellular adhesion molecule-1 and risk of future ischemic stroke: a nested case-control study from the Bezafibrate Infarction Prevention (BIP) study cohort. Stroke 2002 Sep;33(9):2182-6.

(49) Blankenberg S, Rupprecht HJ, Bickel C, Peetz D, Hafner G, Tiret L, et al. Circulating cell adhesion molecules and death in patients with coronary artery disease. Circulation 2001 Sep 18;104(12):1336-42.

(50) Schulze F, Lenzen H, Hanefeld C, Bartling A, Osterziel KJ, Goudeva L, et al. Asymmetric dimethylarginine is an independent risk factor for coronary heart disease: results from the multicenter Coronary Artery Risk Determination investigating the Influence of ADMA Concentration (CARDIAC) study. Am Heart J 2006 Sep;152(3):493-8.

(51) Schulze MB, Shai I, Rimm EB, Li T, Rifai N, Hu FB. Adiponectin and future coronary heart disease events among men with type 2 diabetes. Diabetes 2005 Feb;54(2):534-9.

(52) Siroen MP, Teerlink T, Nijveldt RJ, Prins HA, Richir MC, van Leeuwen PA. The clinical significance of asymmetric dimethylarginine. Annu Rev Nutr 2006;26:203-28.

(53) Stehouwer CD, Gall MA, Twisk JW, Knudsen E, Emeis JJ, Parving HH. Increased urinary albumin excretion, endothelial dysfunction, and chronic low-grade inflammation in type 2 diabetes: progressive, interrelated, and independently associated with risk of death. Diabetes 2002 Apr;51(4):1157-65.

(54) Araujo JP, Lourenco P, Azevedo A, Frioes F, Rocha-Goncalves F, Ferreira A, et al. Prognostic value of high-sensitivity C-reactive protein in heart failure: a systematic review. J Card Fail 2009 Apr;15(3):256-66.

(55) de Jager J, Kooy A, Lehert P, Bets D, Wulffele MG, Teerlink T, et al. Effects of short-term treatment with metformin on markers of endothelial function and inflammatory activity in type 2 diabetes mellitus: a randomized, placebo-controlled trial. J Intern Med 2005 Jan;257(1):100-9.

(56) Hartog JW, Voors AA, Bakker SJ, Smit AJ, van Veldhuisen DJ. Advanced glycation end-products (AGEs) and heart failure: pathophysiology and clinical implications. Eur J Heart Fail 2007 Dec;9(12):1146-55.

(57) Meerwaldt R, Lutgers HL, Links TP, Graaff R, Baynes JW, Gans RO, et al. Skin autofluorescence is a strong predictor of cardiac mortality in diabetes. Diabetes Care 2007 Jan;30(1):107-12.

(58) Monnier VM, Bautista O, Kenny D, Sell DR, Fogarty J, Dahms W, et al. Skin collagen glycation, glycoxidation, and crosslinking are lower in subjects with long-term intensive versus conventional therapy of type 1 diabetes: relevance of glycated collagen products versus HbA1c as markers of diabetic complications. DCCT Skin Collagen Ancillary Study Group. Diabetes Control and Complications Trial. Diabetes 1999 Apr;48(4):870-80.

(59) Aronson D. Cross-linking of glycated collagen in the pathogenesis of arterial and myocardial stiffening of aging and diabetes. J Hypertens 2003 Jan;21(1):3-12.

(60) Krum H, Liew D. New developments in the pharmacological treatment of chronic heart failure. Expert Opin Investig Drugs 2003 May;12(5):751-7.

(61) Bakris GL, Bank AJ, Kass DA, Neutel JM, Preston RA, Oparil S. Advanced glycation end-product cross-link breakers. A novel approach to cardiovascular pathologies related to the aging process. Am J Hypertens 2004 Dec;17(12 Pt 2):23S-30S.

(62) Zieman SJ, Kass DA. Advanced glycation endproduct crosslinking in the cardiovascular system: potential therapeutic target for cardiovascular disease. Drugs 2004;64(5):459-70.

(63) Tanaka Y, Uchino H, Shimizu T, Yoshii H, Niwa M, Ohmura C, et al. Effect of metformin on advanced glycation endproduct formation and peripheral nerve function in streptozotocin-induced diabetic rats. Eur J Pharmacol 1999 Jul 2;376(1-2):17-22.

(64) Jyothirmayi GN, Soni BJ, Masurekar M, Lyons M, Regan TJ. Effects of Metformin on Collagen Glycation and Diastolic Dysfunction in Diabetic Myocardium. J Cardiovasc Pharmacol Ther 1998 Oct;3(4):319-26.

(65) Ouslimani N, Mahrouf M, Peynet J, Bonnefont-Rousselot D, Cosson C, Legrand A, et al. Metformin reduces endothelial cell expression of both the receptor for advanced glycation end products and lectin-like oxidized receptor 1. Metabolism 2007 Mar;56(3):308-13.

(66) Bhamra GS, Hausenloy DJ, Davidson SM, Carr RD, Paiva M, Wynne AM, et al. Metformin protects the ischemic heart by the Akt-mediated inhibition of mitochondrial permeability transition pore opening. Basic Res Cardiol 2008 May;103(3):274-84.

(67) Davidson SM, Hausenloy D, Duchen MR, Yellon DM. Signalling via the reperfusion injury signalling kinase (RISK) pathway links closure of the mitochondrial permeability transition pore to cardioprotection. Int J Biochem Cell Biol 2006 Mar;38(3):414-9.

(68) Hausenloy DJ, Yellon DM, Mani-Babu S, Duchen MR. Preconditioning protects by inhibiting the mitochondrial permeability transition. Am J Physiol Heart Circ Physiol 2004 Aug;287(2):H841-H849.

(69) Abu-Hamdah R, Rabiee A, Meneilly GS, Shannon RP, Andersen DK, Elahi D. Clinical review: The extrapancreatic effects of glucagon-like peptide-1 and related peptides. J Clin Endocrinol Metab 2009 Jun;94(6):1843-52.

(70) Cuthbertson J, Patterson S, O'Harte FP, Bell PM. Investigation of the effect of oral metformin on dipeptidylpeptidase-4 (DPP-4) activity in Type 2 diabetes. Diabet Med 2009 Jun;26(6):649-54.

(71) Kooy A, de Jager J, Lehert P, Bets D, Wulffele MG, Donker AJ, et al. Long-term effects of metformin on metabolism and microvascular and macrovascular disease in patients with type 2 diabetes mellitus. Arch Intern Med 2009 Mar 23;169(6):616-25.

(72) Foley DP, Escaned J, Strauss BH, di Mario C, Haase J, Keane D, et al. Quantitative coronary angiography (QCA) in interventional cardiology: clinical application of QCA measurements. Prog Cardiovasc Dis 1994 Mar;36(5):363-84.

(73) The Thrombolysis in Myocardial Infarction (TIMI) trial. Phase I findings. TIMI Study Group. N Engl J Med 1985 Apr 4;312(14):932-6.

(74) 't Hof AW, Liem A, Suryapranata H, Hoorntje JC, de Boer MJ, Zijlstra F. Angiographic assessment of myocardial reperfusion in patients treated with primary angioplasty for acute myocardial infarction: myocardial blush grade. Zwolle Myocardial Infarction Study Group. Circulation 1998 Jun 16;97(23):2302-6.

(75) Vogelzang M, Vlaar PJ, Svilaas T, Amo D, Nijsten MW, Zijlstra F. Computer-assisted myocardial blush quantification after percutaneous coronary angioplasty for acute myocardial infarction: a substudy from the TAPAS trial. Eur Heart J 2009 Mar;30(5):594-9.

(76) van der Laarse A, Vermeer F, Hermens WT, Willems GM, de Neef K, Simoons ML, et al. Effects of early intracoronary streptokinase on infarct size estimated from cumulative enzyme release and on enzyme release rate: a randomized trial of 533 patients with acute myocardial infarction. Am Heart J 1986 Oct;112(4):672-81.

(77) Elsman P, Zijlstra F, Miedema K, Hoorntje JC, Dikkeschei LD, Slingerland RJ, et al. The predictive value of cumulative lactate dehydrogenase release within the first 72 h of acute myocardial infarction in patients treated with primary angioplasty. Ann Clin Biochem 2004 Mar;41(Pt 2):142-8.

(78) Meerwaldt R, Graaff R, Oomen PH, Links TP, Jager JJ, Alderson NL, et al. Simple non-invasive assessment of advanced glycation endproduct accumulation. Diabetologia 2004 Jul;47(7):1324-30.

16. ATTACHMENTS
