# Supplementary material for: Sex differences in cardiac structure and function following ST-segment elevation myocardial infarction
Source: Sci Rep. 2026 May 19;16:22756. doi: 10.1038/s41598-026-52993-8 (PMC13385960; doi:10.1038/s41598-026-52993-8)
Supplement: Supplementary file 5 — Supplementary Material 5 [file 41598_2026_52993_MOESM5_ESM.docx]

**CONSORT 2025 checklist of information to include when reporting a randomised trial***

| **Section / Topic** | **No** | **CONSORT 2025 checklist item description** | **Reported on page no.** |
| --- | --- | --- | --- |
| **Title and abstract** | | |  |
| Title and structured abstract | 1a | Identification as a randomised trial | Reported in primary publication (Ref. 20) |
|  | 1b | Structured summary of the trial design, methods, results, and conclusions | 2, Reported in primary publication (Ref. 20) |
| **Open science** | | |  |
| Trial registration | 2 | Name of trial registry, identifying number (with URL) and date of registration | 5, Reported in design paper (Ref. 19) and primary publication (Ref. 20) |
| Protocol and statistical analysis plan | 3 | Where the trial protocol and statistical analysis plan can be accessed | Reported in design paper (Ref. 19) |
| Data sharing | 4 | Where and how the individual de-identified participant data (including data dictionary), statistical code and any other materials can be accessed | 25 |
| Funding and conflicts of interest | 5a | Sources of funding and other support (e.g., supply of drugs), and role of funders in the design, conduct, analysis and reporting of the trial | 24, Reported in primary publication (Ref. 20) |
|  | 5b | Financial and other conflicts of interest of the manuscript authors | 24, Reported in primary publication (Ref. 20) |
| **Introduction** | | |  |
| Background and rationale | 6 | Scientific background and rationale | 4, Reported in design paper (Ref. 19) and primary publication (Ref. 20) |
| Objectives | 7 | Specific objectives related to benefits and harms | Reported in design paper (Ref. 19) and primary publication (Ref. 20) |
| **Methods** | | |  |
| Patient and public involvement | 8 | Details of patient or public involvement in the design, conduct and reporting of the trial | 6 |
| Trial design | 9 | Description of trial design including type of trial (e.g., parallel group, crossover), allocation ratio, and framework (e.g., superiority, equivalence, non-inferiority, exploratory) | Reported in design paper (Ref. 19) and primary publication (Ref. 20) |
| Changes to trial protocol | 10 | Important changes to the trial after it commenced including any outcomes or analyses that were not prespecified, with reason | Reported in primary publication (Ref. 20) |
| Trial setting | 11 | Settings (e.g., community, hospital) and locations (e.g., countries, sites) where the trial was conducted | Reported in design paper (Ref. 19) and primary publication (Ref. 20) |
| Eligibility criteria | 12a | Eligibility criteria for participants | 5, Reported in design paper (Ref. 19) and primary publication (Ref. 20) |
|  | 12b | If applicable, eligibility criteria for sites and for individuals delivering the interventions (e.g., surgeons, physiotherapists) | Not applicable |
| Intervention and comparator | 13 | Intervention and comparator with sufficient details to allow replication. If relevant, where additional materials describing the intervention and comparator (e.g., intervention manual) can be accessed | Reported in design paper (Ref. 19) and primary publication (Ref. 20) |
| Outcomes | 14 | Pre-specified primary and secondary outcomes, including the specific measurement variable (e.g., systolic blood pressure), analysis metric (e.g., change from baseline, final value, time to event), method of aggregation (e.g., median, proportion), and time point for each outcome | 6-8, Reported in design paper (Ref. 19) and primary publication (Ref. 20) |
| Harms | 15 | How harms were defined and assessed (e.g., systematically, non-systematically) | Reported in design paper (Ref. 19) and primary publication (Ref. 20) |
| Sample size | 16a | How sample size was determined, including all assumptions supporting the sample size calculation | Reported in design paper (Ref. 19) and primary publication (Ref. 20) |
|  | 16b | Explanation of any interim analyses and stopping guidelines | Reported in design paper (Ref. 19) |
| Randomisation: |  |  |  |
| Sequence generation | 17a | Who generated the random allocation sequence and the method used | Reported in design paper (Ref. 19) and primary publication (Ref. 20) |
|  | 17b | Type of randomisation and details of any restriction (e.g., stratification, blocking and block size) | Reported in design paper (Ref. 19) and primary publication (Ref. 20) |
| Allocation concealment mechanism | 18 | Mechanism used to implement the random allocation sequence (e.g., central computer/telephone; sequentially numbered, opaque, sealed containers), describing any steps to conceal the sequence until interventions were assigned | Reported in design paper (Ref. 19) and primary publication (Ref. 20) |
| Implementation | 19 | Whether the personnel who enrolled and those who assigned participants to the interventions had access to the random allocation sequence | Reported in design paper (Ref. 19) and primary publication (Ref. 20) |
| Blinding | 20a | Who was blinded after assignment to interventions (e.g., participants, care providers, outcome assessors, data analysts) | Reported in design paper (Ref. 19) and primary publication (Ref. 20) |
|  | 20b | If blinded, how blinding was achieved and description of the similarity of interventions | Reported in design paper (Ref. 19) and primary publication (Ref. 20) |
| Statistical methods | 21a | Statistical methods used to compare groups for primary and secondary outcomes, including harms | 8, Reported in design paper (Ref. 19) and primary publication (Ref. 20) |
|  | 21b | Definition of who is included in each analysis (e.g., all randomised participants), and in which group | 5,9, Reported in primary publication (Ref. 20) |
|  | 21c | How missing data were handled in the analysis | 5, 9, Reported in primary publication (Ref. 20) |
|  | 21d | Methods for any additional analyses (e.g., subgroup and sensitivity analyses), distinguishing prespecified from post-hoc | Reported in primary publication (Ref. 20) |
| **Results** | | |  |
| Participant flow, including flow diagram | 22a | For each group, the numbers of participants who were randomly assigned, received intended intervention, and were analysed for the primary outcome | Reported in primary publication (Ref. 20) |
|  | 22b | For each group, losses and exclusions after randomisation, together with reasons | Reported in primary publication (Ref. 20) |
| Recruitment | 23a | Dates defining the periods of recruitment and follow-up for outcomes of benefits and harms | Reported in primary publication (Ref. 20) |
|  | 23b | If relevant, why the trial ended or was stopped | Not applicable |
| Intervention and comparator delivery | 24a | Intervention and comparator as they were actually administered (e.g., where appropriate, who delivered the intervention/comparator, how participants adhered, whether they were delivered as intended [fidelity]) | Reported in primary publication (Ref. 20) |
|  | 24b | Concomitant care received during the trial for each group | Reported in primary publication (Ref. 20) |
| Baseline data | 25 | A table showing baseline demographic and clinical characteristics for each group | 10-12, Reported in primary publication (Ref. 20) |
| Numbers analysed,  outcomes and estimation | 26 | For each primary and secondary outcome, by group:   - the number of participants included in the analysis - the number of participants with available data at the outcome time point - result for each group, and the estimated effect size and its precision (such as 95% confidence interval) - for binary outcomes, presentation of both absolute and relative effect size | 9, Reported in primary publication (Ref. 20) |
| Harms | 27 | All harms or unintended events in each group | Reported in primary publication (Ref. 20) |
| Ancillary analyses | 28 | Any other analyses performed, including subgroup and sensitivity analyses, distinguishing pre-specified from post-hoc | Reported in primary publication (Ref. 20) |
| **Discussion** | | |  |
| Interpretation | 29 | Interpretation consistent with results, balancing benefits and harms, and considering other relevant evidence | 18-22, Reported in primary publication (Ref. 20) |
| Limitations | 30 | Trial limitations, addressing sources of potential bias, imprecision, generalisability, and, if relevant, multiplicity of analyses | 20, Reported in primary publication (Ref. 20) |

*We strongly recommend reading this statement in conjunction with the CONSORT 2025 Explanation and Elaboration and/or the CONSORT 2025 Expanded Checklist for important clarifications on all the items. We also recommend reading relevant CONSORT extensions. See [www.consort-spirit.org](http://www.consort-spirit.org).

Citation: Hopewell S, Chan AW, Collins GS, Hróbjartsson A, Moher D, Schulz KF, et al. CONSORT 2025 Statement: updated guideline for reporting randomised trials. BMJ. 2025; 388:e081123. <https://dx.doi.org/10.1136/bmj-2024-081123>.

© 2025 Hopewell et al. This is an Open Access article distributed under the terms of the Creative Commons Attribution License (<https://creativecommons.org/licenses/by/4.0/>), which permits unrestricted use, distribution, and reproduction in any medium, provided the original work is properly cited.
